# Supplementary material for: Allocentric flocking
Source: Nat Commun. 2025 Oct 13;16:9051. doi: 10.1038/s41467-025-64676-5 (PMC12518777; doi:10.1038/s41467-025-64676-5)
Supplement: Supplementary file 1 — Supplementary Information [file 41467_2025_64676_MOESM1_ESM.pdf]

Supplementary Information for
Allocentric Flocking

Mohammad Salahshour & Iain D. Couzin

Contents

Supplementary Note 1 The spontaneous network dynamics in the spin system model 3

Supplementary Note 2 Individual motion in the spin system model 3

Supplementary Note 3 Individual information acquisition in the spin system model 4
Supplementary Note 3.1 decision-making speed: Reaching the target. 5

Supplementary Note 4 Collective motion in agents with an allocentric representation of space in the spin system model 5
Supplementary Note 4.1 Phase transitions 6

Supplementary Note 5 Order-disorder transition and cohesive motion in groups of agents with an egocentric representation of space in thee spin system model 7
Supplementary Note 5.1 Order-disorder phase transition 7
Supplementary Note 5.2 phase diagram in $\beta - h_t^s$ plane 8

Supplementary Note 6 Parameter dependence of collective motion in the spin system model 9
Supplementary Note 6.1 Dependence on individuals' network characteristics 9
Supplementary Note 6.2 Density dependence 9

Supplementary Note 7 Neural dynamics of agents during collective movement 10

Supplementary Note 8 Collective motion with short-range repulsion and long-range attraction 10

Supplementary Note 9 Distance-dependent social attraction 11

Supplementary Note 10 Neural Field Model: Individual motion and information acquisition 12
Supplementary Note 10.1 Free motion 12
Supplementary Note 10.2 Target tracking 12

Supplementary Note 11 Neural Field Model: Collective motion 14
Supplementary Note 11.1 Egocentric neural field model 14
Supplementary Note 11.2 Allocentric neural field model 14
Supplementary Note 11.3 Phase transitions 14
Supplementary Note 11.4 Parameter dependence 15

Supplementary Note 12 Switch between allocentric and egocentric representations 16

Supplementary Note 13 A modified model with constant speed with self-organized head direction adjustment **17**

Supplementary Note 14 The model with no recurrent connections **17**

Supplementary Note 15 Measures of collective movement **18**

Supplementary Note 16 The general conceptual formulation **20**

Supplementary Note 1 The spontaneous network dynamics in the spin system model

In this section, we study the network dynamics in the spin system model with cosine-shaped synaptic connectivity ($\nu = 1$) in the absence of external input, $h_0 = 0$. The internal dynamics of the ring is similar for other values of ν. The study of the correlation between adjacent spins, $\langle s_i s_{i+1} \rangle - \langle s_i \rangle^2$ as a function of β, in Supplementary Figure. 1(a) shows that for small β, the system is in the disordered phase, where spins take random states and no correlation between adjacent spins is observed. For high β, the system settles in an ordered phase where adjacent spins tend to assume the same state. As β increases, the correlation between adjacent spins increases continuously, which suggests the transition between the ordered and disordered phases is a continuous transition. The responsiveness of the system to external stimuli can be measured by the susceptibility defined as the change in the average activity of spins in response to the variation of the external field, $\chi(h = 0) = \frac{dm}{dh}|_{h=0}$. The zero field susceptibility, $\chi \equiv \chi(h = 0)$ plotted in Fig. Supplementary Figure. 1(b), shows a peak at the phase transition point. This suggests that the responsiveness of the network to external stimuli is maximized at the transition point. Furthermore, the examination of the magnetization of the system, $m = (1/N_s) \sum_i s_i$, in Fig. Supplementary Figure. 1(c) shows that at the criticality, the system has its minimum activation. In both the ordered and disordered phases, the magnetization is zero. Nevertheless, the existence of a correlation between adjacent spins indicates that a long-range order exists in the ordered phase. As will be shown in the next sections, when we allow the network to move, the agent's speed distribution shows a shift from an unimodal to a bimodal distribution at the critical point, which provides a simple way to detect the critical point.

What does the ordered state of the network look like? The Hamiltonian of the system is invariant under translation along the ring. This symmetry requires zero magnetization above the critical point. However, at criticality, this symmetry breaks, and the system exhibits long-range order. The translational invariance leads to an N-fold degeneracy of the ordered states, in each of which, spins starting from position l up to $N_s/2 + 1$, for $l = 0, .., N_s - 1$ is in the active state, and the rest in the inactive state. This leads to a bump of activity leading to a net direction of motion along $\boldsymbol{\alpha}_i$. However, for small β thermal noise drives transitions between states. This can lead to frequent shifts between bumps of activity for smaller values of β and smooth movement of the bump for larger values of β.

Supplementary Note 2 Individual motion in the spin system model

In this section, we study the motion patterns of an individual agent during free motion and in the presence of a target in hte spin system model. We begin by plotting the distribution of speed along the x-axis, $P(v_x)$, and its absolute value, $P(v)$ for different values of β close to the critical point in Supplementary Figure. 2. Here, we show results for an egocentric representation of space. The results for an allocentric representation of space are similar.

The distribution of v_x (and v_y) is a Gaussian in the disordered phase. At the critical point, the distribution shifts from an unimodal Gaussian to a bimodal distribution in the ordered phase. The distribution of speed shifts from small values to large values and broadens at criticality. This is due to the fact that in free motion (absence of external stimuli), the network dynamics is in fact decoupled from the space. Consequently, the representation of space does not affect the network dynamics. The situation, however, changes in the presence of external stimuli, which effectively, couples the network dynamics with space, through the agent's egocentric or allocentric representation of space.

Examples of the network dynamics and the resulting motion patterns for an allocentric and ego-

centric representation of space are presented in Supplementary Figure. 3 and Supplementary Figure. 4, respectively. Here, β is chosen to encompass a broad region from above but close to criticality (small β) to far below the criticality (large β in the ordered phase). For small values of β, noise drives the transition between states. This leads to the agent's random walk-like behavior. However, the resulting motion pattern depends on the representation of space. While in the presence of an allocentric representation of space, the trajectory resembles a random walk, in the presence of an egocentric representation of space the agent shows intermittent dynamics between small jumps where it spends long times exploring small regions and larger jumps. This results from the transitions between attractors corresponding to circular motion along circles with varying radii.

Examples of the network dynamics and the resulting motion patterns for an allocentric and egocentric representation of space in the presence of external stimuli are presented in Supplementary Figure. 5 and Supplementary Figure. 6, respectively. The presence of external stimuli has different effects on the network dynamics depending on the representation of species. In the absence of an internal representation of space (egocentric representation of space), external stimuli tend to stabilize a bump of activity. This can help the agent to stay stationary once it finds the target. On the other hand, in the presence of an internal representation of space, external stimuli destabilize a bump of activity. For large values of β, such external stimuli can drive damped traveling waves on the network which correspond to circular or spiral motion towards the target. Furthermore, for a larger value of β, the agent may occasionally lose interest in the target and move away.

Supplementary Note 3 Individual information acquisition in the spin system model

As argued in the main text, the information acquisition capacity of the network (the ability to reach and stay close to a target) is optimized close to (but not at) criticality. However, the agent faces a speed-accuracy trade-off: Since being in the ordered phase can increase the agent's speed, for higher target speed the information acquisition capacity of the agent is higher in the ordered phase, and the distance of the optimal region to criticality increases by increasing the target speed. In this section, we show that this phenomenology holds for other parameter values.

We begin with network size in Supplementary Figure. 7 and Supplementary Figure. 8. We present the distance of the agent from a moving target with different target speeds, indicated in each panel, for both allocentric and egocentric representations of space. For smaller network sizes, the critical region broadens. Consequently, we observe a broader optimal region. However, for larger network sizes, the optimal region approaches the critical point. Furthermore, for both network sizes, we observe that the average distance of the agent to the target is lower for an egocentric representation of space when the target speed is lower. However, an allocentric representation of space performs better for the high speed of the target, where the environment changes rapidly.

The effect of the speed constant, v_0 is investigated in Supplementary Figure. 9. Here, the average distance of an agent from a moving target with an allocentric representation of space (a) and egocentric (b) representation of space is plotted as a function of β for a small value of v_0. A smaller value of v_0 leads to slower movement of the agent and amounts to a scaling of the space size (L) and the target speed (v_t. Thus, it leads to a higher relative speed of the target with respect to the agent. Consequently, an agent with an allocentric representation of space performs better than an egocentric representation of space, due to faster (relative) environmental changes.

The effect of the receptive field is studied in Supplementary Figure. 10 and Supplementary Figure. 11, for respectively, an egocentric and an allocentric representation of space. In both cases, different values of the receptive field are shown. Here the target speed is equal to $v_t = 1$, which is small

compared to the agent's speed which can exceed 3 in the orderred phase. The receptive field does not affect the distance of the agent from the target appreciably and in all the cases, the optimal distance occurs close to criticality.

The effect of the amplitude of external stimuli is studied in Supplementary Figure. 12 and Supplementary Figure. 13, where two different values of h_0 are compared. In both cases, the optimal region happens at criticality. As intuitively expected, higher external stimuli decrease the average distance.

Finally, the effect of the neural tuning parameter is studied in Supplementary Figure. 14 and Supplementary Figure. 15, for an allocentric and an egocentric representation of space, respectively. Smaller tunning parameters amount to a higher proportion of inhibitory connections. That is, the synaptic connectivity between neurons decays faster, and turns into an inhibitory connection, by increasing their distance on the network. Thus, for smaller inhibitory connections, ordering becomes more difficult and a smaller amount of noise is enough to prevent the ordering process. Consequently, the critical point shifts to larger values for smaller values of ν. However, in all the cases the distance to the target is minimized close to the respective critical point of the network.

Supplementary Note 3.1 decision-making speed: Reaching the target.

So far we have studied the information acquisition capacity of an agent in following a moving or fixed target. Successful decision-making in such tasks requires the agent to exhibit both high decision-making speed (reaching the target) and accuracy (staying in close proximity to the target). In many contexts, the stationary long-time behavior may not be of high importance and the agent may need to make a fast decision, such as reaching a fixed target as fast as possible. While successful decision-making in such a task requires high decision-making speed, accuracy can still be of importance depending on how accurately the agent needs to accomplish the task. In Supplementary Figure. 16 we study this problem by plotting the decision time required for the agent to reach a stationary target. We vary the accuracy required for the successful accomplishment of the task by testing different vicinities of the target, d_0 that the agent should reach before the simulation stops. We compare the results for allocentric and egocentric representations of space.

The result shows that optimal decision-making speed is reached in the ordered phase. While an allocentric representation of space is beneficial when high accuracy is not needed (large d_0) when the agent needs to reach a close proximity of the fixed target, an egocentric representation can be beneficial.

Supplementary Note 4 Collective motion in agents with an allocentric representation of space in the spin system model

In this section, we study the collective motion of agents with an allocentric representation of space. In Supplementary Figure. 17 to Supplementary Figure. 19, we present the phase diagram of the system in the $\beta - h_s^t$ plane. For better visibility, in Supplementary Figure. 20 to Supplementary Figure. 22, we present the same measures as a function of total social attraction for different values of β.

At a baseline social attraction of zero, agents exhibit independent movement. An increase in h_s^t marks a critical transition to a phase characterized by ordered motion. This ordered phase is quantitatively captured by the Global Order (GO), which is taken to be the global angular order parameter (AOP, illustrated in subplot (a)) and the vectorial order parameter (VOP, depicted in subplot (b)), alongside their localized counterparts—topological VOP (shown in subplot (c), which is taken to be a measure of Local Order, LO, in the main text) and geometric VOP (presented in

subplot (d)). We note that here, we will be using a broader set of order parameters to study collective behavior compared to the main text.

These order parameters increase with rising social attraction. A further increase in h_s^t induces the coalescence of agents, culminating in a state devoid of net translational movement. In this highly cohesive phase, both the mean nearest neighbor distance (examined in subplot (e)) and the all pair distance (the average distance among all pairs, (g)), together with the global order parameters, reach their minimum, indicating a diminution in spatial dispersion due to increased social attraction.

Interestingly close to the collective motion-cohesive motion phase transition, local order is minimized for all system sizes. This is due to high fission-fusion dynamics in this regime. Beyond this phase transition, increasing the social attraction induces higher local order (but not global order and collective motion). We note that the group of packed agents shows a collective motion with a small speed compared to the speed magnitude of the individuals (which is close to π) for too high social attraction.

The temporal evolution of the system is presented in Supplementary Figure. 23 to Supplementary Figure. 28. Here, AOP (LO), VOP, the mean distance between all pairs, and mean nearest neighbor distance (in Supplementary Figure. 23, Supplementary Figure. 25, and Supplementary Figure. 27, for groups of $N = 320$, $N = 80$, and $N = 20$, respectively) and topological and geometric order parameters (in Supplementary Figure. 24, Supplementary Figure. 26, and Supplementary Figure. 28, for groups of $N = 320$, $N = 80$, and $N = 20$, respectively) as a function of time are plotted for different values of h_s^t.

In all the group sizes, for too small social attraction, low global and local order and a high distance between agents are observed. In the ordered phase, global and local orders increase and the distance between agents decreases. However, the dynamics are starkly different in large and small groups. In small groups high global order in which all the agents form a group with low distance and consistently move together is observed. On the other hand, the dynamics show intermittency for larger group sizes. While local order always remains high, global order shows intermittent transitions between high and low values. Similar intermittency is observed in the distance between agents. This intermittency results from the strong fission-fusion dynamics in large groups, according to which while at some time period all the individuals form a large group moving coherently, at other times groups are split between subgroups of different sizes. Furthermore, a comparison of groups of 80 and 320 agents shows that this intermittency is higher in larger groups.

For too high values of social attraction, the system is found in the cohesive motion phase. In this phase, local order remains high and the mean distance between agents reaches its minimum. However, global order drops, indicating the lack of collective motion.

Supplementary Note 4.1 Phase transitions

In this section, we study phase transitions in the collective dynamics of agents with an allocentric representation of space. In Supplementary Figure. 29, we present collective movement measures as a function of the total social attraction in groups of various sizes. Here, the phase transitions studied so far can be observed. Namely, as the social attraction increases, the system shows a phase transition to an ordered phase with collective motion in which both local and global order reach a high value and the distance between agents decreases. Further increasing the social attraction, the system shows a second phase transition to a cohesive phase where global order decreases, and the mean distance between agents reaches its minimum. Yet local order is preserved and increases by increasing social attraction after reaching a minimum.

Before proceeding to study the phase transitions in a large β regime, we note that for small values of β the system does not exhibit singular phase transitions. Nor does it exhibit collective motion. This

can already be seen in the contour plots presented in Supplementary Figure. 17 to Supplementary Figure. 19. To better see the behavior of the system in small β regime, in Supplementary Figure. 30, we present the collective motion order parameters as a function of the total social attraction, h_s^t, for a small value of $\beta = 20$. While this value of β corresponds to the ordered phase of the ring attractor network, it is not too far from the disordered phase. Consequently, individual movement is too noisy and random for this value of β to allow the collective to maintain long-range order. This leads to the lack of collective motion in this phase. Rather by increasing the social attraction, the system exhibits a cross-over from the disordered phase to the cohesive phase. While global order is lacking in the cohesive phase, a high local order is observed. Nevertheless, the increase in order by increasing the social attraction is gradual but not singular. This shows that as the total social attraction increases, the system shows an order-disorder cross-over, but not a singular phase transition.

The nature of the phase transitions in the large β regime is investigated in Supplementary Figure. 31 to Supplementary Figure. 38. Here we present the distribution of different order parameters in groups of, $N = 10$, $N = 20$, $N = 40$, and $N = 80$ individuals. The order parameters in groups of 160 and 320 individuals are presented in Supplementary Figure. 39 and Supplementary Figure. 40.

In Supplementary Figure. 31 and Supplementary Figure. 32, we present the distribution of AOP (taken to be the Global Order parameter in the main text) for successively larger values of total social attraction. Here we present the results for different system sizes. Similarly, Supplementary Figure. 33 and Supplementary Figure. 34 show the distribution of the topological order parameter (which is another measure of global order). The results for larger system sizes can be seen in Supplementary Figure. 39 and Supplementary Figure. 40. For too small h_t^s, the distribution shows a peak for small values, which indicates a lack of global and local order. As h_s^t increases, a phase transition to the ordered phase is observed. The phase transition appears to be discontinuous in small systems, due to the bimodality of the distribution. However, this transition becomes a continuous transition in larger system sizes. This can be seen by noting that the distribution does not show distinct peaks in large system sizes. Rather, it moves continuously from a peak at small values to a peak at large values, as h_t^s increases, and it broadens at a critical point. This phenomenology is characteristic of a continuous phase transition.

By further increasing the social attraction, a second phase transition is observed in which both local and global order decrease continuously. This is indicated by a continuous movement of the peak to smaller values. We note that, by further increasing the social attraction (not shown), local order increases after reaching a minimum. However, global order remains low.

The distribution of the mean nearest neighbor distance and mean distance between all pairs are shown in Supplementary Figure. 35 to Supplementary Figure. 38. In this case, by increasing the social attraction the distribution remains unimodal distribution and its peak continuously moves from large values in the disordered phase to a small value in the ordered phase.

Supplementary Note 5 Order-disorder transition and cohesive motion in groups of agents with an egocentric representation of space in thee spin system model

Supplementary Note 5.1 Order-disorder phase transition

In this section, we study the collective dynamics of agents with an egocentric representation of space. As depicted in Supplementary Figure. 41, an increase in the total social attraction, h_t^s, leads to an order-disorder transition. This transition, however, does not lead to collective motion. This

can be seen by noting that both the angular order parameter (AOP, taken to be GO in the main text) and the vectorial order parameter (VOP, another measure of global order) remain low across various population sizes, indicating limited collective movement. Furthermore, while low global order is observed in small groups it decreases by increasing population size (because the sum of a small number of random numbers approaches zero only for a large number). However, the system exhibits local order, manifested by the high value of topological VOP (taken to be LO in the main text) and geometric VOP (another measure of local order). This local order stems from the fact that while most of the agents are stationary, there is a significant alignment in the agents' heading directions at higher susceptibilities.

We note that the increase observed in geometric VOP for larger groups results from the fact that geometric VOP is not normalized by the number of agents and increasing the number of agents can lead to an increase in the sum of the movement speed due to higher numbers of agents. However, normalized topological VOP saturates to the maximum value of 1 as the group size increases. Besides, the mean nearest neighbor distance and mean distance between all the pairs decrease by increasing the social attraction. However, these measures show little dependence on population size, indicating the lack of density dependence. Finally, a nonzero but small angular momentum is observed in the cohesive phase. The small value of angular momentum results from the fact that most of the agents are stationary and do not show considerable movement.

In Supplementary Figure. 42 we investigate the distribution of Global Order (AOP). As can be seen, changing social attraction does not affect the distribution of GO, which peaks around a small value approaching zero for large group sizes. The discontinuous nature of the order-disorder transition is exhibited in Supplementary Figure. 43, where the distribution of LO (topological VOP) is plotted. As the social attraction increases, this distribution shows a transition from small values to large values. Furthermore, while this distribution is bimodal close to the transition for small system sizes, for larger system sizes this transition sharpens and bimodality is not observed.

Finally in Supplementary Figure. 44 and Supplementary Figure. 45 we present the distributions of the mean nearest neighbor distance and distance between all the pairs. Both distributions undergo a discontinuous transition from the disordered phase where agents show large mean distances, to the ordered phase where the agents coalesce and the mean distance between agents decreases.

Supplementary Note 5.2 phase diagram in $\beta - h_t^s$ plane

The phase diagram of collective dynamics of agents with an egocentric representation of space in the $\beta - h_t^s$ plane is presented in Supplementary Figure. 46. For better visualization, in Supplementary Figure. 47, we present the same order parameters as a function of social attraction and for different values of β. For too small social attraction, the agents move independently. As social attraction increases, collective motion is not observed. This is indicated by low values of the global angular (GO) and vectorial order parameters, both a measure of global order in the system. Rather, the system shows a phase transition to a phase where cohesive motion with low global order and low mean distance between agents, but high local order is observed. A slight increase in the global order parameter is observed for too high social attraction. This is due to the fact that while the agents coalesce for high social attraction, the group of packed agents shows a slow collective motion for too high social attraction. Normalized and total angular momentum generally remain small but nonzero due to the fact that while most of the agents remain stationary, a small fraction of agents show rotational movement in the pack of agents.

The temporal evolution of order parameters is presented in Supplementary Figure. 49 and Supplementary Figure. 48. Here, the two phases of collective dynamics indicated by high local order (topological and geometric VOP in Supplementary Figure. 48), and low distance between agents for

cohesive motion for large social attraction and low local order and high distance between agents can be observed. In both phases global order remains small.

Supplementary Note 6 Parameter dependence of collective motion in the spin system model

Supplementary Note 6.1 Dependence on individuals' network characteristics

In this section, we show that similar patterns are observed under the variation of the agents' network structure. In Supplementary Figure. 50 to Supplementary Figure. 52, we present collective movement order parameters in networks or groups of 80 individuals, each equipped with networks of $N_s = 100$ and $N_s = 200$ spins. Each figure shows different inverse temperatures. Similar phenomenology is observed in both cases. Namely, as the social attraction increases the system shows two successive phase transitions: First an order-disorder transition to a collective motion phase, and then a phase transition from collective motion to cohesive motion.

In Supplementary Figure. 53 to Supplementary Figure. 55 we present measures of collective movement in a community of 80 agents each equipped with a network with different values of the tuning parameter, ν. Each figure shows a different network inverse temperature. In this case, as well similar phenomenology is observed for different tuning parameters.

In Supplementary Figure. 56, we examine the variation of speed constant, v_0. The constant only affects the agent's speed. Similar phases are observed for different values of speed constant. We note that a smaller value of VOP results from a smaller net speed of the agents (consequently, a smaller speed of the collective). Similarly, a higher value of geometric VOP and a smaller distance between agents result from the slower speed of the agents. More precisely, the effect of agents' absolute speed is only relative. Consequently, the phenomenology of the speed with a smaller speed constant is equivalent to an increase in the dimension of the space keeping the v_0 constant.

Finally, in Supplementary Figure. 57 and Supplementary Figure. 58, we investigate the dependence of collective motion of the width of the receptive field of the neurons, σ, and base field on the network h_b. While collective movement is observed for different values, we find that smaller receptive field width increases global order in the collective movement.

Supplementary Note 6.2 Density dependence

The density dependence of collective motion is investigated in Supplementary Figure. 59. Here measures of collective movement for $N = 80$ agents in a square arena with periodic boundaries of linear dimensions, $L = 1000$ and $L = 10000$ are compared. We observe that density does not affect the phase transitions. Rather, similar phase transitions and similar values of social attraction are observed in both small and large arenas, indicating a lack of density dependence. Intuitively this results from the fact that in our model agents move towards each other. Consequently, the mean distance between agents for high social attraction is independent of the size of the arena. We note that for smaller social attractions the mean distance between agents shows dependence on the size of the arena. This is due to the presence of fission-fusion dynamics based on which the movement of the agents shows intermittency between states of high and low order.

Furthermore, the global order increases for larger system sizes. This is due to the fact that the coalescence of subgroups of coherently moving agents in the system is a source of reduction of globally coherent motion. Such collisions are more frequent in smaller system sizes and by increasing the system size, longer periods in which the whole group moves coherently are observed. On the other hand, the normalized topological order parameter does not show dependence on the system size. This

is indicative of the fact that the ordering process is density-independent. The geometric VOP on the other hand is larger in large system sizes. This is due to the higher separation of subgroups of coherently moving agents in larger arenas.

Supplementary Note 7 Neural dynamics of agents during collective movement

Supplementary Figure. 60 and Supplementary Figure. 61 illustrate the neural activity during collective movement in a population of 80 agents, in the spin system model. To increase visibility, the population is decomposed into two sets of 40 agents, presented in Supplementary Figure. 60 and Supplementary Figure. 61. The bright colors represent active neurons, while the dark colors indicate inactive neurons. The agents' trajectories are depicted in Supplementary Figure. 62, where time is color-coded.

The population of agents can be decomposed into subgroups with synchronized neural dynamics, which move together. This can be seen in Supplementary Figure. 60 and Supplementary Figure. 61 where, the neural activity of the population can be decomposed into sub-groups with similar activities. During the fission-fusion dynamics, individual agents change their group, groups split, or new groups are formed, leading to changes in the subgroups of synchronized agents.

Supplementary Figure. 63 provides a detailed view of synchronization and fission-fusion dynamics in collective movement by focusing on a subset of 10 agents. The top panels show the trajectories of 10 agents, broken into synchronized groups, within a population of 80 agents. Here the same simulation presented in Supplementary Figure. 63 is used, where, the trajectories of all the agents can be seen. The bottom panels display the neural activity of these agents. Among the 80 agents shown in Supplementary Figure. 60 and Supplementary Figure. 61, three groups with similar neural dynamics are highlighted. Notably, Agent 5 changes its group around time 1000. While Agents 1 to 7 exhibit similar neural dynamics and trajectories up to this point, their dynamics diverge afterward, resulting in the group's split into two distinct groups. Agent 5 is better synchronized with Agents 3 and 4 before this time and becomes synchronized with Agents 6 and 7 afterward.

In contrast, agents 8 to 10 show a subgroup of highly synchronized agents who remain synchronized and move together and away from others during the whole time shown in the figure.

While the synchronization of the neural dynamics is observed in all collective movement phases, the degree of synchronization changes depending on the collective motion pattern. An example of highly coherent motion across the population is presented in Supplementary Figure. 64 and Supplementary Figure. 65. The snapshot of the trajectories during this time is presented in Supplementary Figure. 66. The parameter values used here are the same as before. However, here a segment of simulation in which the whole population shown coherent motion is shown. As can be seen, in this case, most of the agents show similar and synchronized neural activity, leading to a highly coherent directed collective motion.

Supplementary Note 8 Collective motion with short-range repulsion and long-range attraction

In this section, using the spin system model, we study a variant of the model where individuals avoid collision. We implement this by defining a collision radius, such that when the distance of an agent to the focal agent is below this radius, the social attraction field becomes negative. That is, when the distance between individuals drops below r_0, where r_0 is a collision radius, individuals are coupled

with a negative external field. We take the repulsive field to be relatively large, $h_r = -0.1$. With such a value, repulsion in short distances becomes a sufficiently strong force and dominates the network dynamics, and in practice individuals turn away from each other when closer than the collision radius.

In Supplementary Figure. 67 to Supplementary Figure. 70, we study the dynamics of collective motion by examining the influence of short-range repulsion and long-range attraction among agents. Here, we present measures of collective movement in the plane defined by total social attraction (h_s^t) and collision radius.

Supplementary Figure. 67 shows contour plots of various collective motion metrics plotted against the total social attraction (h_s^t) and collision radius. For better visualization, Supplementary Figure. 68 illustrates different measures of collective motion as a function of h_s^t for various collision radii. As the social attraction increases, agents transition from independent movement to ordered motion. Specifically, a higher h_s^t triggers a phase transition leading to ordered movement. Further increasing the social attraction, a second phase transition to cohesive motion is observed. This phenomenology is similar to that observed in the model in the absence of short-range repulsion. However, an increase in the collision radius, which represents the distance below which agents avoid collisions, generally reduces both global and local order. This phenomenon is evident as GO (the angular order parameter (AOP)), vectorial order parameter (VOP), LO (topological VOP), and geometric VOP, all decrease with increasing collision radius. Additionally, mean nearest neighbor distance and all-pair distances increase, indicating a dispersion among agents due to the avoidance behavior. Similarly to the model without repulsion, too large social attraction results in cohesive motion.

The temporal evolution of collective movement metrics is depicted in Supplementary Figure. 69. This figure shows how different values of h_s^t influence the system's behavior over time. In the absence of social attraction, agents exhibit no collective motion. As h_s^t increases to moderate levels, collective motion emerges, characterized by intermittent fission-fusion dynamics. For very high h_s^t (not shown), agents display cohesive motion with low global order and minimal inter-individual distances, indicating a tightly packed but disordered state. We note that the packing of the agent decreases by increasing the collision radius, such that the agents occupy a larger area due to maintaining a larger distance from each other.

Finally, Supplementary Figure. 70 focuses on the time dependence of the normalized topological and geometric VOP for different h_s^t values. The geometric VOP, calculated for neighbors within a distance smaller than $L/100$, highlights the local ordering influenced by both long-range attraction and short-range repulsion.

Supplementary Note 9 Distance-dependent social attraction

To examine the effect of the distance dependence of social attraction on collective motion we consider a modification of the collective motion model, using the spin system model of individual decision-making, where the magnitude of the social field decays exponentially with distance, with a characteristic length, L/ζ. Thus, in this model, the magnitude of the social field acting on an agent, resulting from another agent at a distance, d, equals $h_0^s \exp(-d/(L/\zeta))$. For $d \leqslant L/\zeta$, the exponential term approaches 1 and thus, below the distance, d, the social attraction is approximately constant and does not depend on distance. For $d > L/\zeta$, on the other hand, social attraction decays exponentially fast. Thus, with this parametrization of the distance dependence of social attraction, we can examine different empirically relevant ecologies, from, effectively no distance dependence to strong distance dependence above a characteristic length, L/ζ.

The results of our analysis are presented in Supplementary Figure. 71 and Supplementary Figure. 72. In Supplementary Figure. 71, we present the contour plot of the order parameters as a function

of total social attraction, h_s^t, and ζ. The result shows that distance dependence has two main effects. First, it shifts the phase transitions to the onset of collective and cohesive motion to larger values of h_t^s. This is the case since with a stronger distance dependence, social attraction effectively decays faster with distance. Consequently, the effective social attraction reduces, and the constant term, h_0^s should be rescaled to retain a similar effective social attraction, when it decays with distance.

More importantly, while collective motion is observed for larger values of ζ, local and global order decreases with increasing ζ, such that for too large values of ζ only a small global order is observed. This shows that strong distance dependence on social attraction can reduce order. This is the case because with strong distance dependence (a smaller radius of social attraction), while subgroups of coherently moving individuals are formed, a large fraction of individuals are found in isolation and move freely from others, due to a too-weak social attraction they experience once away from the group. This fact reduces local and global order in the collective motion phase. Increasing the magnitude of social attraction can not fully compensate for its distance dependence. Rather, it can drive a phase transition to the cohesive motion phase where all the individuals coalesce in a cohesive block.

Supplementary Note 10 Neural Field Model: Individual motion and information acquisition

Supplementary Note 10.1 Free motion

The network activity and the resulting movement patterns during free motion in the neural field model are shown in Supplementary Figure. 73 and Supplementary Figure. 74, for different values of β, and for an allocentric, and an egocentric representation of space, respectively. In the neural field model, the membrane potential of neurons, u, is a continuous variable. While the potential is (in principle) unconstrained, by choosing that neural activity to be a tangent hyperbolic function of the neural potential, it is ensured that this variable does not exceed the interval between -1 and $+1$.

Starting from a random initial condition for neuron potentials, u_i, for small values of β, after a short transient period, the agent stops moving. The trajectory of the agent with an allocentric perception of space during the transient period is a directed line and with an egocentric representation of space, it is a circular trajectory with decaying radi, leading to an inward spiral. As β increases, a bump of activity appears in the network, leading to a motion along a directed trajectory, with an allocentric representation of space, and a circular trajectory, with an egocentric representation of space. Because the agent's speed increases in initial stages, with an egocentric perception of space, the increasing speed leads to an outward spiral in initial times.

Similar trajectories are observed for larger values of β. However, by increasing β, the agent's speed increases until it saturates for too large values of β.

Supplementary Note 10.2 Target tracking

The movement patterns while tracking a fixed target in the neural field model are shown in Supplementary Figure. 75 and Supplementary Figure. 76, for different values of β, and for an allocentric and an egocentric representation of space, respectively. The agent's speed in the neural field model can change by introducing targets, such that, in a small β regime, where the agent does not move in the absence of a target, it starts to move once targets are introduced (see $\beta = 2.55$). With both an allocentric and egocentric representation of space, a variety of patterns are observed on the ring attractors in the presence of external stimuli. With an allocentric representation of space, the agent moves towards the target for sufficiently high values of β. For smaller values of β, once the agent reaches the target, it stays close to the target. For larger values of β, the agent moves in a circular

trajectory when reaching the target. This leads to traveling waves of activity on the ring attractor network.

With an egocentric representation of space, for small values of β, the agent moves towards the target with low speed but in a directed trajectory. Besides, when reaching the target, the agent remains close to the target. As β increases, however, the agents start to move slowly away from the target in a variety of coil-shaped trajectories, once they reach the target. These movement patterns can be seen in Supplementary Figure. 77, where the trajectory of the agents in the last stages of the movement, when close to the target is shown. These simulations are the same simulations shown in Supplementary Figure. 76, shortened to the last time stages here for better visibility. We note that the behavior of the agent also depends on the strength of the external stimuli, such that for stronger stimuli the agent remains close to the target once they reach the target. Finally, while for larger values of β the agent can still reach the target in a meandering trajectory (Supplementary Figure. 76 $\beta = 5$), for too high values of β, with an egocentric perception of space, the agent often is unable to reach the target.

The average distance of the agent to a fixed and moving target is presented in Supplementary Figure. 78 and Supplementary Figure. 79, respectively. Here, the average distance of the agent from the target for both allocentric and egocentric representations of space is plotted. The simulations are run for 15000 timesteps, and the average distance of the agent in the last 5000 timesteps is calculated (over a sample of 10 simulations). Large error bars for an egocentric representation of space result from the fact that with an egocentric representation of space, the agent's trajectories are circles with varying radii, including infinite radii, corresponding to a directed motion. While in the spin system model, noise drives transitions between such movement trajectories, a bump of activity is more stable in the neural field model. This leads to the fact that switching between different trajectories is less frequent in the neural field model and in many simulations, once the agent starts to move along a circular trajectory, it can take a too long time before it switches to another trajectory, especially for large values of β and small h_t. Consequently, for large values of β an egocentric agent performs poorly in finding both a fixed and tracking a moving object. By contrast, an allocentric agent can find and track targets even for large values of β. In both cases, however, the agent's average distance from the target is minimized for medium values of β. For too small values of β, on the other hand, the agent does not move or moves too slowly toward the target. Furthermore, the optimal value of β shifts to larger values, and the strength of the external stimuli, h_t increases.

We note that, in contrast to the spin system model, in the neural field model, the neural dynamics do not exhibit an order-disorder phase transition as β increases. Rather, by increasing β, the system shows a cross-over from a state where all the neuron potentials are zero, to a state where a neural bump appears in the system. Consequently, the movement trajectories for small β in the two models are different. While in the spin system model, the agent exhibits a random-walk-like behavior for small values of β, in the neural field model the agent moves along a directed line or circular trajectory depending on its perception of space. Nevertheless, transitions between different bumps of activity, leading to transitions between different trajectories are easier for small values of β. This leads to some similarities in the phenomenology of the neural field model and the spin system model when tracking an object. Namely, the information acquisition capacity of the agent is maximized for a medium value of β, where transitions between bumps of activity can endow the agent with flexible decision-making.

Supplementary Note 11 Neural Field Model: Collective motion

Supplementary Note 11.1 Egocentric neural field model

Collective behavior in a community of 80 agents with an egocentric representation of space in the neural field model is investigated in Supplementary Figure. 80. Here, the contour plots of collective motion metrics in $\beta - h_t^s$ plane are shown. For zero or too small social attraction, the agents move independently. As social attraction increases, collective motion is not observed. Rather, the collective forms an aggregation with low global and local order, and low or relatively low mean distance between agents. We note that the behavior of the system can also depend on β. For small β, agents coalesce in the aggregation phase, resulting in a small mean distance between agents. However, for too large values of β, agents keep a distance in the aggregation phase, or they are decomposed into subgroups with slow movement. This typically results from small movements of the agents toward each other, or even slowly moving away from each other, when they are too close.

The behavior of the system as a function of time for a small value of β is shown in Supplementary Figure. 81. Here, collective motion metrics for four different values of social attraction are shown. In all the cases, global and local orders remain small. However, the mean distance between agents decreases in the initial stages of the simulations, indicating that agents, starting from random positions, move towards each other. A pick in the local order from initial time results from the transient local alignment induced by agents moving toward each other.

Supplementary Note 11.2 Allocentric neural field model

Different measures of the collective motion in the neural field model with an allocentric representation of space are shown in Supplementary Figure. 82 and Supplementary Figure. 83. As the social attraction increases, the collective first shows a phase transition from a disordered phase to a collective motion phase with high global and local order. Further increasing the social attraction drives a second phase transition from the collective motion phase to an aggregation phase with high local order but small global order and mean distance between the agents. Example time series of the measures of collective movement is presented in Supplementary Figure. 83. Here examples of the collective motion in the disordered phase and the collective motion phase, both with highly ordered directed motion, intermittency between motion patterns, and in the aggregation phase are shown.

We note that, in this model, in the disordered phase the global order parameters remain constant. The reason is that, in contrast to the spin system model, in this model, in the absence of social attraction (or target more generally) variation in the agents' speed is not observed. Rather agents always move in the same direction and with a constant (β-dependent) speed. Consequently, while measures such as local order parameters or distance measures show random fluctuations due to the changing positions of agents, global order parameters take a small, constant, and time-independent value.

Supplementary Note 11.3 Phase transitions

The order-disorder phase transition in the neural field model and for different population size is investigated in Supplementary Figure. 84 and Supplementary Figure. 85. Here the population size is, $g = 20$ and $g = 80$, respectively. The left panels show the distribution of Global Order (angular order parameter) and local order (topological order parameter) with an allocentric representation of space. The right panels show example time series of the same variables as a function of time. In all the cases, as the social attraction increases, the population shows a continuous phase transition from

the disordered state with low local and global order to a collective motion phase with high local and global order.

The collective motion-aggregation phase transition in a small ($g = 20$) and a large ($g = 80$) population size is investigated in Supplementary Figure. 86 and Supplementary Figure. 87. As the social attraction increases, the population shows a phase transition from the collective motion phase with high global and local order, to the aggregation phase with high local and low global order. Similarly to the spin system model, the phase transition seems discontinuous in smaller groups and tends to a continuous phase transition in larger groups. The apparent discontinuity of the phase transition in small groups origniate from the transition of aggregated groups of individuals between stationary and mobile groups in smaller population size.

Supplementary Note 11.4 Parameter dependence

The dependence of collective motion patterns in the neural field model on parameter values is investigated in Supplementary Figure. 88 to Supplementary Figure. 95. In each of the simulations, using the base parameter values, $N_s = 100$, $\sigma = 0.1$, $\nu = 0.5$, $v_0 = 0.05$, $\beta = 1000$, and $dt = 0.3$, $g = 80$, and $L = 1000$, we change one of the parameters to systematically investigate the effect of all parameter values. Using this analysis, we show the robustness of our results regarding the emergence of collective motion.

We begin by studying the dependence on the population size in Supplementary Figure. 88, by presenting different measures of collective motion as a function of total social attraction, for three different population sizes, g. Similar phenomenology, namely an order-disorder transition, and then, a collective motion-aggregation phase transition is observed in different population sizes. In the aggregation phase, the mean distance between neighbors decreases. While local order monotonically increases by social attraction, global order is low for too high social attraction (aggregation phase). While the phenomenology of the neural field model is generally similar to the spin system model, a notable difference is milling patterns are observed in the neural field model, resulting in higher normalized angular momentum.

The investigation of the dependence of collective motion patterns on the arena size in Supplementary Figure. 89 reveals that increasing density (decreasing the arena size) can slightly increase order due to less frequent encounters and mixing of the coherently moving groups. However, similar phases and phase transitions are observed for different densities.

The dependence of the motion patterns on global inhibition is investigated in Fig. Supplementary Figure. 90. While collective motion is observed for different global inhibitions, a larger value of global inhibition increases global order, and mean distance between agents, and decreases normalized angular momentum, indicating agents show weaker milling patterns.

The dependence of the motion patterns on the width of the neurons' receptive field, σ, is investigated in Fig. Supplementary Figure. 91. Global order and normalized angular momentum remain largely similar for the two values of σ considered. However, a larger receptive field decreases local order (alignment) by increasing the mean distance between agents. While similar phases and phase transitions are observed for different values of the neural receptive field width, for larger values of the receptive field width, collective motion phase transition shifts to smaller values of social attraction. Besides, a higher order is observed in the collective motion phase.

The dependence of the motion patterns on the tuning parameter of the synaptic connectivity of the network, ν, is investigated in Fig. Supplementary Figure. 92. The notable effect of ν is in the aggregation phase, where higher order and larger errorbars are observed. This phenomenology indicates that with larger values of ν, in the aggregation phase, the collective is more likely to form a moving aggregate, instead of a stationary one.

The dependence of the motion patterns on the speed constant, v_0, is investigated in Fig. Supplementary Figure. 93. Lower global and local order and a higher distance between the agents are observed for larger v_0. This is due to the fact that a higher speed of the agents can lead to more dramatic changes in agents' relative positions, and thus, vectorial representation of the agents for each other, and decreases the stability of highly ordered motion patterns.

The dependence of the collective motion on the integration constant dt is investigated in Supplementary Figure. 94, where it is shown that similar phases and phase transitions are observed for different values of dt. We note that a smaller VOP is observed for smaller dt. This is due to the fact that the agent's speed is proportional to dt. Thus, the absolute speed (but not alignment which is better captured by AOP) decreases with decreasing dt.

Finally, the dependence on the number of neurons is investigated in Supplementary Figure. 95. While local order and mean nearest neighbor distance remain the same for different values of N_s, VOP can increase, while AOP decreases. This is due to the fact that a higher number of neurons increases the agent's speed, which in turn can decrease local order.

Supplementary Note 12 Switch between allocentric and egocentric representations

We investigate the dynamics of the system when agents switch between an allocentric and an egocentric representation of space in Supplementary Figure. 96 to Supplementary Figure. 100. In this modification of the model, at each timestep, each agent can be found in an allocentric or egocentric state. Besides, as explained in the main text, here, we consider continuous switches between representations, where the agent's origin of the reference frame does not undergo changes when agents switch, ensuring that the agent does not get lost (the same bump of activity does not code for a new motion direction) after a switch.

In Supplementary Figure. 96 and Supplementary Figure. 98 we show the collective motion metrics as a function of the probability of switch, ω and h_t^s. We observe two regions with high global order, one for a small probability of egocentric representation (ω) and large h_t^s. This is close to the aggregation phase, where switching ensures the collective, instead of becoming a stationary aggregation, form moving aggregations.

The second region with high global and local order occurs for large ω and small h_t^s. This region is in the collective motion phase, where agents do not form highly packed aggregates. Rather, while keeping a distance from each other, move in high alignment. Examples of time series of the collective motion metric for relative high values of ω, and different values of h_t^s, can be observed in Supplementary Figure. 97.

To investigate the behavior of the system for smaller population sizes, in Supplementary Figure. 98, we present measures of collective motion in groups of 20 individuals. In smaller groups, the advantage of switching for achieving a high global order is lower. For large h_t^s, where the population exhibits moving aggregates, high global and local order is observed for purely allocentric representation of space and when the probability of being in the egocentric state is smaller than, approximately, 0.5. For larger values of ω, switching between allocentric and egocentric representations of space can only decrease global and local alignment.

For smaller values of h_t^s and in smaller groups, while switching between representations preserves collective motion, we do not observe the stabilizing effect of switching that is observed for larger groups.

To shed further light on the mechanisms using which switching between an allocentric and egocentric representation of space increases alignment, in Supplementary Figure. 99 and Supplementary

Figure. 100 we present global and local order resulting from both a time and an ensemble average (an average over 5 simulations), in groups of 80 and 20 individuals. We observe that, in a single run (bottom panels), occasionally high order, indicating highly ordered directed motion can be observed. However, results can be different in independent runs (or over time). These phenomena result from the multistability of the collective motion patterns associated with its complexity, such that the collective exhibits transitions between different motion patterns. However, switching between allocentric and egocentric representation reduces the complexity of collective motion by making different motion patterns unstable, leading to a higher probability of just one mode, highly ordered collective motion, in which individuals move together without significant turning or changing direction. Consequently, the geometrical representation of conspecifics remains relatively stable over time. This leads to high ensemble and time average global and local order when the individual switches between representations.

Supplementary Note 13 A modified model with constant speed with self-organized head direction adjustment

The collective motion model with constant speed is investigated in Supplementary Figure. 101 and Supplementary Figure. 102. In this model, we have made a small modification to the neural field model by taking the agents to always move with constant speed. However, their head direction is determined by the ring attractor network. Under our assumption that the agents always move toward the direction that they are facing (head direction), this determines the direction of the agents' movement. Here, we have considered an allocentric representation of space.

Similar phases and phase transitions that we observed in other models are observed in this model. Namely, as the social attraction increases, the collective first shows a phase transition from a disordered phase to a collective motion phase with high global and local order. Further increasing the social attraction drives a second phase transition from the collective motion phase to an aggregation phase with high local order but small global order and mean distance between the agents.

Example time series of the measures of collective movement are presented in Supplementary Figure. 102. Here, examples of the collective motion in the collective motion phase, with highly ordered directed motion, intermittency between motion patterns, and in the aggregation phase are shown.

Supplementary Note 14 The model with no recurrent connections

To examine whether recurrent connections are essential for collective motion and how they affect collective motion patterns, in Supplementary Figure. 103 and Supplementary Figure. 104 we present measures of collective movement in a community of 80 agents in the neural field model with no recurrent connections. Here collective motion order parameters are presented as a function of β and h_t^s. For too large β the individuals do not move or move too slowly. Individuals' speed increases for smaller values of β. Consequently, for large values, the simulation does not reach a stationary state in any practical time and the distance between agents remains large. For smaller values of β on the other hand, individuals move towards each other in an accelarating fashion and collapse into a dense aggregate with a random walk-like, but very slow, motion. In this state global order remains small for both an allocentric and egocentric representation, but local order reaches a relatively high value for an allocentric representation of state.

However, the motion observed in the absence of recurrent connections lacks the complexity or realism observed in the model with recurrent connections. This can be seen in a small vectorial order parameter, which in addition to the alignment of the agents' heading direction, is a measure of the

group net speed. The small value of the time average of this quantity shows the slow, random-walk-like behavior of the collective in this model.

To better see this point, in Supplementary Figure. 105 and Supplementary Figure. 106, we present measures of collective movement as a function of time. The acceleratingly decreasing distance between agents with time shows the collapse of the collective into a dense aggregate. Once the aggregate is formed, all the measures of global and local order show high fluctuations due to the slow, random-walk-like behavior of the agents. See Supplementary Vidoe 17 for an example of the dynamics of this model.

We note that, in the absence of recurrent connection, the agent does not move in the absence of targets due to the fact that external stimuli on the ring are essential to give rise to nonzero neural activity. Similarly for too strong external stimuli, the absence of inhibitory connections leads to maximal activity of all the neurons. Consequently, the net speed of the agent becomes zero and the agent does not move (because induced motion by different neurons cancels each other). Thus, in this null model, there is only a small region of the external input (total social attraction) in which movement is observed. Only this region is shown in Supplementary Figure. 103 and Supplementary Figure. 104.

Supplementary Note 15 Measures of collective movement

In this section, we define the measures used to analyze collective movement in our study. We begin by defining our noration. For each agent $i = 1, \ldots, N$ at time t, denote its position by

$$\mathbf{r}_i(t) = \big(x_i(t),\, y_i(t)\big),$$

and its velocity by

$$\mathbf{v}_i(t) = \big(v_{x,i}(t),\, v_{y,i}(t)\big),$$

with speed $\|\mathbf{v}_i(t)\| = \sqrt{v_{x,i}(t)^2 + v_{y,i}(t)^2}$. We define the unit-velocity

$$\hat{\mathbf{v}}_i(t) = \begin{cases} \dfrac{\mathbf{v}_i(t)}{\|\mathbf{v}_i(t)\|}, & \|\mathbf{v}_i(t)\| > 0, \\ \mathbf{0}, & \|\mathbf{v}_i(t)\| = 0. \end{cases}$$

Angular Order Parameter (AOP) This is calculated as the sum of the individuals' normalized velocity vectors. To do so, we have normalized direction vectors to 1 and then summed over all individuals' normalized vectors. Values close to 1 indicate strong alignment, and values close to 0 indicate weak alignment. Mathematically, the global angular order parameter at time t is defined as:

$$AOP(t) = \left\| \frac{1}{N} \sum_{i=1}^{N} \hat{\mathbf{v}}_i(t) \right\| = \sqrt{\Big(\tfrac{1}{N} \sum_i \tfrac{v_{x,i}(t)}{\|\mathbf{v}_i(t)\|}\Big)^2 + \Big(\tfrac{1}{N} \sum_i \tfrac{v_{y,i}(t)}{\|\mathbf{v}_i(t)\|}\Big)^2}\,. \quad (1)$$

We often report the time average and ensemble average (average over independent runs) of this quantity. We note that the minimum of this quantity approaches zero only for large groups. In the main text, we used AOP as a measure of global order (GO).

Vectorial Order Parameter (VOP) This is a measure of the average direction of the velocity vectors of individuals within the entire group. It is calculated as the sum of the velocity vectors of

the agents. A high value indicates strong alignment (coordinated movement in a common direction), while a low value indicates weak alignment. More mathematically, global VOP is defined as:

$$VOP(t) = \Big\| \frac{1}{N} \sum_{i=1}^{N} \mathbf{v}_i(t) \Big\| = \sqrt{\Big(\tfrac{1}{N} \sum_i v_{x,i}(t) \Big)^2 + \Big(\tfrac{1}{N} \sum_i v_{y,i}(t) \Big)^2}\,. \tag{2}$$

Topological and geometric VOP. These measures are calculated in a local neighborhood and serve as measures of local order. In the case of topological VOP, k nearest neighbors of the focal individual are considered, and in the case of Geometric VOP, all the individuals closer than a distance R are considered. Precisely, for a fixed radius R, define the geometric neighborhood of agent i at time t by

$$\mathcal{N}_i^{(R)}(t) = \{\, j : \|\Delta \mathbf{r}_{ij}(t)\| \leq R \}\,.$$

Then

$$\mathrm{VOP}_{\mathrm{geo}}(t) = \frac{1}{N} \sum_{i=1}^{N} \Big\| \sum_{j \in \mathcal{N}_i^{(R)}(t)} \hat{\mathbf{v}}_j(t) \Big\|. \tag{3}$$

For topological VOP, considering a fixed k, let $\mathcal{N}_i^{(k)}(t)$ be the set of the $k+1$ nearest agents (including i) to agent i. Then

$$\mathrm{VOP}_{\mathrm{topo}}(t) = \frac{1}{N} \sum_{i=1}^{N} \Big\| \sum_{j \in \mathcal{N}_i^{(k)}(t)} \hat{\mathbf{v}}_j(t) \Big\|. \tag{4}$$

These measures do not take values between 0 and 1, rather can take values up to the total number of individuals in a neighborhood. To achieve a standard order parameter limited to the interval $[0, 1]$, we have divided the topological VOP by $k + 1$. In all the plots we have taken $k = 5$ and $R = L/100$. In the main text, we used topological VOP as a measure of local order (LO).

We note that geometric (and similarly topological) AOP can be defined as:

$$\mathrm{AOP}_{\mathrm{geo}}(t) = \sqrt{\Big(\tfrac{1}{N} \sum_i \sum_{j \in \mathcal{N}_i^{(R)}(t)} \tfrac{v_{x,j}(t)}{\|\mathbf{v}_j(t)\|} \Big)^2 + \Big(\tfrac{1}{N} \sum_i \sum_{j \in \mathcal{N}_i^{(R)}(t)} \tfrac{v_{y,j}(t)}{\|\mathbf{v}_j(t)\|} \Big)^2}\,. \tag{5}$$

This is different from geometric VOP in that it sums over all the individuals' local neighborhoods and then calculates the norm. Whereas in the case of geometric VOP average is taken over the norm of local VOPs. However, due to the fact that the results are similar to geometric VOP, we have focused on (geometric and topological) VOP as measures of local order.

Mean distance between all pairs. This is the average distance between all the pairs in the group, $\sum_{i,j} d_{i,j}/(N(N-1))$, where $d_{i,j}$ is the distance between individuals i and j, and N is the number of agents in the group.

Mean nearest-neighbor distance The mean nearest-neighbor distance is calculated as the average nearest-neighbor distance of all the individuals.

$$D^{\mathrm{nn}}(t) = \frac{1}{N} \sum_{i=1}^{N} \min_{j \neq i} d_{ij}(t). \tag{6}$$

Angular Momentum Metrics Total angular momentum is defined as the angular momentum of the whole group around its center of mass. Let the center of mass be

$$\mathbf{r}_{\mathrm{CM}}(t) = \frac{1}{N}\sum_{i=1}^{N}\mathbf{r}_i(t),$$

and let $\Delta\mathbf{r}_i^{\mathrm{CM}}(t)$ denote the distance vector of individual i from the center of mass. Then total angular momentum is:

$$L_{\mathrm{tot}}(t) = \sum_{i=1}^{N}\Delta\mathbf{r}_i^{\mathrm{CM}}(t)\times\mathbf{v}_i^{\mathrm{CM}}(t) = \sum_{i=1}^{N}\big(\Delta x_i^{\mathrm{CM}}(t)\,v_{y,i}(t)\ -\ \Delta y_i^{\mathrm{CM}}(t)\,v_{x,i}(t)\big). \tag{8}$$

Normalized angular momentum is defined as:

$$\widehat{L}(t) = \frac{|\,L_{\mathrm{tot}}(t)|}{\sum_{i=1}^{N}\|\Delta\mathbf{r}_i^{\mathrm{CM}}(t)\|\,\|\mathbf{v}_i(t)\|}\,. \tag{9}$$

Supplementary Note 16 The general conceptual formulation

In this section, we discuss how, conceptually, it is possible to extend our models to a general multilayer network. We base our general framework on Amari's neural field model. In our general framework, the agents' decision-making is governed by a multilayer neural network, which receives sensory input from the outside world, performs neural computation, and governs motor decisions. Following Amari's neural field model [86], we consider a multilayer neural network with m layers. While the model can straightforwardly be extended to three space dimensions, here we are interested in modeling animal movement in two space dimensions, aligning most past work and the fact that collective movement in many, if not most, species effectively takes place in two dimensions [5]. As we discussed in the main text, a one-dimensional neural network with a ring structure suffices to model animal decisions in two space dimensions. Thus, without loss of generality, for conceptual simplicity, and motivated by empirical (ring) attractor networks [44, 54–59], we can think of the networks to be one-dimensional networks with periodic boundary conditions. Neurons on each layer are indexed by a continuous variable $x \in [0, 2\pi)$, where x indicates the position of the neuron on the "ring". We represent the average membrane potential of each neuron by a function $u^a(x)$. Here, $a \in \{1, .., m\}$, refers to the layer. The "firing rate" of the neuron can be represented by $F^a(u^a(x))$, where F^a is a (monotonically non-decreasing saturating) output function. The output function in general depends on the layer.

The intensity of connections between neuron x and y in layers, a and b, respectively, can be represented by the "synaptic connectivity" $J^{ab}(x, y; t-t')$. In general, $J^{ab}(x, y; t-t')$ can also depend on time lag. Such a dependence allows us to take pulse conduction or synaptic delay into account [86]. While incorporating such a memory-based representation of neural dynamics may provide interesting insights, here we do not investigate the consequences of synaptic delay. The average, background stimulation level at level a is I_0^a, which usually is taken to be non-positive [86] (we employ this by taking I_0^a to be nonnegative and adding a minus sign in the following equation). In addition, each layer is subject to a neuron-specific stimulation $I^a(x)$. The dynamics of the network are governed by the following integrodifferential equation, which guarantees that the neuron potential converges to its stimulus level in the stationary state [86]:

$$\frac{\partial u^a(x,t)}{\partial t}\ =\ -\,u^a(x,t)\ +\ \sum_{b=1}^{m}\frac{1}{2\pi}\int_0^{2\pi} J^{ab}(x,x',t-t')F^b\big(u^b(x';t')\big)dx'dt'\ -\ I_0^a\ +\ I^a(x,t),$$

(Supplementary Equation.1)

The above description closely follows Amari's neural field model [86]. The key element we add to this formulation is equipping this network with sensory input and decision output. The sensory layer, which can be thought of as the first layer, receives sensory input from the outside world. To implement this, we assume each neuron at position x, in the sensory layer, is subject to external current originating from external stimuli. We assume each neuron x in the sensory layer is equipped with a Gaussian receptive field centered on an angle $\boldsymbol{\alpha}(x)$. In two-dimensional physical space, the angle $\boldsymbol{\alpha}(x)$ can be represented by a scaler in a polar coordinate, which can be taken to be the same as x, $\boldsymbol{\alpha}(x) = \alpha(x) = x$. That is a neuron at position x along the ring encodes for stimulus arriving at an angle x with respect to the zero (origin) of the polar coordinate (the coordinate, i.e., the agent's *reference frame* can be allocentric or egocentric and will be specified shortly). Thus, we have for the receptive field:

$$I^1(x) = \frac{h_0}{\sqrt{2\pi\sigma^2}} \exp[-\frac{(\alpha(x) - \theta_{target})^2}{2\sigma^2}]. \qquad \text{(Supplementary Equation.2)}$$

Here, θ_{target} represents the angular direction of the external stimuli. The key question is to define the coordinate using which the external stimuli (i.e., the outside world) are represented in our simplified "brain". Evidence suggests that animals can represent objects in both allocentric and egocentric ways, likely, by converting egocentric representations to allocentric ones [41,48]. As mentioned before, we do not aim to model the way animals form this capacity. Rather, we aim to investigate their consequences using a general model applicable to diverse species across the levels of the organization. In the allocentric scenario, bearings to objects are represented in a world-centric (polar) frame of reference. That is, a neuron at position x along the ring, encodes for a bearing $\alpha(x)$ (e.g., $= x$) in an absolute reference frame, independent of the animal's body axis and pose, e.g., head or heading direction, but attached to the animal (so that bearings always originate from the animal's position. Thus, in this scenario, the agent has an allocentric representation of space and the network receives allocentric input about the bearing of the objects. By contrast, in the egocentric scenario, the agent lacks an allocentric representation of space and the direction $\boldsymbol{\alpha}$ is measured with respect to an egocentric, body-centered coordinate. The zero of this coordinate (which neuron is to be indexed as neuron 1) does not matter, but most naturally it can be taken to be the agent's heading direction. While we do not need to specify the agent's head direction, we can think of the animal's head direction to be dynamically updated at each movement decision to align with its heading direction. Biologically, this intuition is supported by a wealth of evidence according to which, both invertebrate [41, 54, 55] and vertebrates [68, 69] turn their head towards the direction that they are moving, such that head direction and heading directions are strongly correlated [41, 54, 55, 55, 68, 69]. Methodologically, this assumption originates from the parsimony of our model, based on which, we do not explicitly model the mechanisms (e.g., path integration) governing the agent's head direction (or body pose).

Finally, the network's output is the agents's goal direction, and thus its movement decision. This can be implemented by thinking of the last layer of the network to provide behavioral output, i.e., makes movement decisions. This is implemented by taking each neuron in the last layer to code for movement based on the agent's allocentric or egocentric refrence frame. That is each neuron α codes for movement along the allocentric or egocentric direction α^m. Similarly to before, in the allocentric case, α^m refers to an angle measured in an allocentric coordinate independent of the animal's body coordinate and movement direction. In the egocentric case, this refers to an angle with respect to the animal's head, which is taken to be the same as its heading direction. The agent's speed vector, $\mathbf{v}$ is determined based on the collective activity of its motor neurons, according to:

$$\mathbf{v} = \frac{v_0}{2\pi} \int_0^{2p} \max{(0, F^m(u^m(x))} \boldsymbol{\alpha}(x) dx. \qquad \text{(Supplementary Equation.3)}$$

The above description provides a general conceptual description of our neural field model. By defining synaptic connectivities, J^{ab}, between layers, $a \neq b$, and within layers, $a = b$, (and the external currents, $I^a(x)$ and I_0^a on each layer), one can achieve a variety of models, which can be thought of as different sensory-motor transformation or perceptual neural networks. To achieve, arguably, the most parsimonious model, which is our *neural field mode*l in the main text, we take $m = 1$ (limiting ourselves to a one-layer network). In this simplification of our conceptual framework, the preferred movement direction of each neuron is taken to be the same as the direction from which the neuron receives sensory input, $\boldsymbol{\alpha}$. Besides, to simulate the network dynamics, we take the network to be composed of N_s neurons. This leads to a discretization of the continuous network, where, the continuous variable x is replaced by discrete index i referring to neuron's position along the ring. Furthermore, motivated by empirical data, we take the synaptic connectivity of the network to be a generalized cosine shae neural connectivity, giving the network a ring structure. See the Methods in the main text for a detailed exposition.

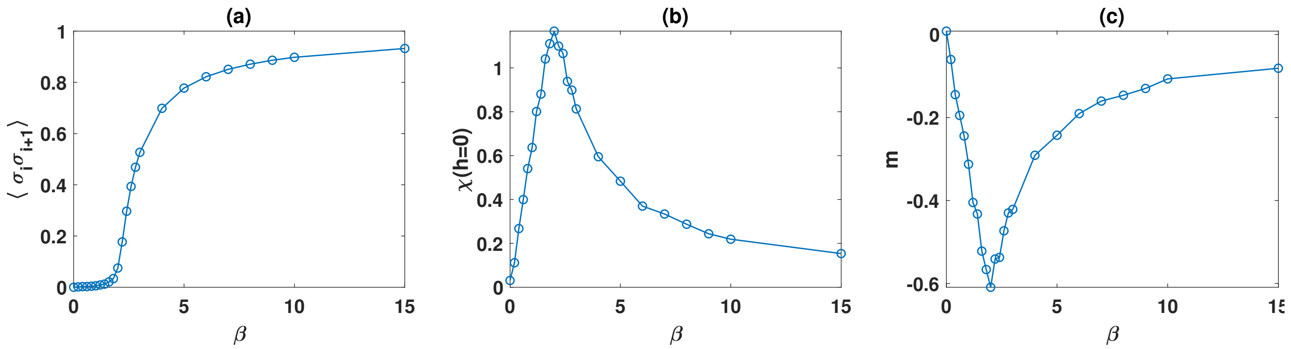

Supplementary Figure. 1: The order-disorder transition in a ring attractor network in the absence of an external stimulus. (a) The correlation function between adjacent neurons, $\langle \sigma_i \sigma_j \rangle$ is plotted. The correlation between adjacent spins is zero for small β and approaches 1 for large β. (b): The zero field suceptibility, $\chi(h=0) = \frac{dm}{dh}|_{h=0}$ is maximized at the critical point. The network activity measured by the average magnetization, $m = \Sigma_i \sigma_i$, is minimized at the critical point. Parameter values: $h = 0$, $\sigma = 2\pi/N$, $N = 400$. Plots show an average of over 500 simulations each for 8×10^6 spin updates according to Glaubar dynamics.

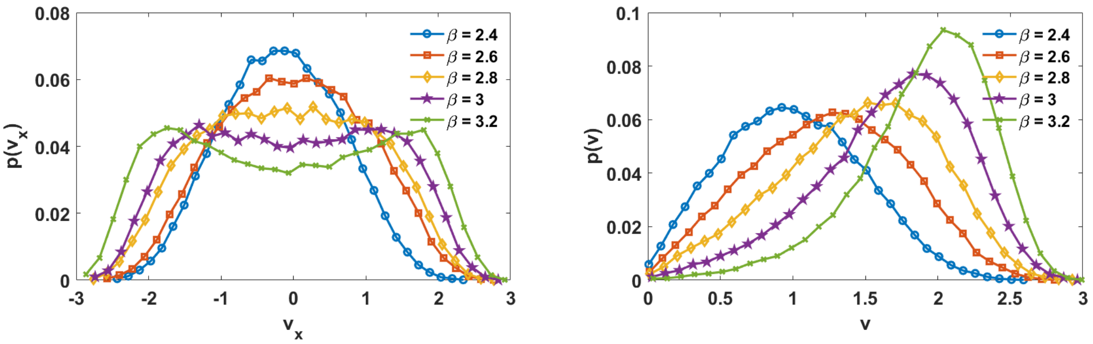

Supplementary Figure. 2: The distribution of speed along the x-axis, $P(v_x)$ and its absolute value, $P(v)$ for different values of β for free movement of the agent close to the phase transition for an egocentric representation of space. At the critical point, the distribution of v_x shifts from an unimodal Gaussian above to a bimodal distribution below the critical point. The distribution of speed shifts from small values to large values and broadens at criticality. Parameter values: $N_s = 100$, $\sigma = 2\pi/N_s$, $v_0 = 10$. The agent moves in a periodic space with linear size, $L = 1000$.

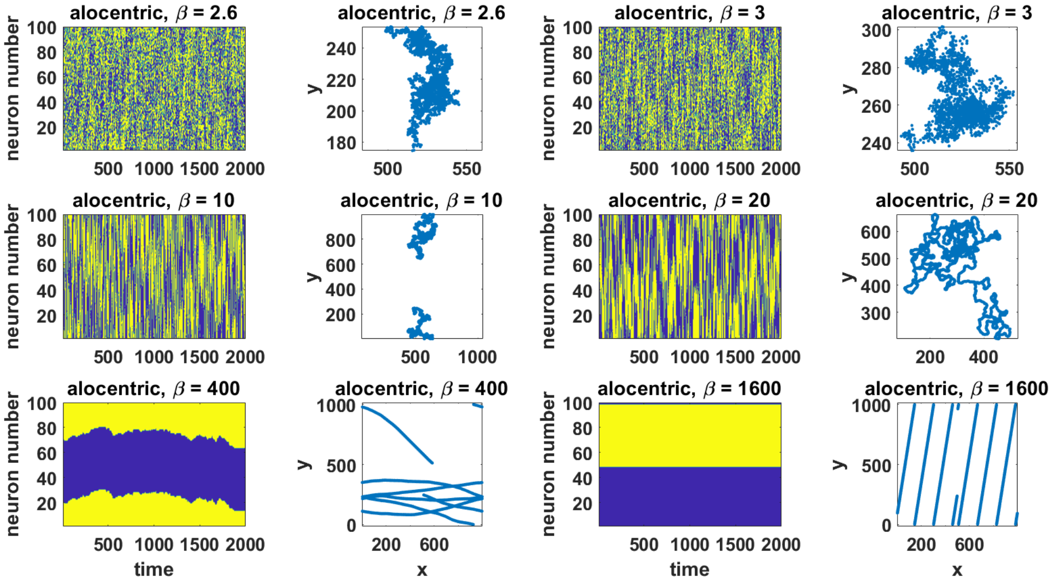

Supplementary Figure. 3: Movement patterns with an allocentric representation of space and during free motion. The network activity as a function of time (left) and the resulting trajectories (right) for different values of β, indicated on each panel are shown. The agent possesses an allocentric representation of space. For smaller values of β a more disordered phase leading to frequent transitions between states resulting in a random walk-like motion is observed and for larger values of β, an ordered phase where adjacent spins take similar values (a bump of activity) is observed. This leads to motion along a straight line with constant speed. Parameter values: $N_s = 100$, $\sigma = 2\pi/N_s$, $v_0 = 10$. The agent moves in a periodic space with linear size, $L = 1000$.

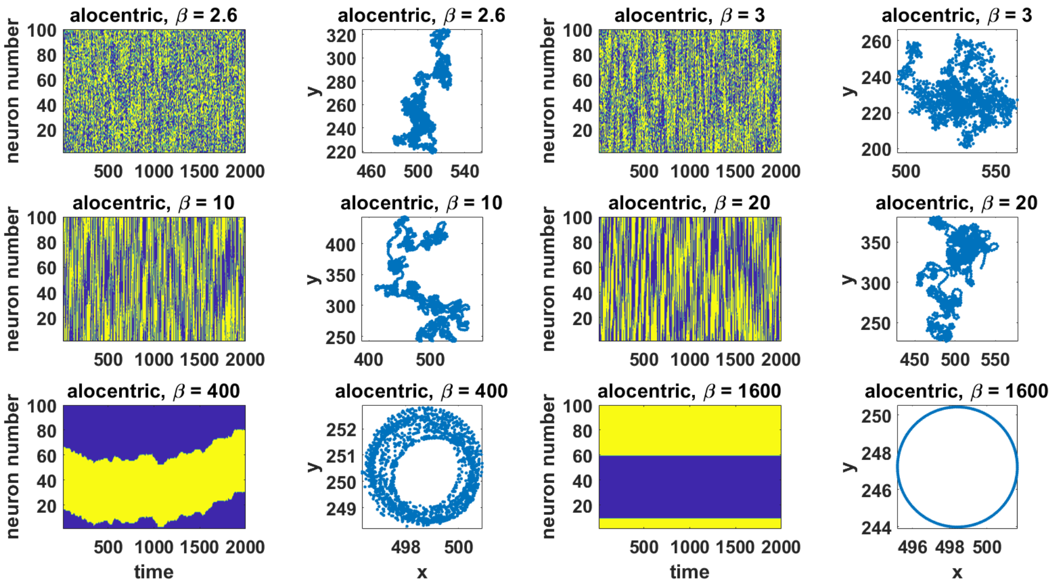

Supplementary Figure. 4: Movement patterns with an egocentric representation of space and during free motion. The network activity as a function of time (left) and the resulting trajectories (right) for different values of β, indicated on each panel are shown. The agent has an egocentric representation of space. For smaller values of β a more disordered phase leading to frequent transitions between states resulting in a random walk-like motion is observed and for larger values of β, an ordered phase where adjacent spins take similar values (a bump of activity) is observed. A bump of activity leads to a circular motion. However, for smaller values of β, frequent jumps between states, lead to a Levy flight-like behavior where the agent spends long times in small regions with intermittent longer jumps. Parameter values: $N_s = 100$, $\sigma = 2\pi/N_s$, $v_0 = 10$. The agent moves in a periodic space with linear size, $L = 1000$.

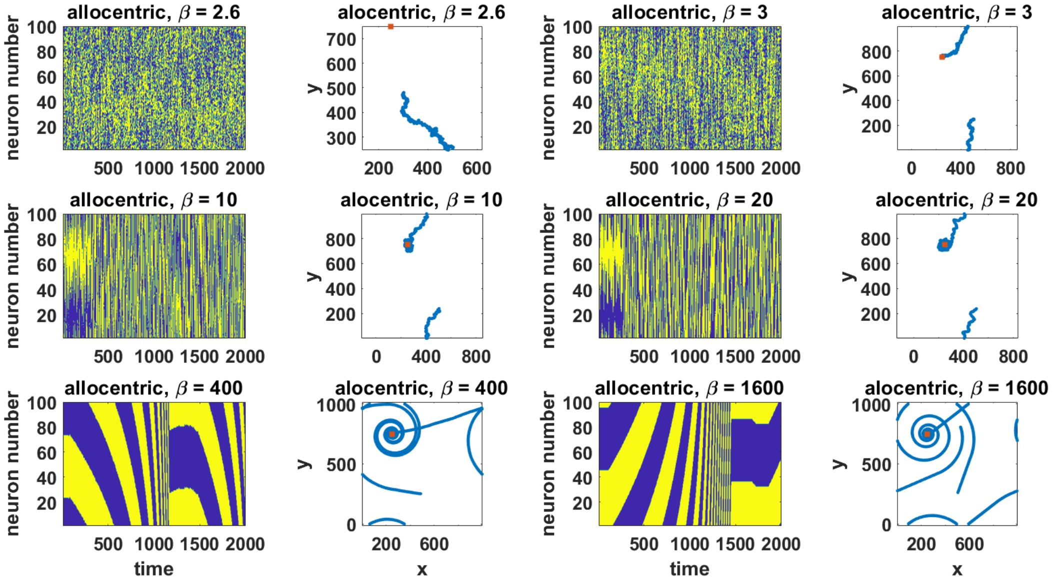

Supplementary Figure. 5: Movement patterns with an allocentric representation of space during tracking a fixed target. In contrast to an egocentric representation of space, external stimuli destabilize a bump of activity. This leads to movement towards the target in a spiral motion for large values of β. For smaller values of β, the agent moves towards the target in a more noisy trajectory due to transitions between states driven by noise. Parameter values: $N_s = 100$, $\sigma = 2\pi/N_s$, $\nu = 0.5$, $v_0 = 10$, $h_0 = 0.01$. The agent moves in a periodic space with linear size, $L = 1000$.

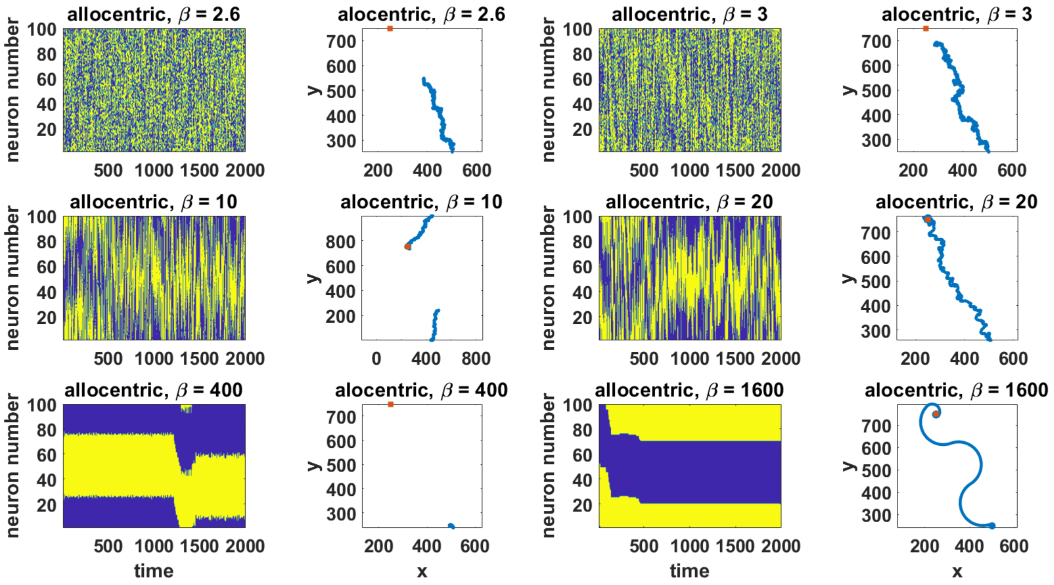

Supplementary Figure. 6: Movement patterns with an egocentric representation of space while tracking a fixed target. In the presence of a target, the agent moves towards the target. Comparison with the case in the absence of a target and for smaller values of β show that in the presence of an ego-centric representation of space, tracking a target leads to more stable bumps. This helps the agent to stay close to a target, once reaches the target. Parameter values: $N_s = 100$, $\sigma = 2\pi/N_s$, $\nu = 0.5$, $v_0 = 10$, $h_0 = 0.01$. The agent moves in a periodic space with linear size, $L = 1000$.

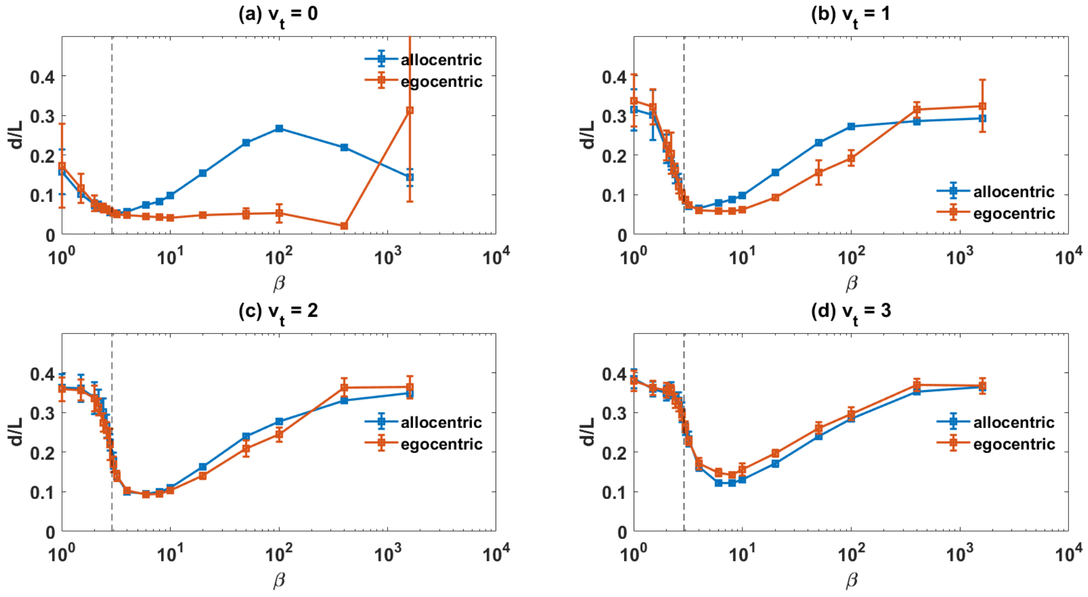

Supplementary Figure. 7: Individual information acquisition (networks of $N_s = 100$ neuron groups). The average distance of an agent from a moving target with an egocentric and an allocentric representation of space is plotted as a function of β for a small value of the target speed, v_t. While for zero or too small target speed, leading to a static or slowly changing environment egocentric representation of space performs better for large β, for larger target speed the agent with an allocentric representation of space performs better. For smaller network size, optimal region may occur at a larger distance to criticality due to the broadening of the critical region in small networks. Parameter values: $N_s = 100$, $\sigma = 2\pi/N_s$. The agent moves in a periodic space with linear size, $L = 100$.

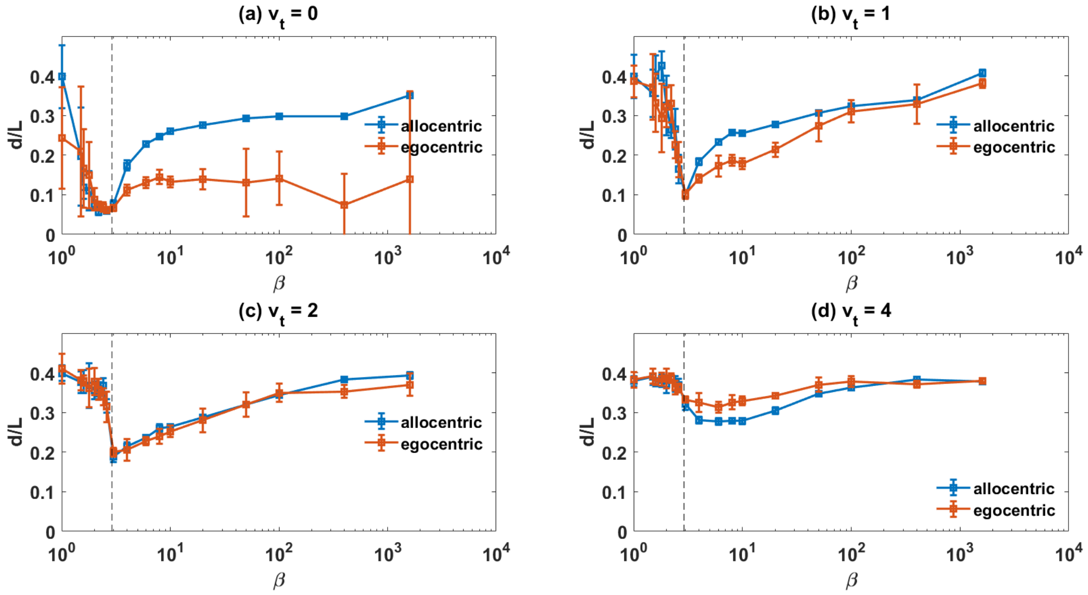

Supplementary Figure. 8: Individual information acquisition (networks of $N_s = 400$ neuron groups). The average distance of an agent from a moving target with an egocentric and an allocentric representation of space as a function of β is plotted for a small value of the target speed, v_t. While for zero or too small target speed, leading to a static or slowly changing environment, egocentric representation of space performs better for large β, for larger target speed the agent with an allocentric representation of space performs better. Compared to smaller networks, optimal information acquisition occurs closer to criticality, due to the fact that the critical region sharpens for larger networks. Parameter values: $N_s = 400$, $\sigma = 2\pi/N_s$. The agent moves in a periodic space with linear size, $L = 100$.

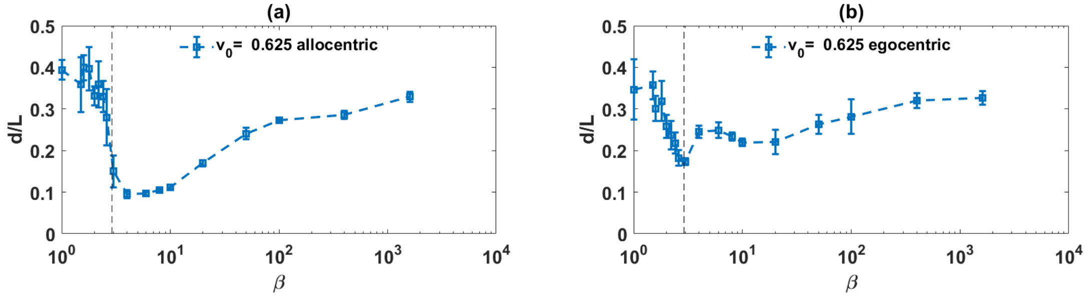

Supplementary Figure. 9: The effect of speed constant, v_0, on individual information acquisition. The average distance of an agent from a moving target with an allocentric (a) and egocentric (b) representation of space is plotted as a function of β for a small value of v_0. A smaller value of v_0 leads to slower movement of the agent and amounts to a scaling of the space size and target speed. Thus, it leads to a higher relative speed of the target with respect to the agent. Consequently, an agent with an allocentric representation of space performs better than an egocentric representation of space due to faster (relative) environmental changes (Compare with panel (b) in Supplementary Figure. 15 and Supplementary Figure. 14 for a larger value of v_0). Parameter values: $N_s = 400$, $\sigma = 2\pi/N_s$. The agent moves in a periodic space with linear size, $L = 100$.

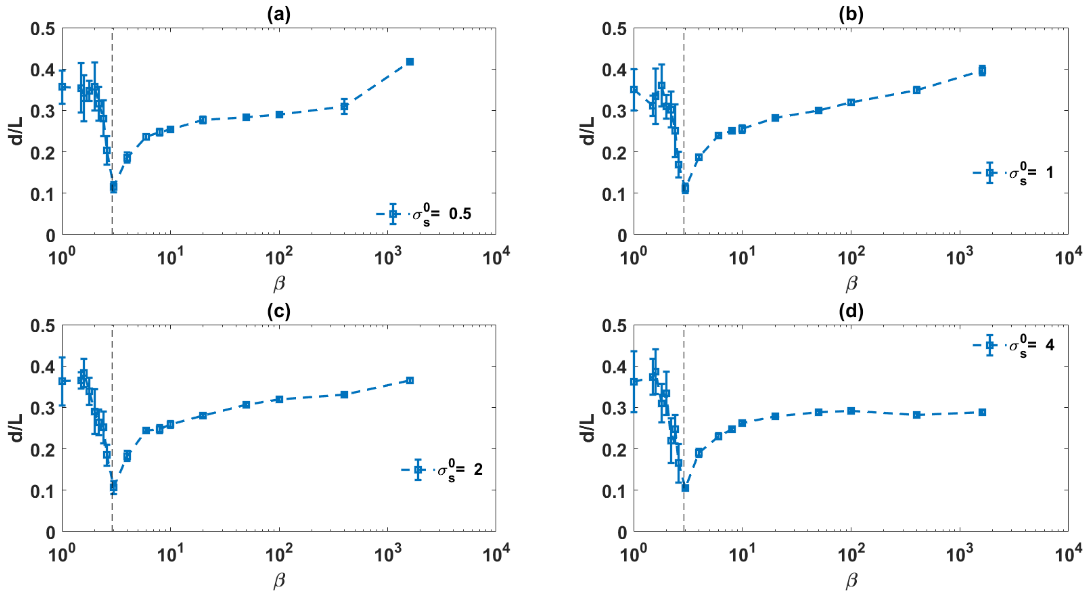

Supplementary Figure. 10: The effect of the receptive field on individual information acquisition with an allocentric representation of space. The average distance of an agent with an allocentric representation of space from a moving target is plotted as a function of β for different values of the width of the receptive field. Here, the receptive field is equal to $\sigma = 2\pi\sigma_s^0/N$. In all the cases, the optimal information acquisition capacity occurs close to criticality. Here, the target performs a random walk with speed $v_t = 1$. The critical point is shown by a dashed line. Parameter values: $N_s = 400$, $\sigma = 2\pi\sigma_s^0/N$, $v_0 = 10$, $h_0 = 0.0025$. The agent moves in a periodic space with linear size, $L = 100$.

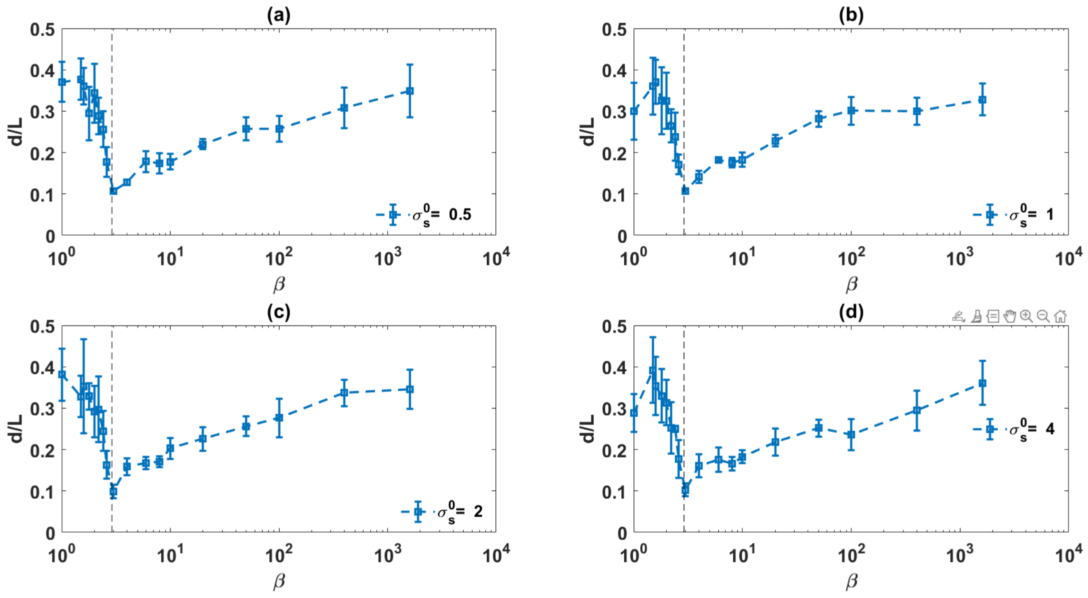

Supplementary Figure. 11: The effect of the receptive field on individual information acquisition with an egocentric representation of space. The average distance of an agent with an egocentric representation of space from a moving target is plotted as a function of β for different values of the width of the receptive field. Here, the receptive field is equal to $\sigma = 2\pi\sigma_s^0/N$. In all the cases, the optimal information acquisition capacity occurs close to criticality. Here, the target performs a random walk with speed $v_t = 1$. The critical point is shown by a dashed line. Parameter values: $N_s = 400$, $\sigma = 2\pi\sigma_s^0/N$, $v_0 = 10$, $h_0 = 0.0025$. The agent moves in a periodic space with linear size, $L = 100$.

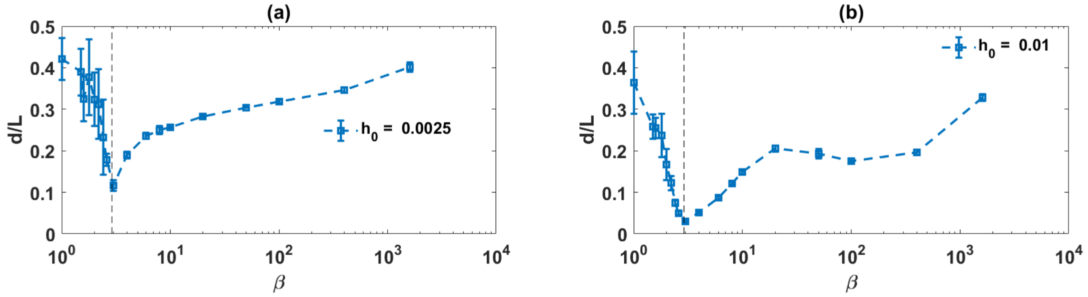

Supplementary Figure. 12: The effect of the amplitude of the external stimuli on individual information acquisition in the presence of an allocentric representation of space. The average distance of an agent with an allocentric representation of space from a moving target is plotted as a function of β for different values of the amplitude of the receptive field, h_0. In all the cases, the optimal information acquisition capacity occurs close to criticality. Here, the target performs a random walk with speed $v_t = 1$. The critical point is shown by a dashed line. Parameter values: $N_s = 400$, $\sigma = 2\pi/N_s$, $v_0 = 10$, $h_0 = 0.0025$. The agent moves in a periodic space with linear size, $L = 100$.

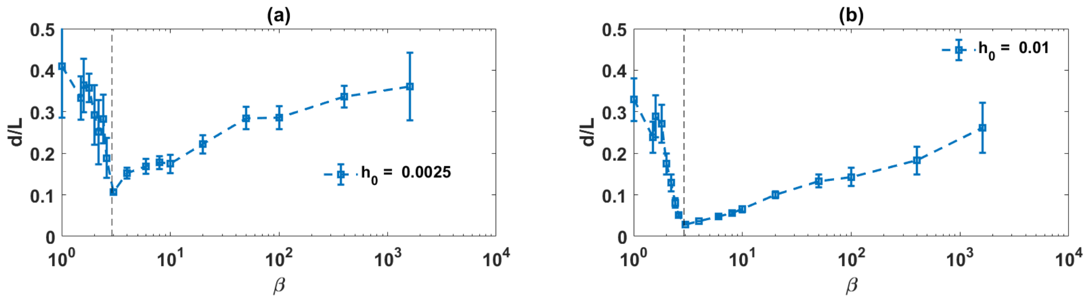

Supplementary Figure. 13: The effect of the amplitude of the external stimuli on individual information acquisition in the presence of an egocentric representation of space. The average distance of an agent with an egocentric representation of space from a moving target is plotted as a function of β for different values of the amplitude of the receptive field, h_0. In all the cases, the optimal information acquisition capacity occurs close to criticality. Here, the target performs a random walk with speed $v_t = 1$. The critical point is shown by a dashed line. Parameter values: $N_s = 400$, $\sigma = 2\pi/N_s$, $v_0 = 10$. The agent moves in a periodic space with linear size, $L = 100$.

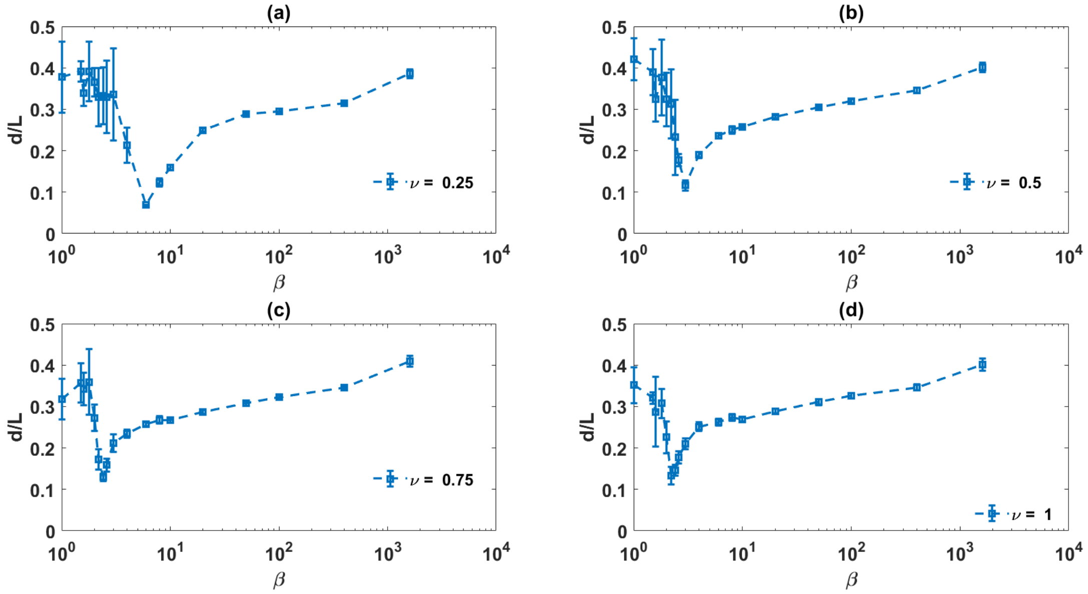

Supplementary Figure. 14: The effect of neural tuning parameter, ν, on individual information acquisition in the presence of an allocentric representation of space. The average distance of an agent with an allocentric representation of space from a moving target is plotted as a function of β for different values of the neural tuning parameter, ν. In all the cases, the optimal information acquisition capacity occurs close to criticality. Here, the target performs a random walk with speed $v_t = 1$. Parameter values: $N_s = 400$, $\sigma = 2\pi/N_s$, $v_0 = 10$. The agent moves in a periodic space with linear size, $L = 100$.

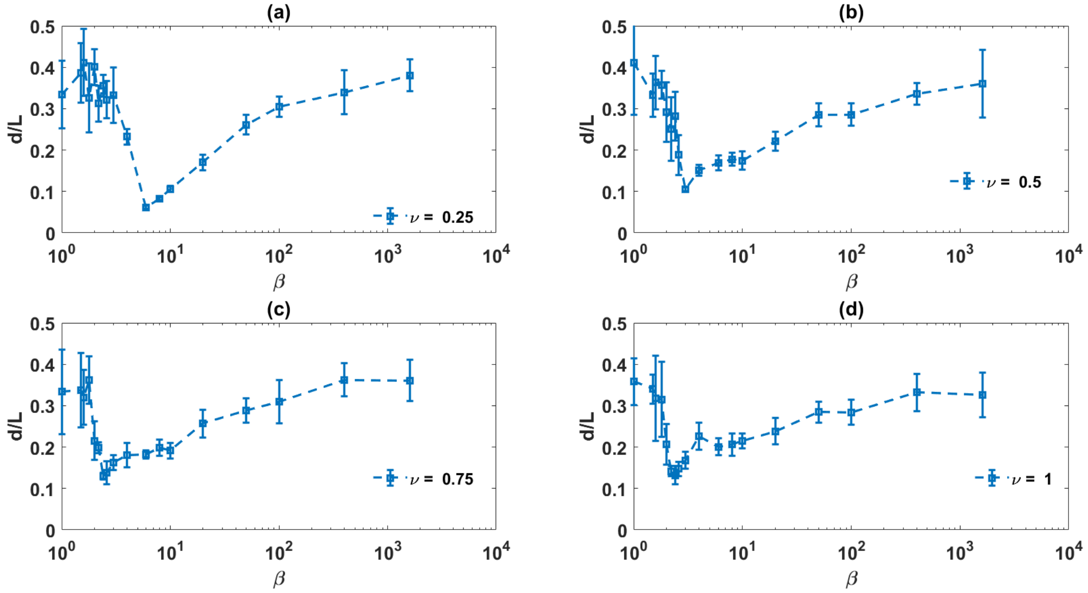

Supplementary Figure. 15: The effect of neural tuning parameter, ν, on individual information acquisition in the presence of an egocentric representation of space. The average distance of an agent with an egocentric representation of space from a moving target is plotted as a function of β for different values of the neural tuning parameter, ν. In all the cases the optimal information acquisition capacity occurs close to criticality. Here the target performs a random walk with speed $v_t = 1$. Parameter values: $N_s = 400$, $\sigma = 2\pi/N_s$, $v_0 = 10$. The agent moves in a periodic space with linear size, $L = 100$.

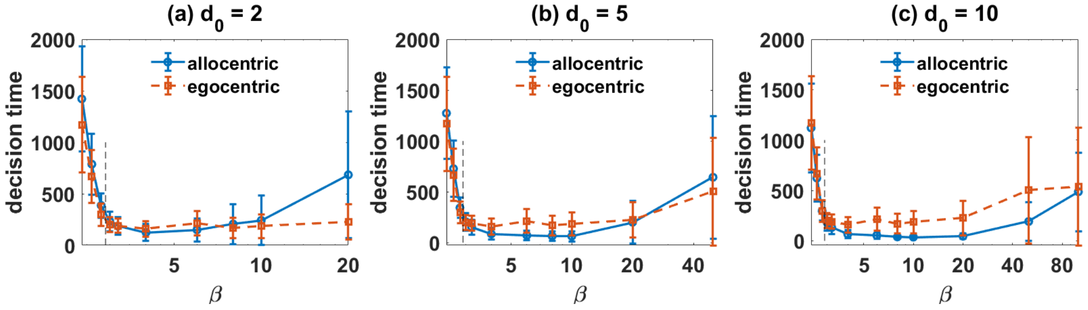

Supplementary Figure. 16: Decision-making speed with an allocentric and egocentric representation of space. The time required for the agent to reach a vicinity, d_0 of a fixed target as a function of β is plotted. There exists an optimal region of β in the ordered phase where the agent decision-making speed is maximized. With an allocentric representation of space, the agent exhibits higher decision-making speed. Smaller d_0 require higher decision-making accuracy, which can increase with an egocentric representation of space. This shows that at smaller distances having an egocentric representation of space is beneficial. The critical point is shown by a dashed line. Parameter values: $N_s = 400$, $\sigma = 2\pi/N_s$, $v_0 = 10$. The agent moves in a periodic space with linear size, $L = 100$. The initial agent position is $x = L/4$ and $y = L/2$ and the target position is $x = L/4$ and $y = 3L/4$. Data points and their error bars show the mean and standard deviation calculated over a sample of 80 simulations.

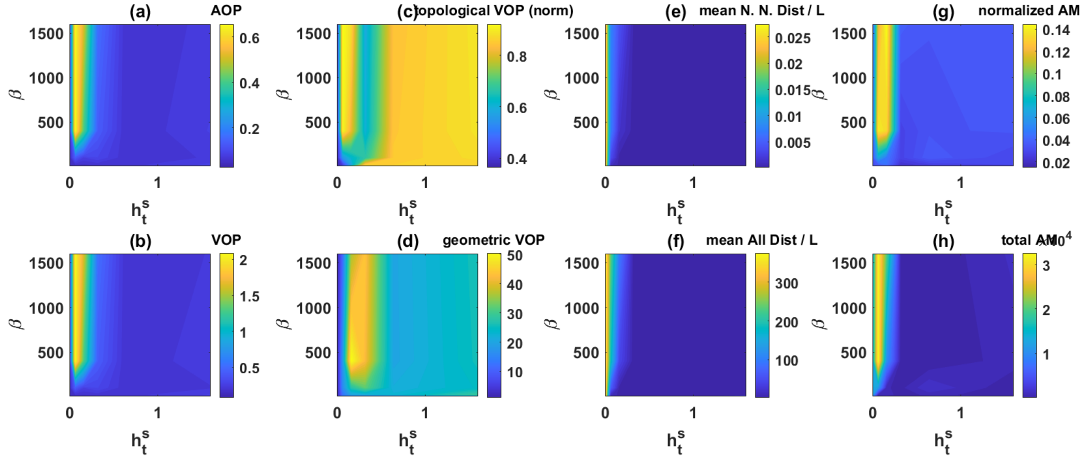

Supplementary Figure. 17: Color plots of collective motion metrics in groups of 320 agents with an allocentric representation of space in $\beta - h_t^s$ space. The collective motion order parameter is color plotted as a function of the total social attraction (h_s^t) and network inverse temperature (β). At zero social attraction, agents exhibit independent movement. An increase in h_s^t triggers a phase transition to ordered motion. Further increasing the social attraction results in agents' coalescence and cohesive motion. (a) to (h) show the global angular order parameter (AOP, (a)), vectorial order parameter (VOP, (b)), local VOP, both topological (c) and geometric (d), mean nearest neighbor distance (e), and all-pair distances (f), normalized and total angular momentum (AM, (g) and (h)). Parameter values: $N_s = 100$, $\sigma = 2\pi/N_s$, $v_0 = 10$. $N = 320$ agents move in a periodic space with linear size, $L = 1000$.

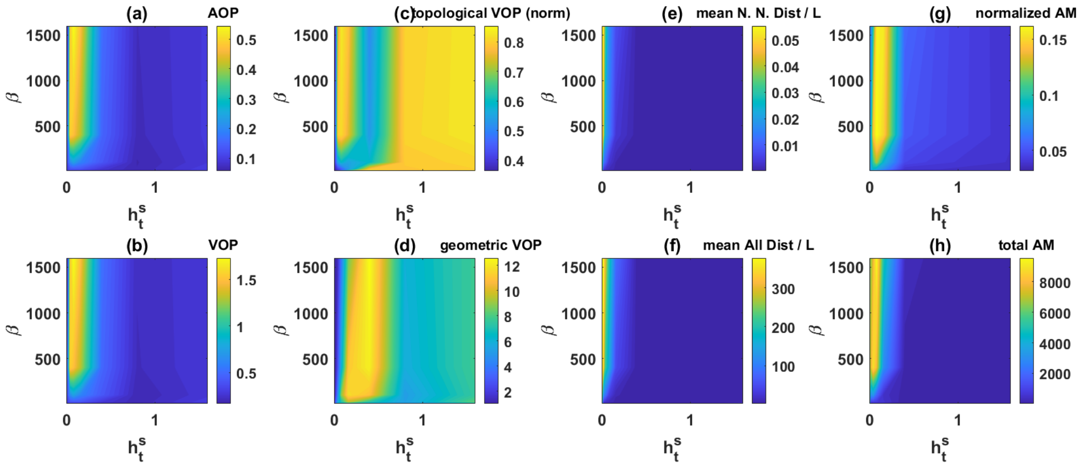

Supplementary Figure. 18: Contour plots of collective motion metrics in $\beta - h_t^s$ space groups of 80 agents with an allocentric representation of space. The collective motion order parameter is color plotted as a function of the total social attraction (h_s^t) and network inverse temperature (β). At zero social attraction, agents exhibit independent movement. An increase in h_s^t triggers a phase transition to ordered motion. Further increasing the social attraction results in agent coalescence and cohesive motion. (a) to (h) show the global angular order parameter (AOP, (a)), vectorial order parameter (VOP, (b)), local VOP, both topological (c) and geometric (d), mean nearest neighbor distance (e), and all-pair distances (f), normalized and total angular momentum (AM, (g) and (h)). Parameter values: $N_s = 100$, $\sigma = 2\pi/N_s$, $v_0 = 10$. $N = 80$ agents move in a periodic space with linear size, $L = 1000$.

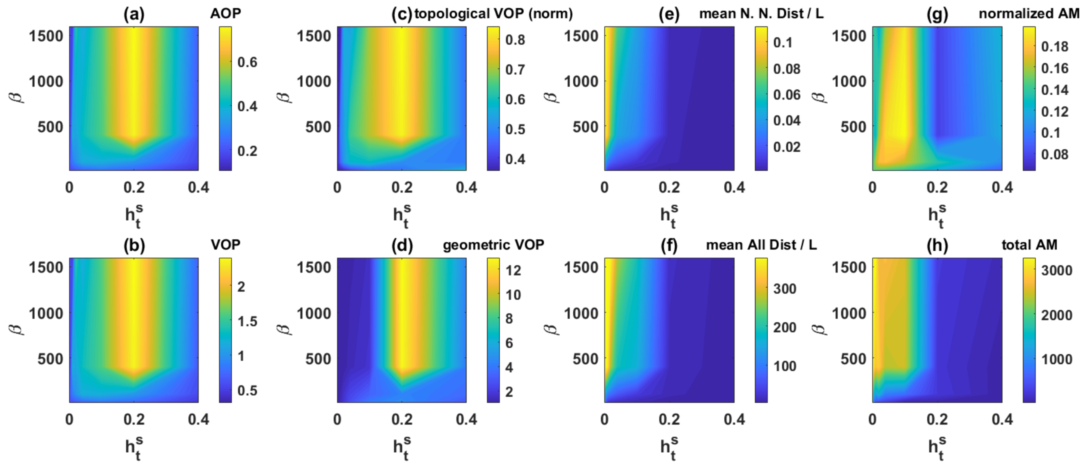

Supplementary Figure. 19: Contour plots of collective motion metrics in groups of 20 agents with an allocentric representation of space in $\beta - h_t^s$ space. The collective motion order parameter is color plotted as a function of the total social attraction (h_s^t) and network inverse temperature (β). At zero social attraction, agents exhibit independent movement. An increase in h_s^t triggers a phase transition to ordered motion. Further increasing the social attraction results in agent coalescence and cohesive motion. (a) to (h) show the global angular order parameter (AOP, (a)), vectorial order parameter (VOP, (b)), local VOP, both topological (c) and geometric (d), mean nearest neighbor distance (e), and all-pair distances (f), normalized and total angular momentum (AM, (g) and (h)). Parameter values: $N_s = 100$, $\sigma = 2\pi/N_s$, $v_0 = 10$. $N = 20$ agents move in a periodic space with linear size, $L = 1000$.

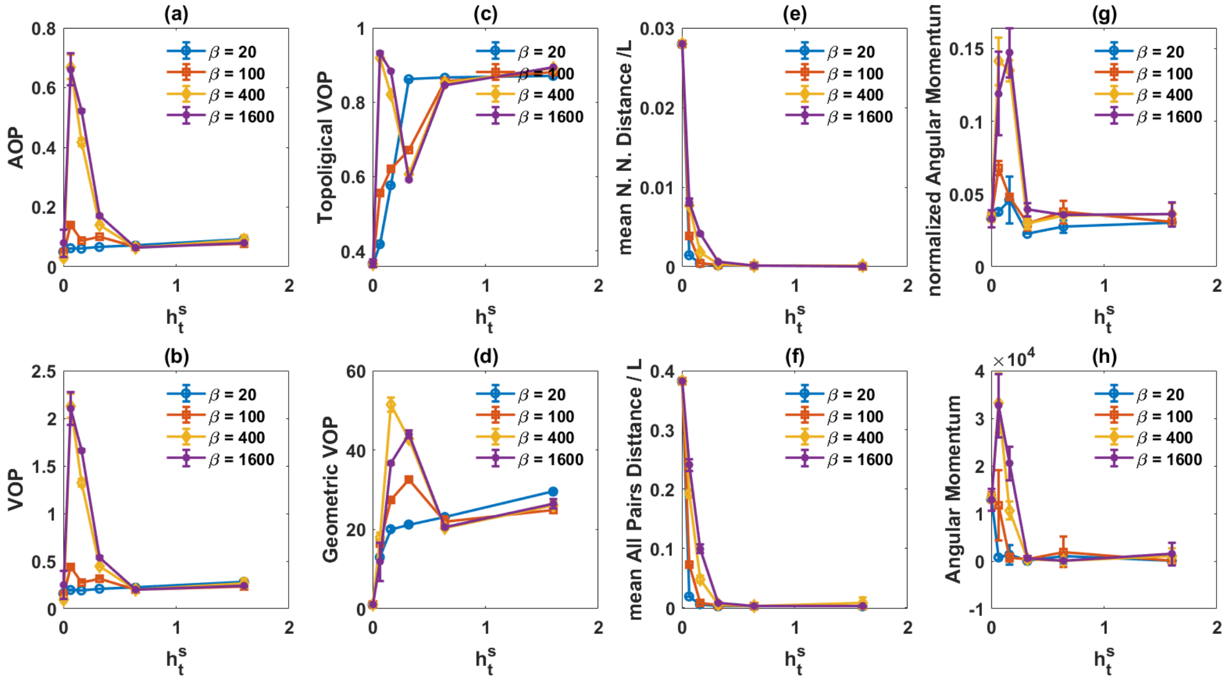

Supplementary Figure. 20: Measures of collective motion in groups of 320 agents with an allocentric representation of space. Different measures of collective motion of agents are shown as a function of the total social attraction, h_s^t, and for different values of β. For zero social attraction, the agents move independently. As the social attraction increases, the system shows a phase transition to a phase where ordered motion is observed. Consequently, the global angular order parameter (AOP in (a)) and vectorial order parameter (VOP in (b)), as well as their local counterparts (topological VOP in (c) and geometric VOP in (d)) increase. Increasing the social sceptibility all the individuals coalesce without net transportative motion. In this phase, the mean nearest neighbor distance (e) and the distance between all pairs (f), as well as the order parameters are minimized. The normalized and total angular momentum (AM, in (g) and (h), respectively) is maximized in the ordered phase, due to the fission-fusion dynamics. Parameter values: $N = 320$. Parameter values: $N_s = 100$, $\sigma = 2\pi/N_s$, $v_0 = 10$. $N = 320$ agents move in a periodic space with linear size, $L = 1000$.

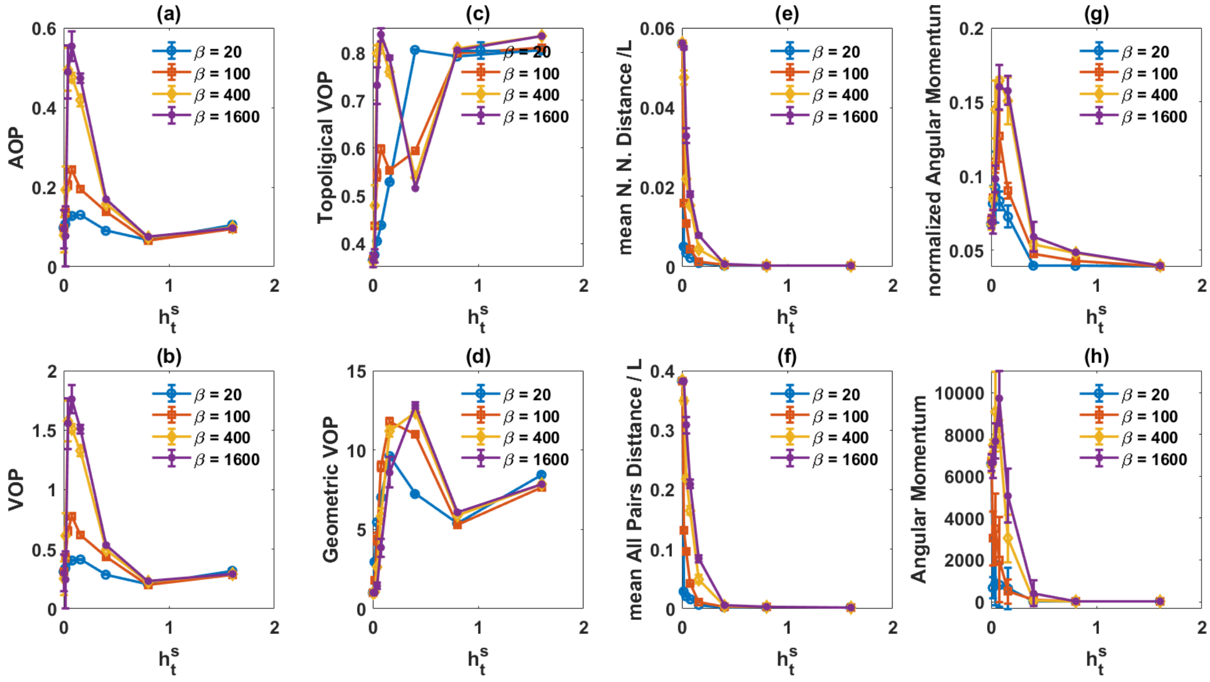

Supplementary Figure. 21: Measures of collective motion in groups of 80 agents with an allocentric representation of space. Different measures of collective motion of agents are shown as a function of the total social attraction, h^t_s, and for different values of β. For zero social attraction, the agents move independently. As the social attraction increases, the system shows a phase transition to a phase where ordered motion is observed. Consequently, the global angular order parameter (AOP in (a)) and vectorial order parameter (VOP in (b)), as well as their local counterparts (topological VOP in (c) and geometric VOP in (d)) increase. Increasing the social sceptibility all the individuals coalesce without net transportative motion. In this phase, the mean nearest neighbor distance (e) and the distance between all pairs (f), as well as the order parameters are minimized. The normalized and total angular momentum (AM, in (g) and (h), respectively) is maximized in the ordered phase, due to the fission-fusion dynamics. Parameter values: $N = 80$. Parameter values: $N_s = 100$, $\sigma = 2\pi/N_s$, $v_0 = 10$. $N = 80$ agents move in a periodic space with linear size, $L = 1000$.

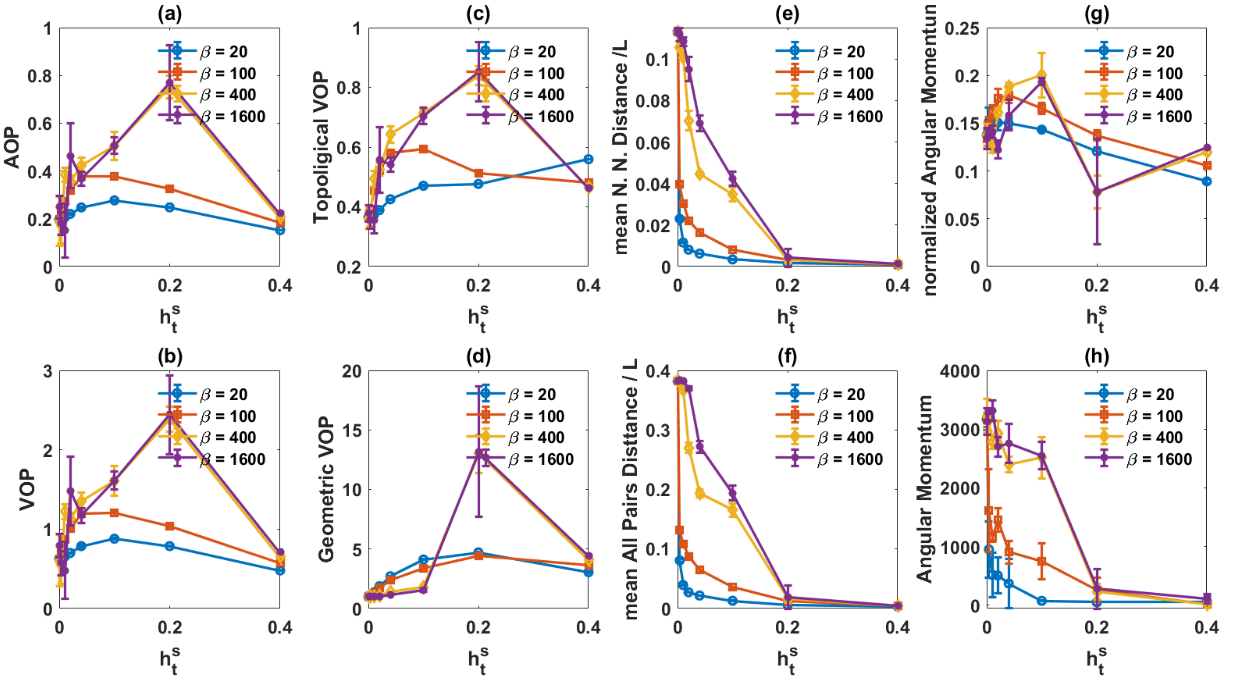

Supplementary Figure. 22: Measures of collective motion in groups of 20 agents with an allocentric representation of space. Different measures of collective motion of agents are shown as a function of the total social attraction, h_s^t, and for different values of β. For zero social attraction, the agents move independently. As the social attraction increases, the system shows a phase transition to a phase where ordered motion is observed. Consequently, the global angular order parameter (AOP in (a)) and vectorial order parameter (VOP in (b)), as well as their local counterparts (topological VOP in (c) and geometric VOP in (d)) increase. Increasing the social sceptibility all the individuals coalesce without net transportative motion. In this phase, the mean nearest neighbor distance (e) and the distance between all pairs (f), as well as the order parameters are minimized. The normalized and total angular momentum (AM, in (g) and (h), respectively) is maximized in the ordered phase, due to the fission-fusion dynamics. Parameter values: $N_s = 100$, $\sigma = 2\pi/N_s$, $v_0 = 10$. $N = 20$ agents move in a periodic space with linear size, $L = 1000$.

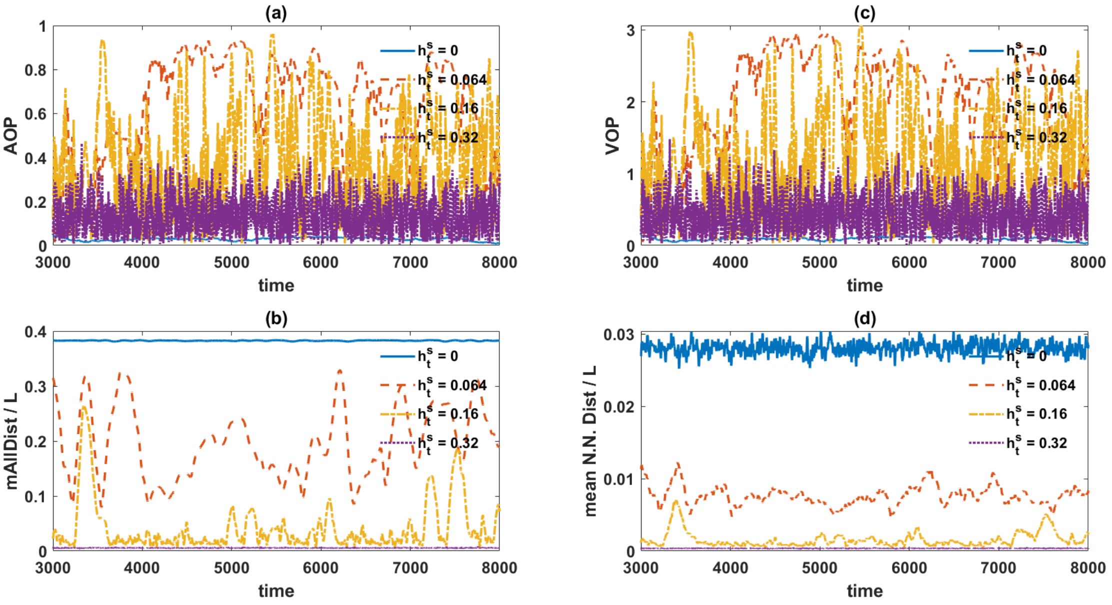

Supplementary Figure. 23: Time dependence of collective movement in groups of 320 agents with an allocentric representation of space. Angular order parameter (AOP in (a)), the mean distance between all the pairs (mAllDist in (b)), vectorial order parameter (VOP in (c)), and mean nearest neighbor distance (N.N. Dist/L in (d)) for four different values of total social attraction indicated in the legend as a function of time is shown. In the absence of social attraction, individuals do not interact and no collective motion is observed. For average social attraction, collective motion is observed. The intermittency indicates the strong fission-fusion dynamics in the system observed in large groups. For larger values of social attraction, $h_s^t = 0.32$, cohesive motion with low global order and low distance between individuals is observed. Parameter values: $N_s = 100$, $\sigma = 2\pi/N_s$, $\nu = 0.5$, $v_0 = 10$, and $\beta = 400$. $N = 320$ agents move in a periodic space with linear size, $L = 1000$.

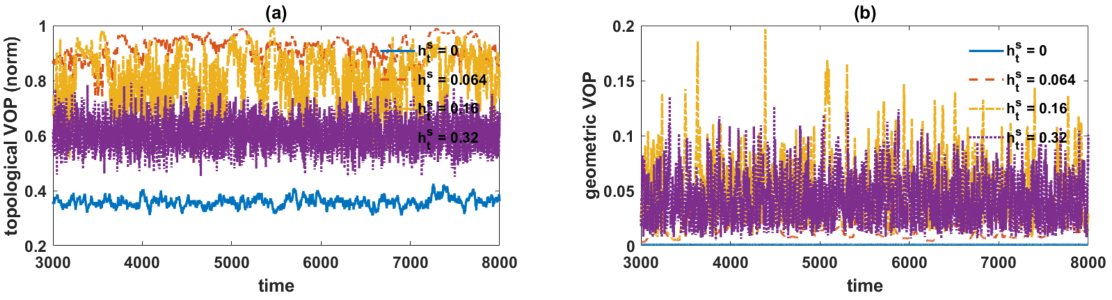

Supplementary Figure. 24: Time dependence of collective movement in groups of 320 agents with an allocentric representation of space. normalized topological vectorial order parameter (VOP in (a)), and the geometric VOP (b) for different values of social attraction are plotted as a function of time. In the absence of social attraction, individuals do not interact and no collective motion is not observed. For $h_s^0 = 0.05$ and $h_s^0 = 0.1$, collective motion is observed, and both topological and geometric VOP increase. For larger values of social attraction, $h_s^t = 0.32$, cohesive motion with low order global order, but persistent local order is observed. A higher value of the geometric VOP in the cohesive phase results from a high number of neighbors (for the distance used here, $R = L/100$) in this phase. Parameter values: $N_s = 100$, $\sigma = 2\pi/N_s$, $\nu = 0.5$, $v_0 = 10$, and $\beta = 400$. $N = 320$ agents move in a periodic space with linear size, $L = 1000$.

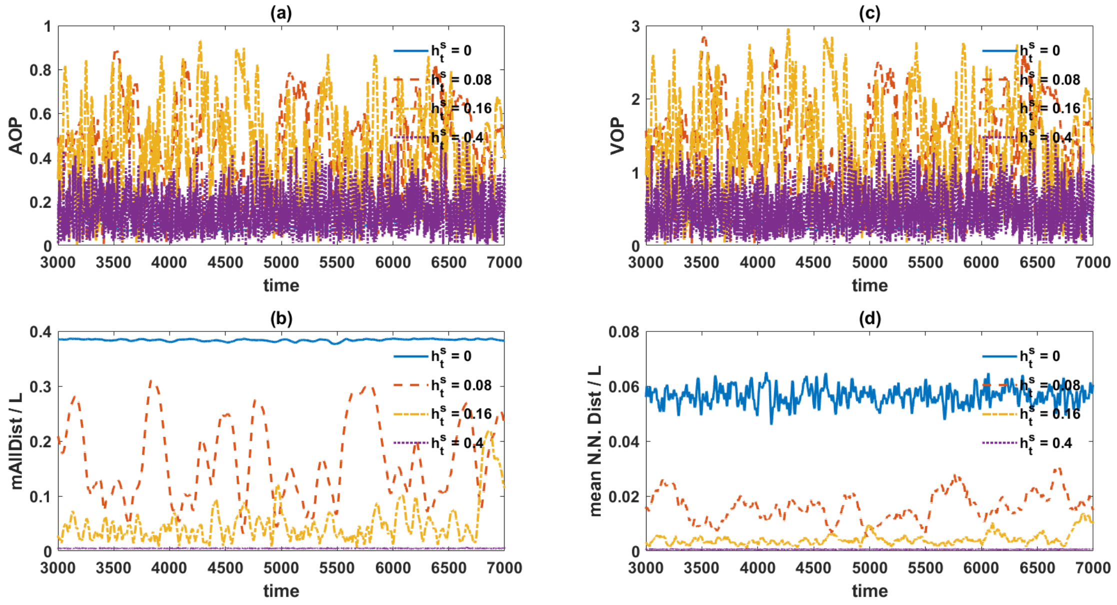

Supplementary Figure. 25: Time dependence of collective movement in groups of 80 agents with an allocentric representation of space. Angular order parameter (AOP in (a)), the mean distance between all the pairs (mAllDist in (b)), vectorial order parameter (VOP in (c)), and mean nearest neighbor distance (N.N. Dist/L in (d)) for four different values of total social attraction indicated in the legend as a function of time is shown. In the absence of social attraction, individuals do not interact and no collective motion is observed. For moderate values of total social attraction, collective motion is observed. The intermittency indicates the strong fission-fusion dynamics in the system observed in large groups. For larger values of social attraction, $h_s^0 = 0.4$, cohesive motion with low global order and low distance between individuals is observed. Parameter values: $N_s = 100$, $\sigma = 2\pi/N_s$, $v_0 = 10$. $N = 80$ agents move in a periodic space with linear size, $L = 1000$, and $\beta = 400$.

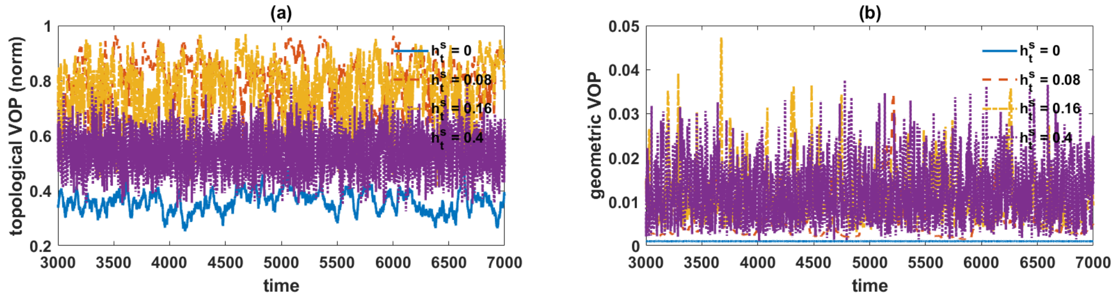

Supplementary Figure. 26: Time dependence of collective movement in groups of 80 agents with an allocentric representation of space. normalized topological vectorial order parameter (VOP in (a)), and the geometric VOP (b) for different values of social attraction are plotted as a function of time. In the absence of social attraction, individuals do not interact and no collective motion is not observed. For $h_s^t = 0.08$ and $h_s^t = 0.16$, collective motion is observed, and both topological and geometric VOP increase. For larger values of social attraction, $h_s^t = 0.4$, cohesive motion with low order global order, but persistent local order is observed. A higher value of the geometric VOP in the cohesive phase results from a high number of neighbors (for the distance used here, $R = L/100$) in this phase. Parameter values: $N_s = 100$, $\sigma = 2\pi/N_s$, $v_0 = 10$. $N = 80$ agents move in a periodic space with linear size, $L = 1000$, and $\beta = 400$.

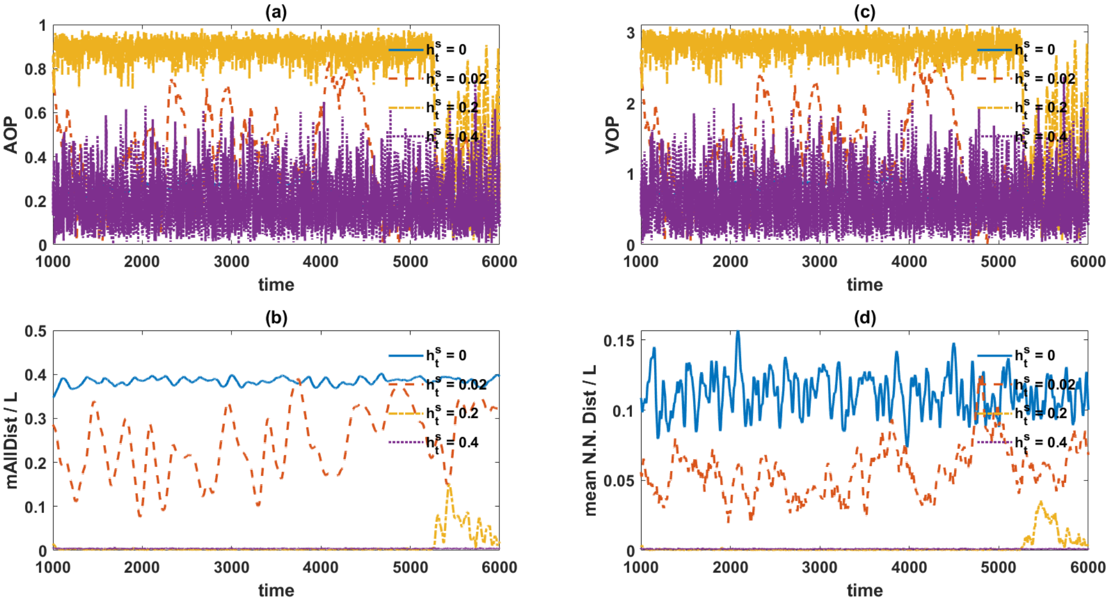

Supplementary Figure. 27: Time dependence of collective movement in groups of 20 agents with an allocentric representation of space. Angular order parameter (AOP in (a)), the mean distance between all the pairs (mAllDist in (b)), vectorial order parameter (VOP in (c)), and mean nearest neighbor distance (N.N. Dist/L in (d)) for four different values of total social attraction indicated in the legend as a function of time is shown. Parameter values: $N_s = 100$, $\sigma = 2\pi/N_s$, $\nu = 0.5$, $v_0 = 10$, and $\beta = 400$. $N = 20$ agents move in a periodic space with linear size, $L = 1000$.

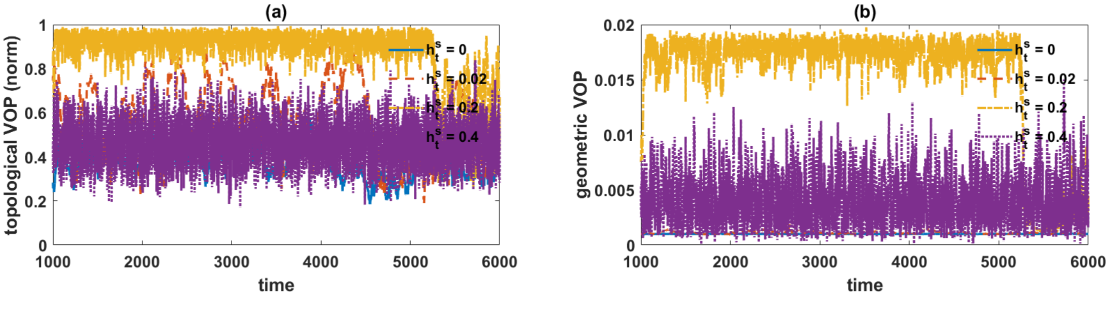

Supplementary Figure. 28: Time dependence of collective movement in groups of 20 agents with an allocentric representation of space. normalized topological vectorial order parameter (VOP in (a)), and the geometric VOP (b) for different values of social attraction are plotted as a function of time. Parameter values: $N_s = 100$, $\sigma = 2\pi/N_s$, $\nu = 0.5$, $v_0 = 10$, and $\beta = 400$. $N = 20$ agents move in a periodic space with linear size, $L = 1000$.

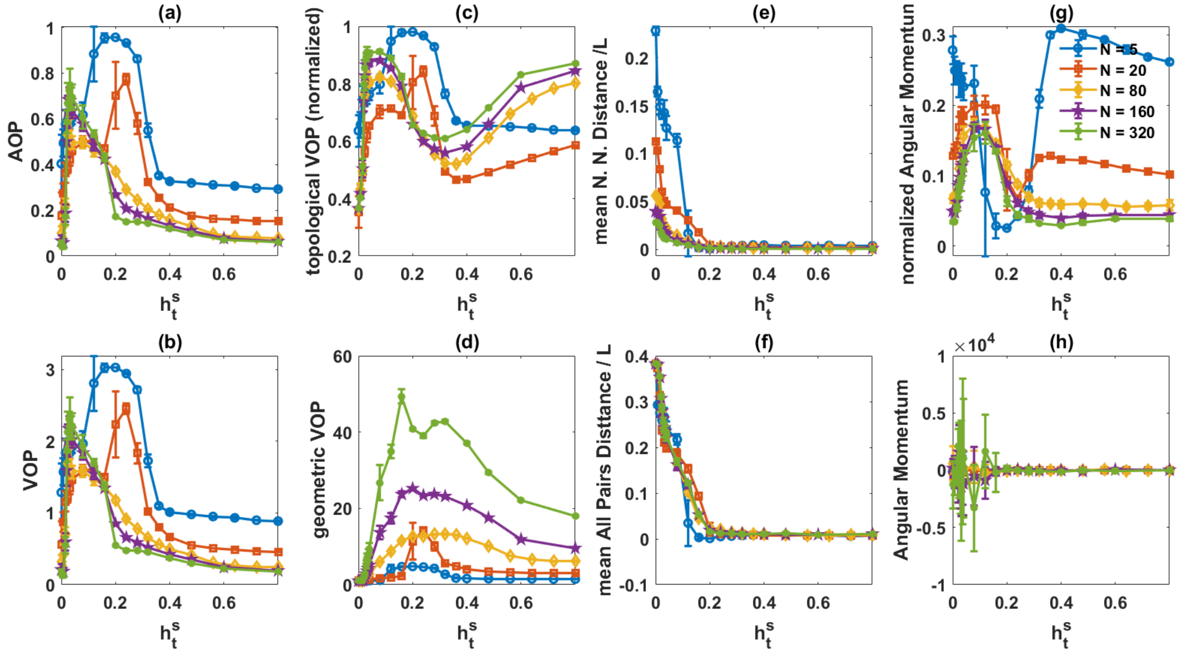

Supplementary Figure. 29: Collective motion of agents with an allocentric representation of space. Different measures of collective motion in groups of agents with various population sizes as a function of the total social attraction, h_t^s, are shown. For zero social attraction, the agents move independently. As the social attraction increases, the system shows two successive phase transitions, first to a phase where ordered motion is observed, and then to a phase where cohesive motion is observed. Consequently, the global angular order parameter (AOP in (a)) and vectorial order parameter (VOP in (b)), as well as local VOP (topological VOP in (c) and geometric VOP in (d)) increases. By further increasing the social sceptibility all the individuals coalesce without net transportative motion. In this phase, the mean nearest neighbor distance (e) and the distance (f) between all pairs, as well as the global order parameters are minimized. However, local ordering is still observed. For large groups, the normalized and total angular momentum (AM, in (g) and (h), respectively) is maximized in the ordered phase, due to the fission-fusion dynamics. Parameter values: $N_s = 100$, $\sigma = 2\pi/N_s$, $\nu = 0.5$, $v_0 = 10$, and $\beta = 400$. Agents move in a periodic space with linear size, $L = 1000$.

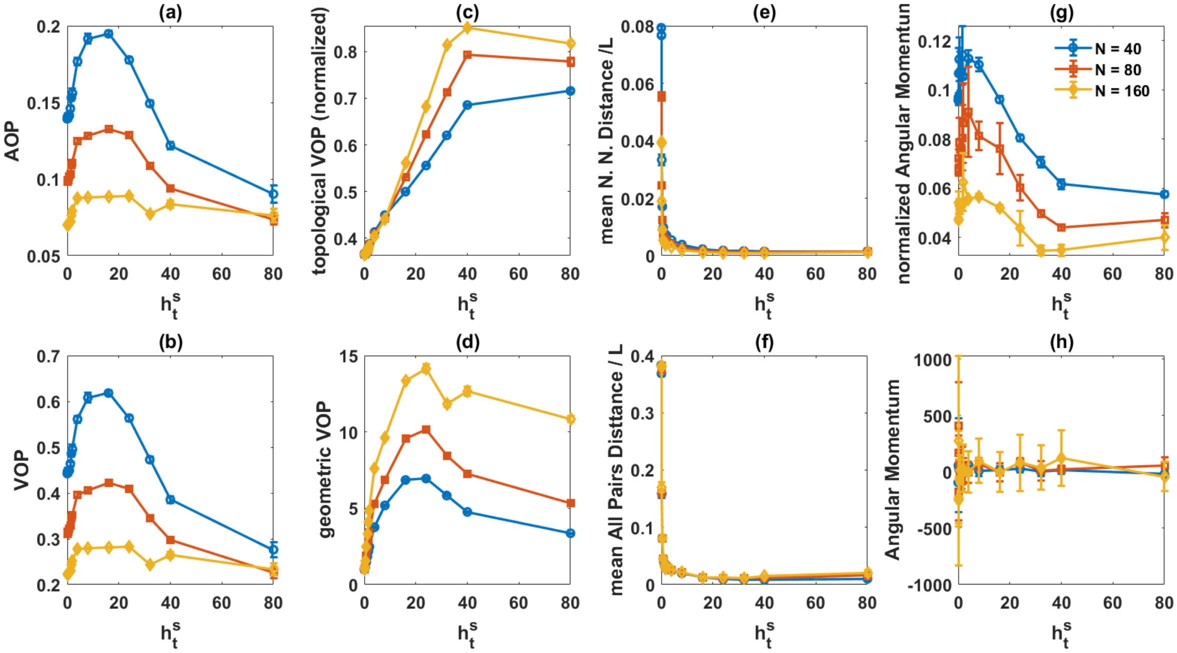

Supplementary Figure. 30: Lack of collective motion for small values of β. Different measures of collective motion in groups of agents with various population sizes as a function of the total social attraction, h_t^s, for a small value of β, $\beta = 20$, are shown. For zero social attraction, the agents move independently. As the social attraction increases, the system shows a cross-over to the cohesive motion phase. The lack of a singular phase transition is apparent in the fact that the topological order parameter increases slowly as total social attraction increases ((c) and (d)). Furthermore, while in intermediate group size global order is higher, it decreases for too large group sizes ((a) and (b)). The mean distance between agents decreases by increasing the group size ((g) and (h)), resulting from the gravitation nature of the social force, leading to more packed groups for larger group sizes. Parameter values: $N_s = 100$, $\sigma = 2\pi/N_s$, $\nu = 0.5$, $v_0 = 10$, and $\beta = 20$. Agents move in a periodic space with linear size, $L = 1000$. Agents employ an allocentric representation of space.

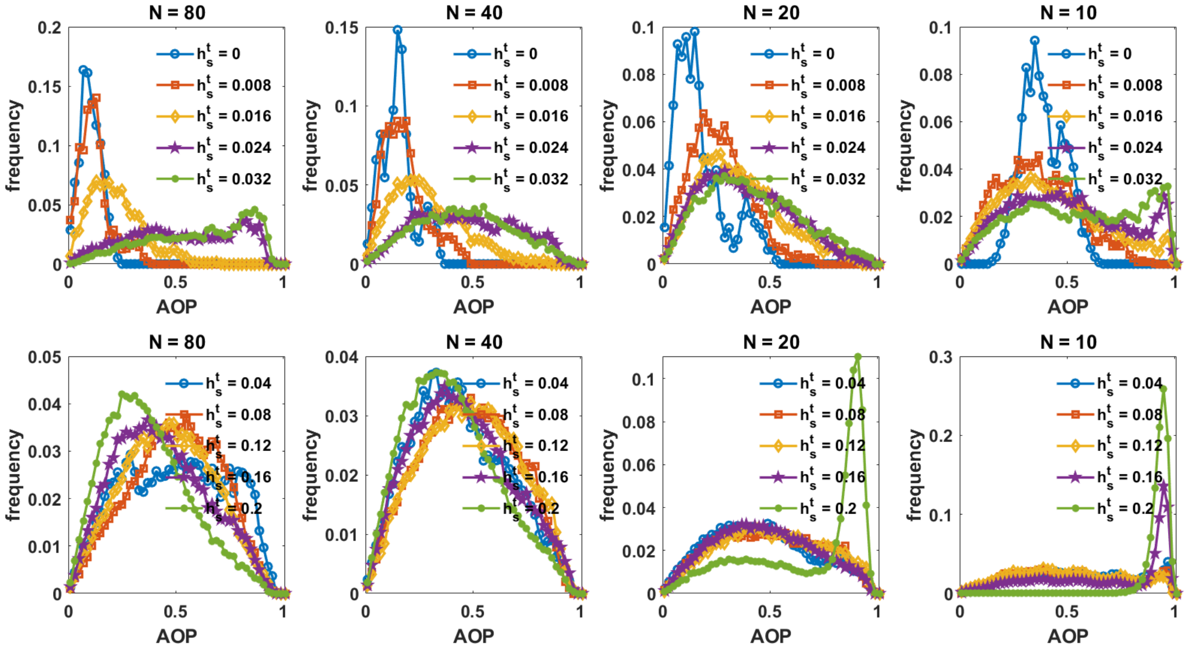

Supplementary Figure. 31: The distribution of Global Order (angular order parameter, AOP) in groups of agents with an allocentric representation of space and in groups of different population sizes. Each panel shows groups of a given size shown on the panel. Total social attraction is shown in the legend. Each group size is broken into two panels to increase visibility. The top rows show the order-disorder phase transition by increasing the social attraction. While the transition appears discontinuous in small groups due to the presence of two peaks corresponding to the ordered and disordered phase, this transition becomes continuous as the group size increases. Further increasing the social attraction, the topological VOP decreases and the system shows another phase transition to the cohesive phase. The collective motion-cohesive motion phase transition occurs continuously in both small and large groups. Parameter values: $N_s = 100$, $\sigma = 2\pi/N_s$, $\nu = 0.5$, $v_0 = 10$, and $\beta = 400$. Agents move in a periodic space with linear size, $L = 1000$.

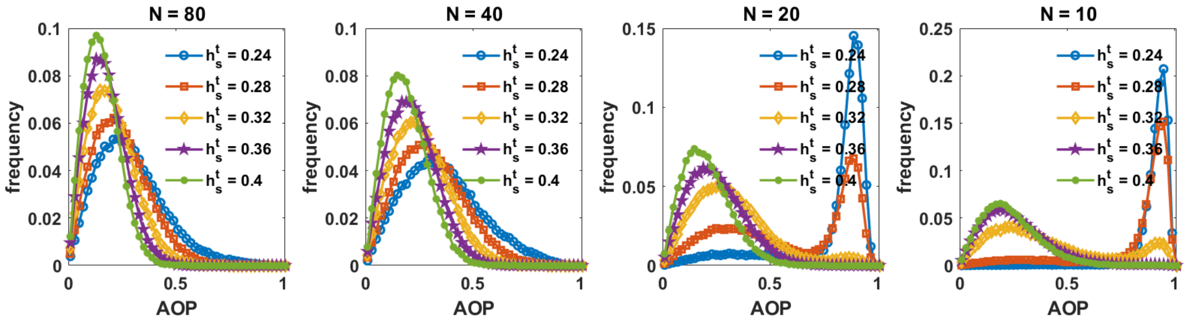

Supplementary Figure. 32: The distribution of Global Order (angular order parameter, AOP) in groups of agents with an allocentric representation of space and in groups of different population sizes. Each panel shows groups of a given size shown on the panel. Total social attraction is shown in the legend. Here, the collective motion-cohesive motion phase transition by increasing social attraction is examined. Parameter values: $N_s = 100$, $\sigma = 2\pi/N_s$, $\nu = 0.5$, $v_0 = 10$, and $\beta = 400$. Agents move in a periodic space with linear size, $L = 1000$.

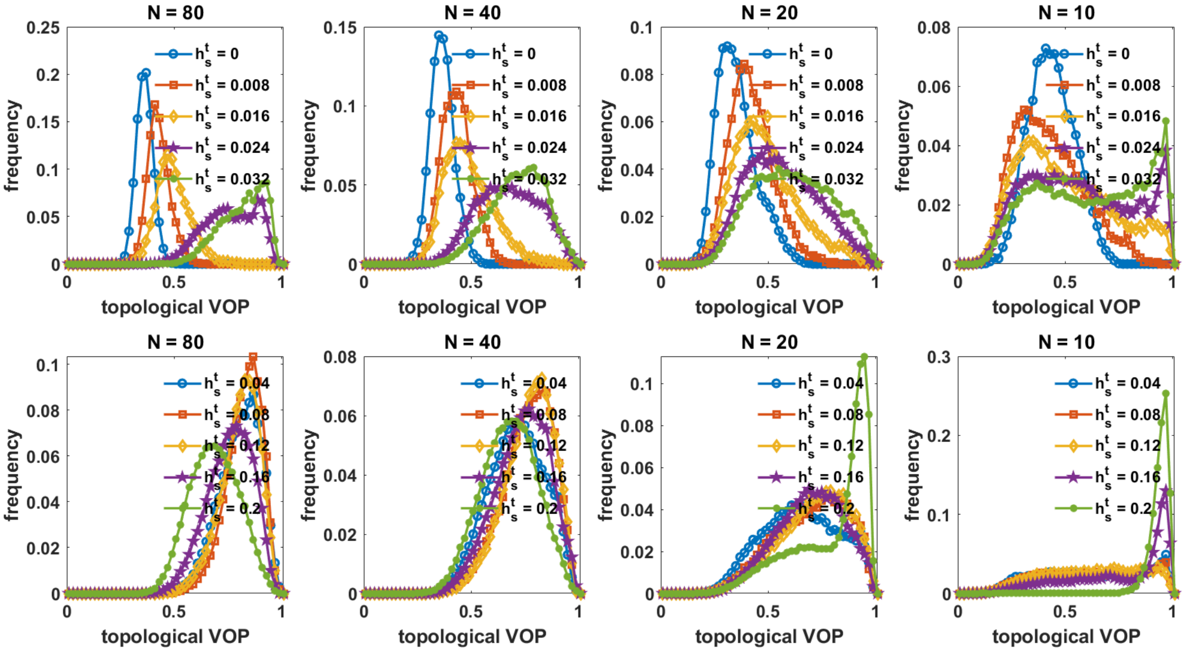

Supplementary Figure. 33: The distribution of Local Order (topological vectorial order parameter) in groups of agents with an allocentric representation of space and in groups of different population sizes. Each panel shows groups of a given size shown on the panel. Total social attraction is shown in the legend. Each group size is broken into two panels to increase visibility. The top rows show the order-disorder phase transition by increasing the social attraction. While the transition appears discontinuous in small groups due to the presence of two peaks corresponding to the ordered and disordered phase, this transition becomes continuous as the group size increases. Further increasing the social attraction, the topological VOP decreases and the system shows another phase transition to the cohesive phase. The collective motion-cohesive motion phase transition occurs continuously in both small and large groups. Parameter values: $N_s = 100$, $\sigma = 2\pi/N_s$, $\nu = 0.5$, $v_0 = 10$, and $\beta = 400$. Agents move in a periodic space with linear size, $L = 1000$.

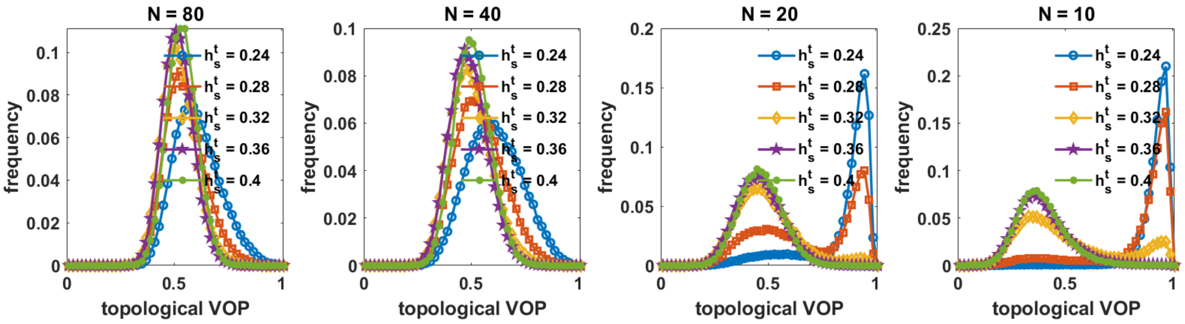

Supplementary Figure. 34: The distribution of Local Order (topological vectorial order parameter, VOP) in groups of agents with an allocentric representation of space and in groups of different size. Each panel shows groups of a given size shown on the panel. Total social attraction is shown in the legend. Here, the collective motion-cohesive motion phase transition by increasing social attraction is examined. Parameter values: $N_s = 100$, $\sigma = 2\pi/N_s$, $\nu = 0.5$, $v_0 = 10$, and $\beta = 400$. Agents move in a periodic space with linear size, $L = 1000$.

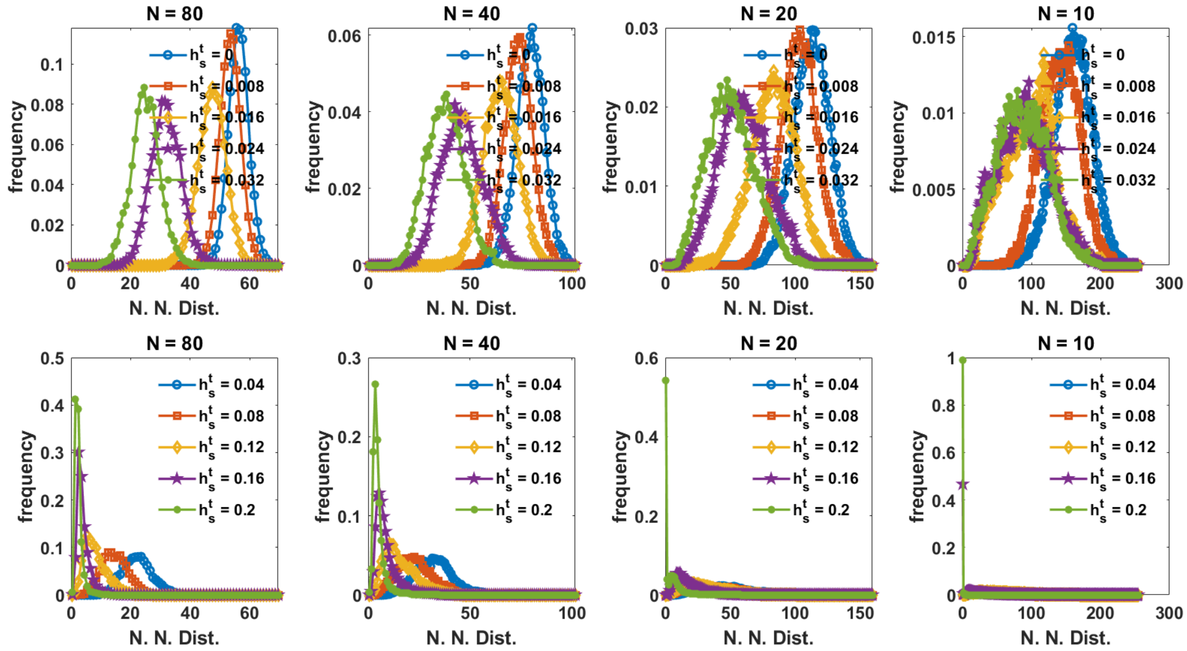

Supplementary Figure. 35: The distribution of mean nearest neighbor distance in groups of agents with an allocentric representation of space and for different population sizes. Each panel shows groups of a given size shown on the panel. Total social attraction is shown in the legend. Each group size is broken into two panels to increase visibility. By increasing the social attraction the system shows an order-disorder transition from a disordered phase where agents move independently, to an ordered phase where collective motion is observed. In the ordered phase, the mean nearest neighbor distance decreases. The transition appears continuously in terms of the spatial distance between agents. Further increasing the social attraction, a phase transition to a cohesive phase with a small distance between agents is observed. Parameter values: $N_s = 100$, $\sigma = 2\pi/N_s$, $\nu = 0.5$, $v_0 = 10$, and $\beta = 400$. Agents move in a periodic space with linear size, $L = 1000$.

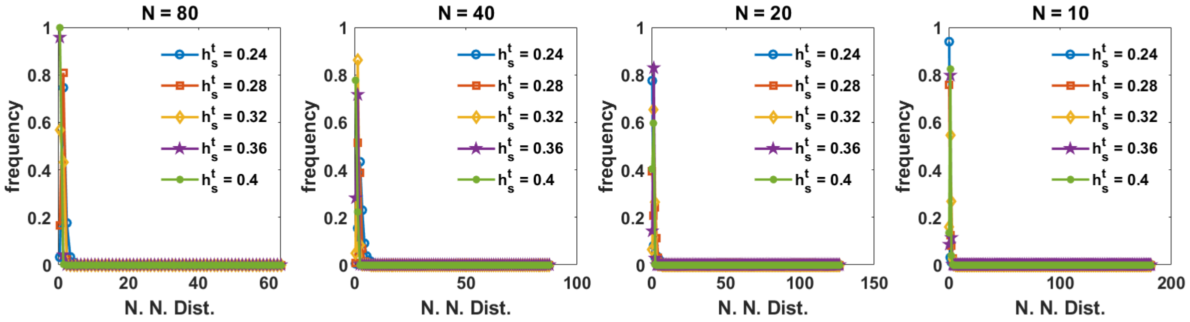

Supplementary Figure. 36: The distribution of mean nearest neighbor distance in groups of agents with an allocentric representation of space and in with different population sizes. Each panel shows groups of a given size shown on the panel. Total social attraction is shown in the legend. Each group size is broken into two panels to increase visibility. Parameter values: $N_s = 100$, $\sigma = 2\pi/N_s$, $\nu = 0.5$, $v_0 = 10$, and $\beta = 400$. Agents move in a periodic space with linear size, $L = 1000$.

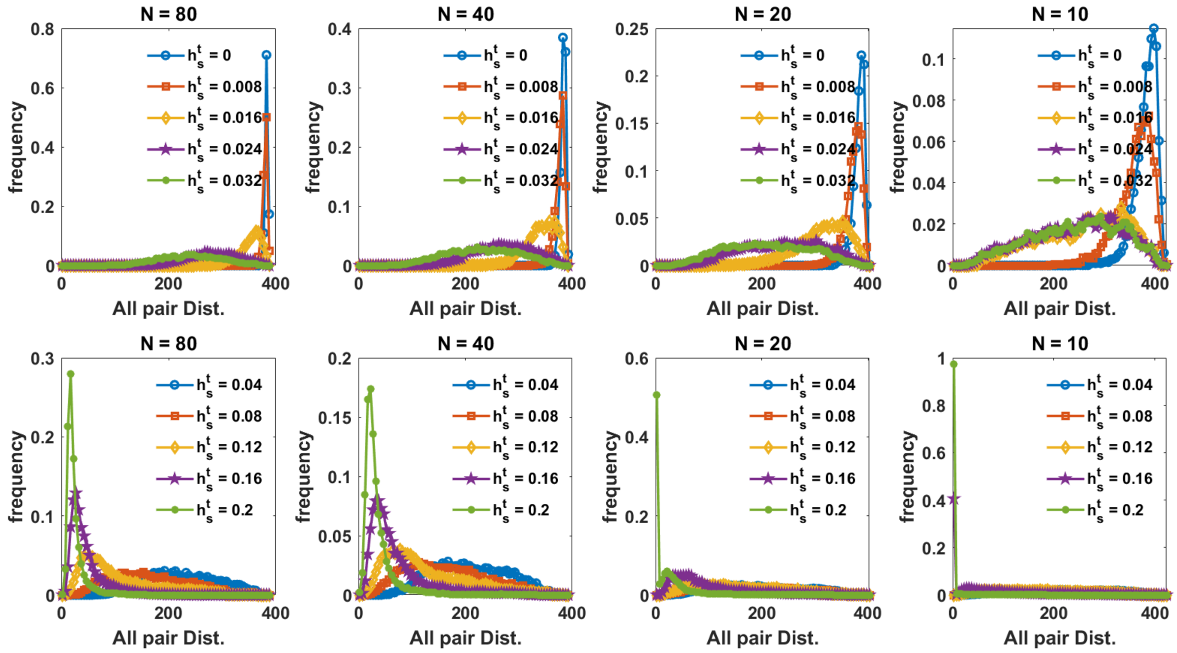

Supplementary Figure. 37: The distribution of mean distance between all pairs of individuals in groups of agents with an allocentric representation of space and with different population sizes. Each panel shows groups of a given size shown on the panel. Total social attraction is shown in the legend. Each group size is broken into two panels to increase visibility. By increasing the social attraction the system shows an order-disorder transition from a disordered phase where agents move independently, to an ordered phase where collective motion is observed. In the ordered phase, the mean distance between individuals decreases. The transition appears continuously in terms of the spatial distance between agents. Further increasing the social attraction, a phase transition to a cohesive phase with a small distance between agents is observed. Parameter values: $N_s = 100$, $\sigma = 2\pi/N_s$, $\nu = 0.5$, $v_0 = 10$, and $\beta = 400$. Agents move in a periodic space with linear size, $L = 1000$.

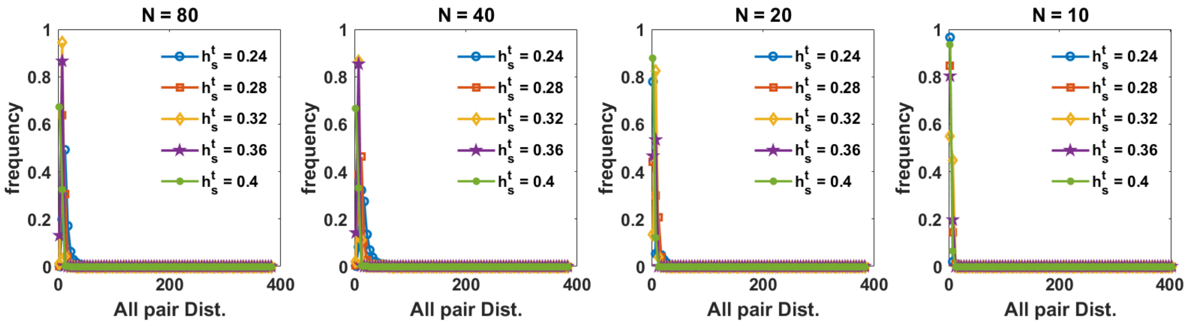

Supplementary Figure. 38: The distribution of mean distance between all pairs in groups of agents with an allocentric representation of space and with different population sizes. Each panel shows groups of a given size shown on the panel. Total social attraction is shown in the legend. Each group size is broken into two panels to increase visibility. Parameter values: $N_s = 100$, $\sigma = 2\pi/N_s$, $\nu = 0.5$, $v_0 = 10$, and $\beta = 400$. Agents move in a periodic space with linear size, $L = 1000$.

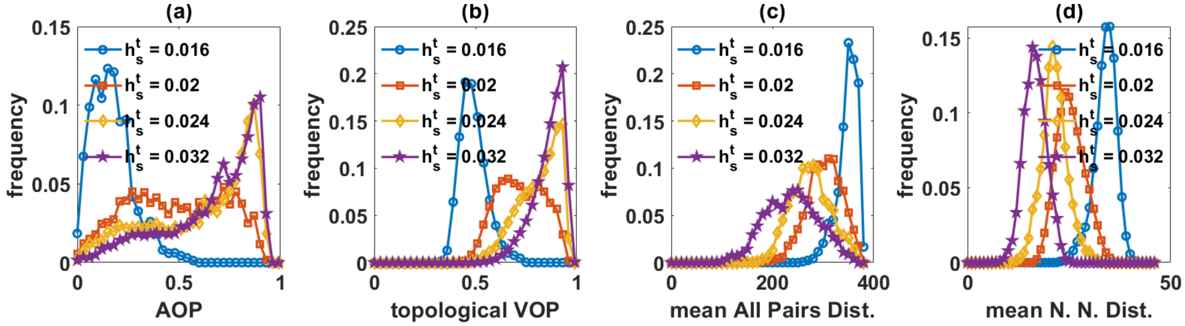

Supplementary Figure. 39: The distribution of Global Order (angular order parameters in (a) and (e)), Local order (normalized topological order parameters in (b) and (f)), mean distance between all pairs (c) and (g), and mean nearest neighbor distance, (d) and (h), in groups of 160 agents with an allocentric representation of space close to the order-disorder (a) to (d) and collective motion-cohesive motion phase transition (e) to (h). Total social attraction is shown in the legend. Parameter values: $N_s = 100$, $\sigma = 2\pi/N_s$, $\nu = 0.5$, $v_0 = 10$, and $\beta = 400$. 160 agents move in a periodic space with linear size, $L = 1000$.

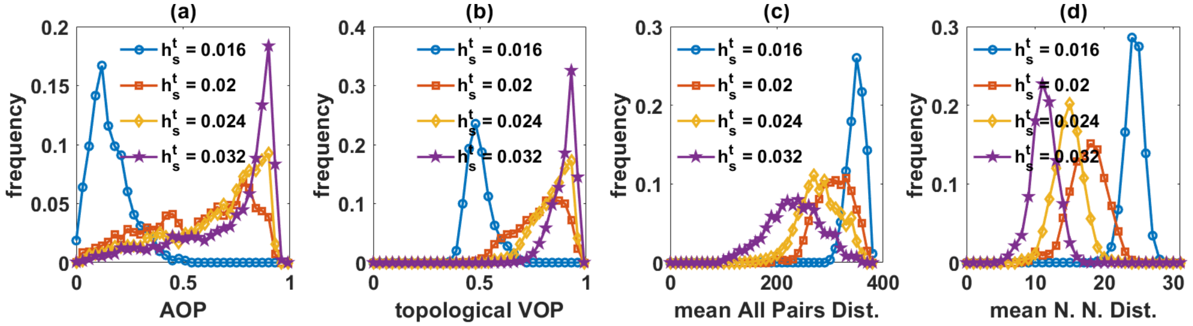

Supplementary Figure. 40: The distribution of Global Order (angular order parameters in (a) and (e)), Local Order (normalized topological order parameters in (b) and (f)), mean distance between all pairs (c) and (g), and mean nearest neighbor distance, (d) and (h), in groups of 320 agents with an allocentric representation of space close to the order-disorder, (a) to (d), and collective motion-cohesive motion phase transition (e) to (h). Total social attraction is shown in the legend. Parameter values: $N_s = 100$, $\sigma = 2\pi/N_s$, $\nu = 0.5$, $v_0 = 10$, and $\beta = 400$. 320 agents move in a periodic space with linear size, $L = 1000$.

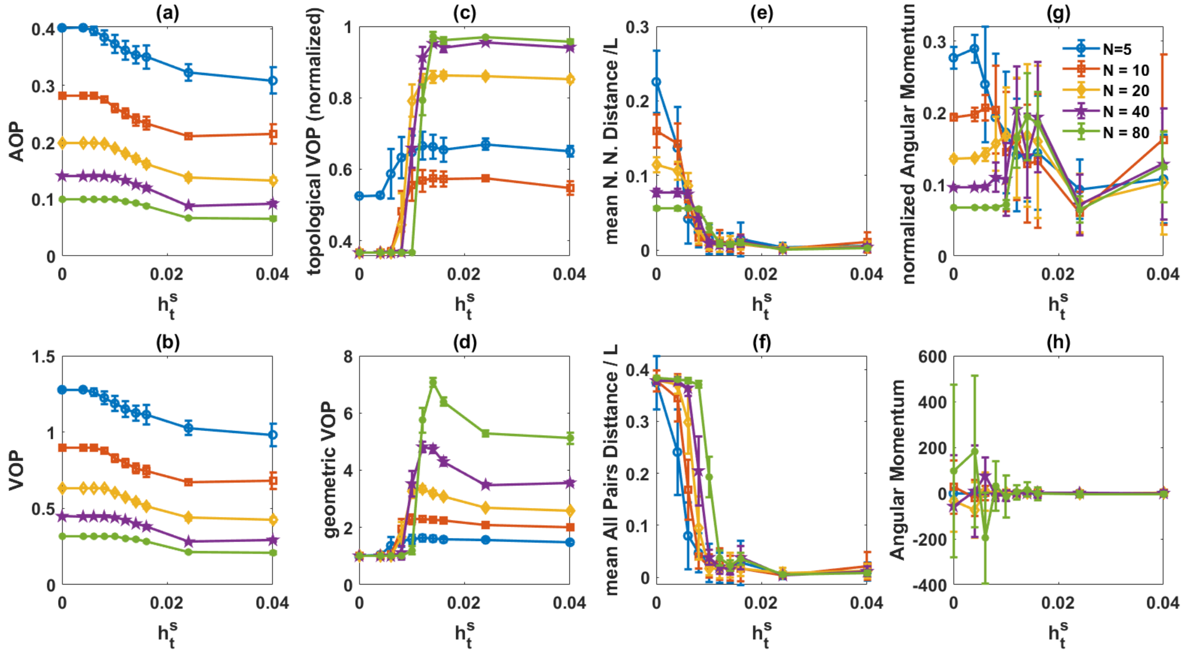

Supplementary Figure. 41: Order-disorder transition in agents with an egocentric representation of space. Different measures of collective motion in groups of agents with an egocentric representation of space and with various population sizes as a function of the total social attraction, h_t^s, are shown. As social attraction increases, the groups show an order-disorder transition. However, collective motion is not observed, and both the angular order parameter (AOP in (a)) and vectorial order parameter (VOP in (b)), as measures of global order, remain small and approach zero in large groups. However, a high ordering of agents' head direction is observed in the ordered phase. Consequently, both topological (c) and geometric (d) VOP (measures of local order) take high values. Furthermore, the distance between agents decreases in the ordered phase and is independent of group size for large social attraction. A small, but non-zero angular momentum is observed in the ordered phase. Topological VOP for $N = 5$ is calculated based the the first 2 nearest neighbor and in all the other cases it is calculated based on the first 5 nearest neighbors. Parameter values: $N_s = 100$, $\sigma = 2\pi/N_s$, $\nu = 0.5$, $v_0 = 10$, and $\beta = 400$. Agents move in a periodic space with linear size, $L = 1000$.

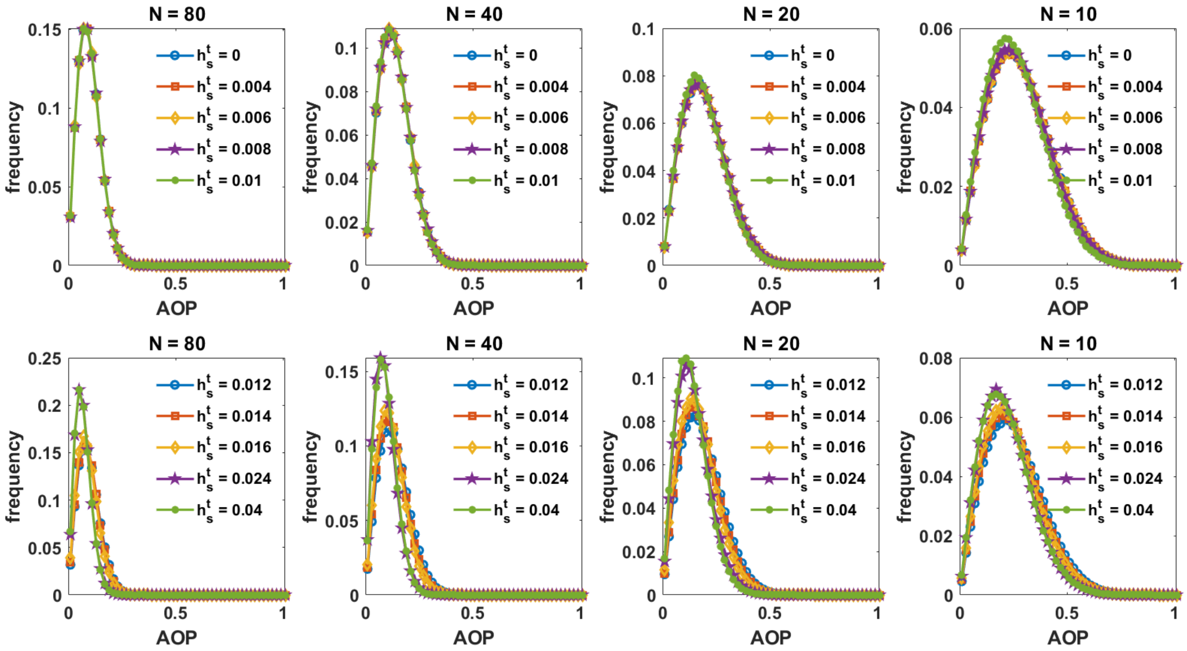

Supplementary Figure. 42: The distribution of Global Order (angular order parameter, AOP) in groups of agents with an egocentric representation of space and in groups of various population sizes. Each panel shows groups of a given size shown on the panel. Total social attraction is shown in the legend. Each group size is broken into two panels to increase visibility. The global order remains low for all the social susceptibilities, indicating collective motion does not exist with an egocentric coordinate. Parameter values: $N_s = 100$, $\sigma = 2\pi/N_s$, $\nu = 0.5$, $v_0 = 10$, and $\beta = 400$. Agents move in a periodic space with linear size, $L = 1000$.

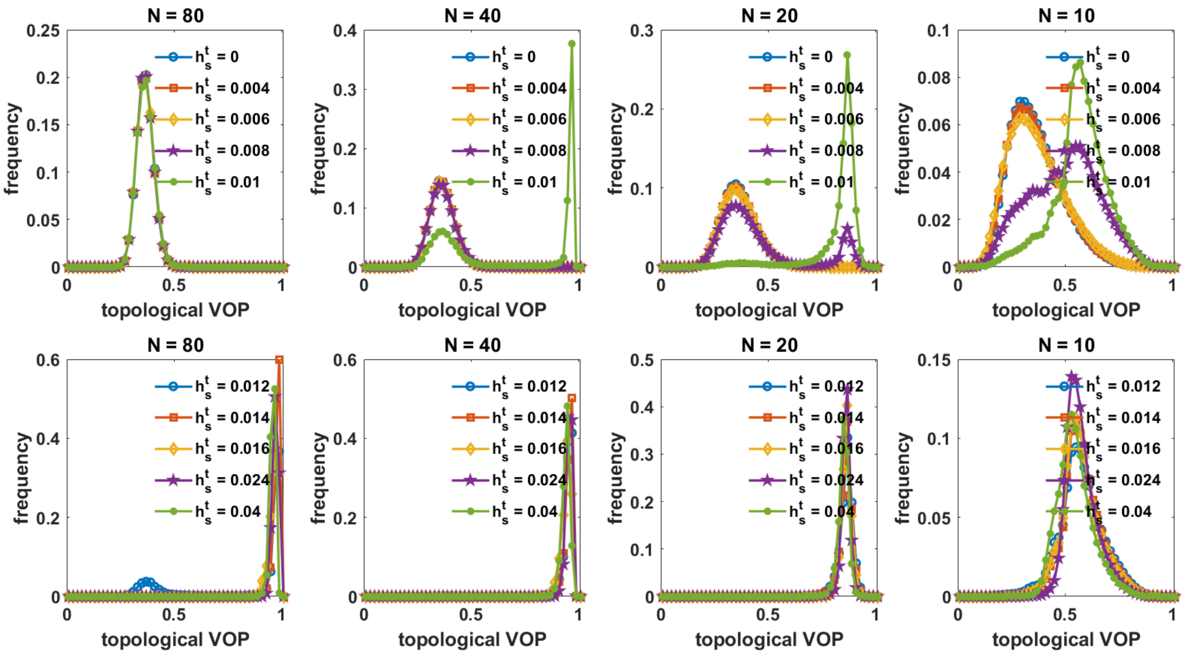

Supplementary Figure. 43: The distribution of Local Order (topological vectorial order parameter VOP) in groups of agents with an egocentric representation of space and in groups of various population sizes. Each panel shows groups of a given size shown on the panel. Total social attraction is shown in the legend. Each group size is broken into two panels to increase visibility. As social attraction increases, the system shows a discontinuous order-disorder phase transition. In the ordered phase, cohesive motion, but not collective motion is observed. In larger group sizes the transition becomes sharper. Parameter values: $N_s = 100$, $\sigma = 2\pi/N_s$, $\nu = 0.5$, $v_0 = 10$, and $\beta = 400$. Agents move in a periodic space with linear size, $L = 1000$.

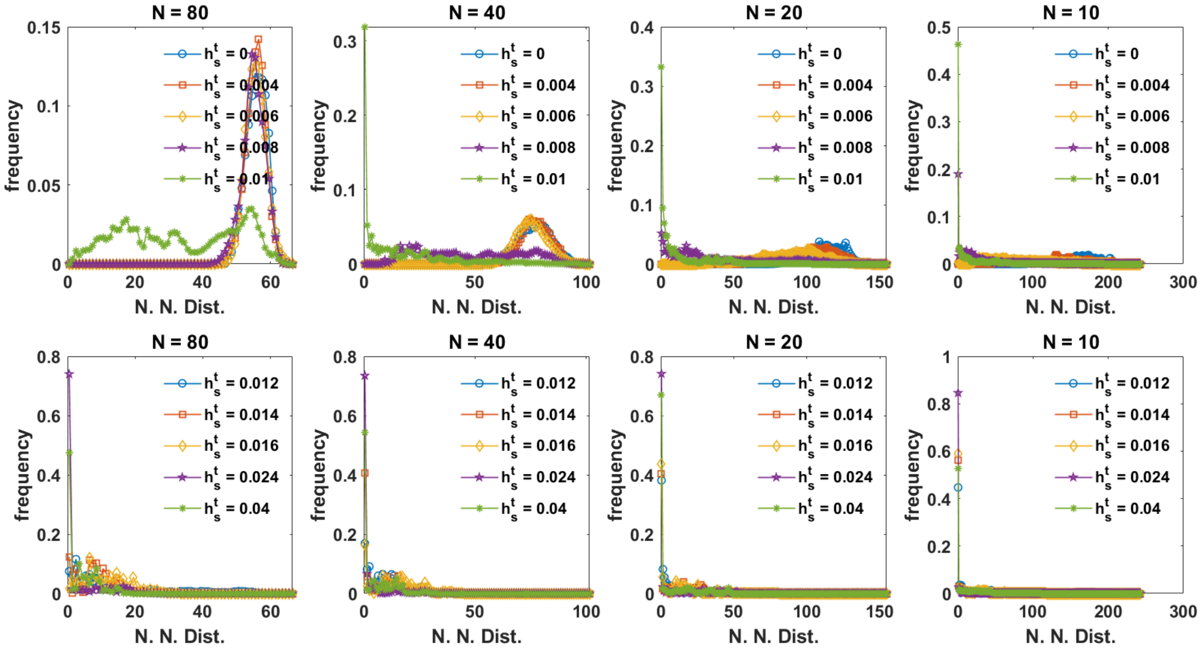

Supplementary Figure. 44: The distribution of mean nearest neighbor distance (N. N. Dist) in groups of agents with an egocentric representation of space and with various population sizes. Each panel shows groups of a given size shown on the panel. Total social attraction is shown in the legend. Each group size is broken into two panels to increase visibility. As social attraction increases, the system shows a discontinuous order-disorder phase transition. In the ordered phase, the distance between agents decreases. However, cohesive motion, but not collective motion is observed. In larger group sizes the transition becomes sharper. Parameter values: $N_s = 100$, $\sigma = 2\pi/N_s$, $\nu = 0.5$, $v_0 = 10$, and $\beta = 400$. Agents move in a periodic space with linear size, $L = 1000$.

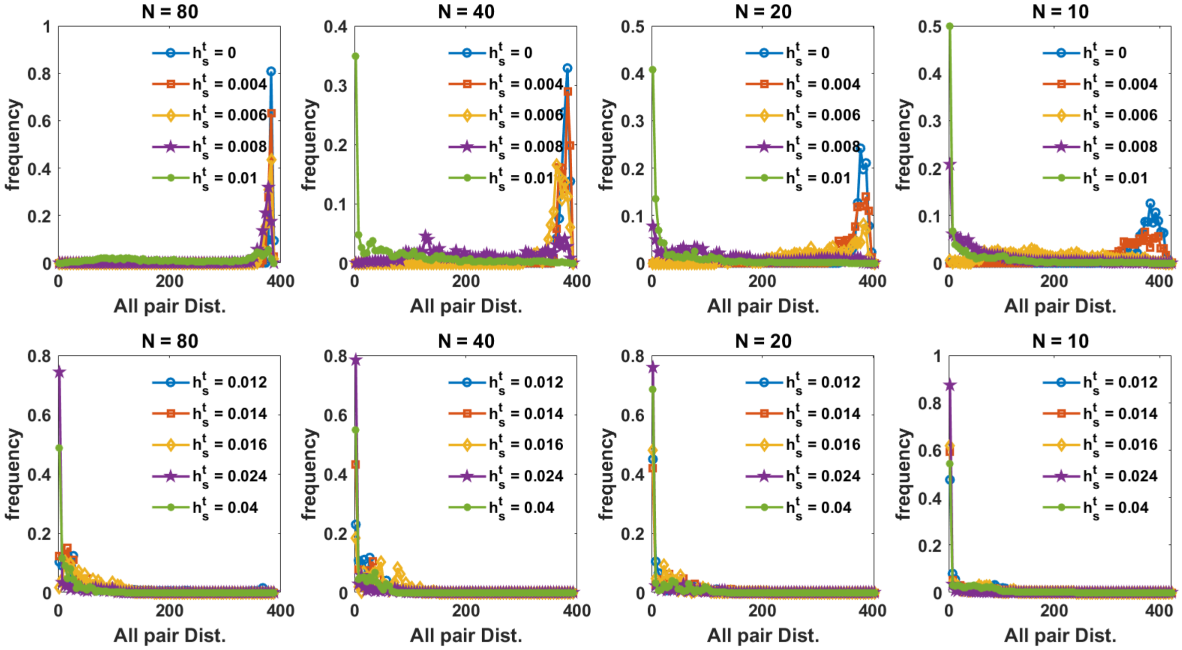

Supplementary Figure. 45: The distribution of mean nearest neighbor distance (N. N. Dist) in groups of agents with an egocentric representation of space and with various population sizes. Each panel shows groups of a given size shown on the panel. Total social attraction is shown in the legend. Each group size is broken into two panels to increase visibility. As social attraction increases, the system shows a discontinuous order-disorder phase transition. In the ordered phase, the distance between agents decreases and all the agents coalesce. However, cohesive motion is observed. In larger group sizes the transition becomes sharper. Parameter values: $N_s = 100$, $\sigma = 2\pi/N_s$, $\nu = 0.5$, $v_0 = 10$, and $\beta = 400$. Agents move in a periodic space with linear size, $L = 1000$.

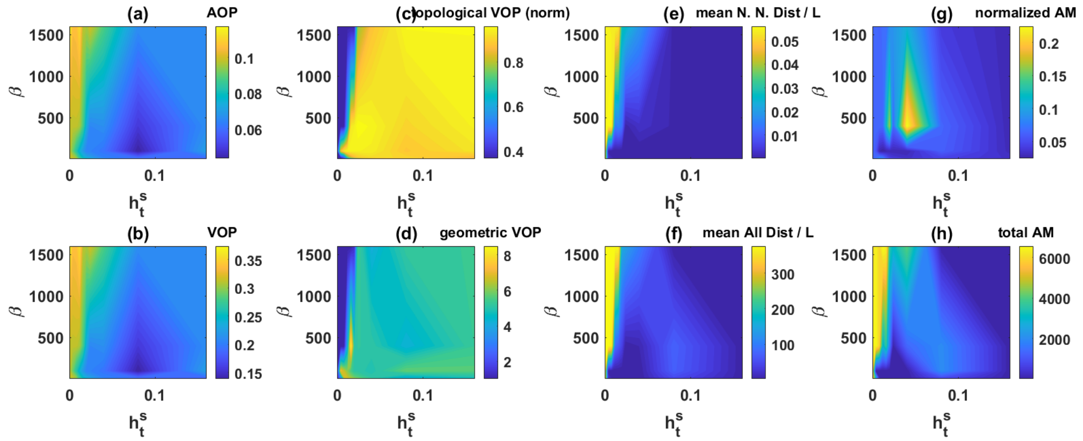

Supplementary Figure. 46: Contour plots of collective motion metrics in $\beta - h_t^s$ space in groups of 80 agents with an egocentric representation of space. The collective motion order parameter is color plotted as a function of the social attraction (h_s^0) and network inverse temperature (β). For too small social attraction, the agents move independently. As social attraction increases, collective motion is not observed. Rather, the system shows a phase transition to a phase where cohesive motion with low global order (global angular order parameter (AOP in (a)) and vectorial order parameter (VOP in (b))) and low mean distance between agents (mean nearest neighbor distance (e) and the distance between all pairs (f)), but high local order (topological VOP in (c) and geometric VOP in (d)) is observed. While the agents coalesce for high social attraction, the group of packed agents shows a slow collective motion for too high social attraction. normalized and total angular momentum (AM, (g) and (h) generally remain small. Parameter values: $N_s = 100$, $\sigma = 2\pi/N_s$, $v_0 = 10$. 80 agents move in a periodic space with linear size, $L = 1000$.

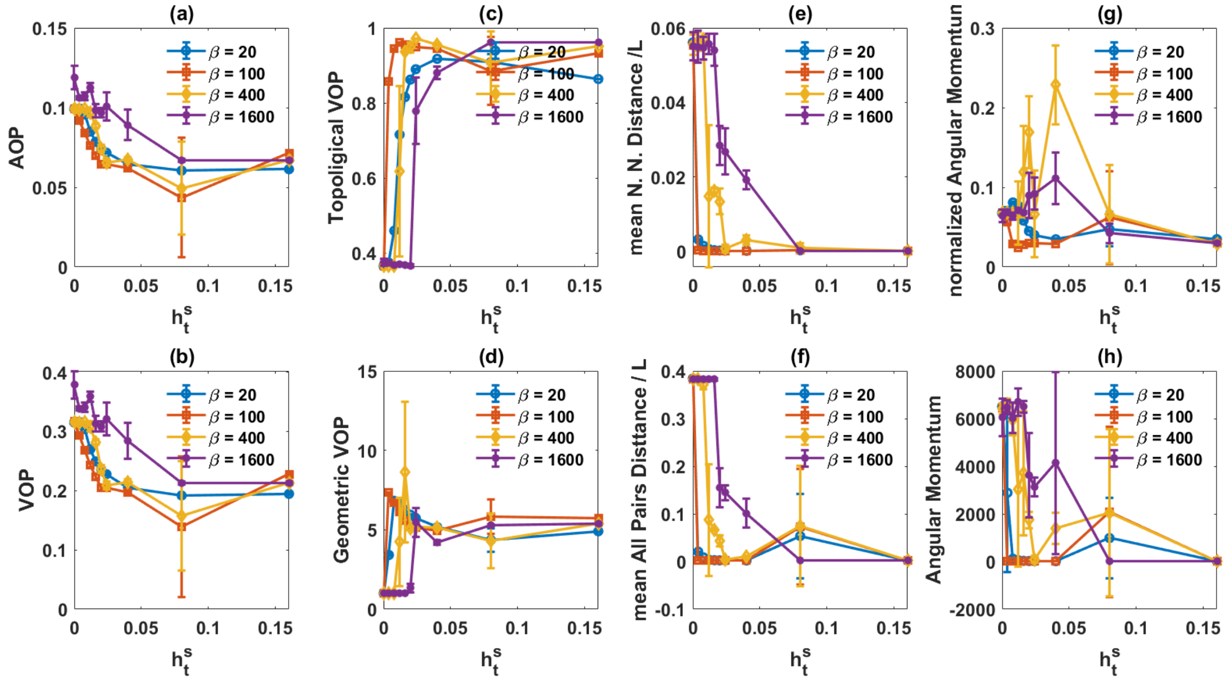

Supplementary Figure. 47: Measures of collective motion in groups of 80 agents with an egocentric representation of space. Different measures of collective motion of agents are shown as a function of the social attraction and for different values of β. For too small social attraction, the agents move independently. As social attraction increases, collective motion is not observed. Rather, the system shows a phase transition to a phase where cohesive motion with low global order (global angular order parameter (AOP in (a)) and vectorial order parameter (VOP in (b))) and low mean distance between agents (mean nearest neighbor distance (e) and the distance between all pairs (f)), but high local order (topological VOP in (c) and geometric VOP in (d)) is observed. While the agents coalesce for high social attraction, the group of packed agents show a slow collective motion for too high social attraction. normalized and total angular momentum (AM, (g) and (h) generally remain small. Parameter values: $N_s = 100$, $\sigma = 2\pi/N_s$, $\nu = 0.5$, $v_0 = 10$, and $\beta = 400$. 80 agents move in a periodic space with linear size, $L = 1000$.

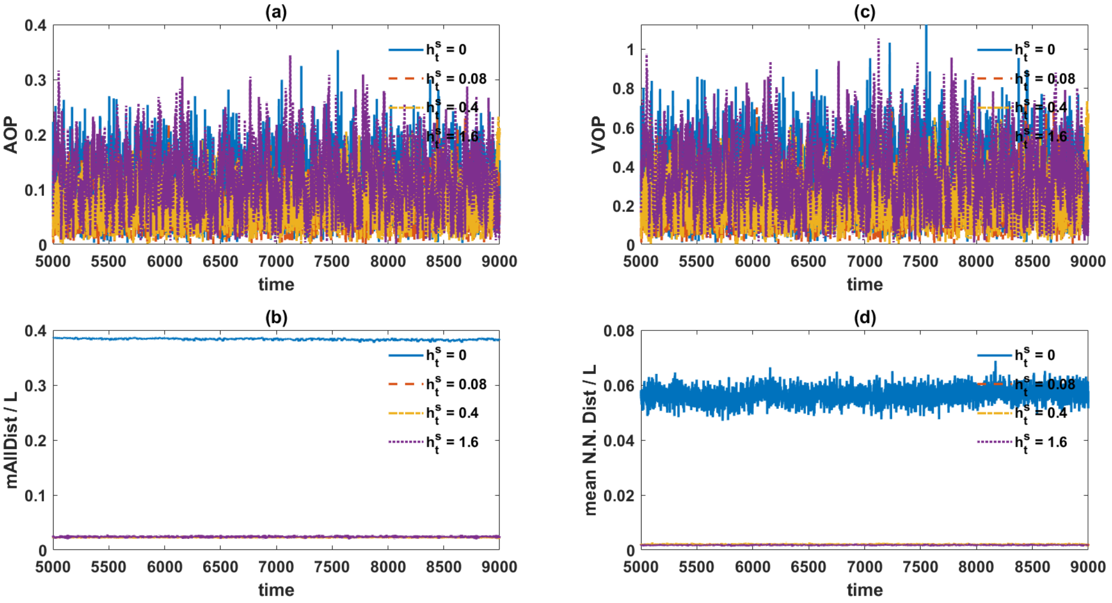

Supplementary Figure. 48: Time dependence of collective movement in groups of 80 agents with an egocentric representation of space. Global Order (Angular order parameter, AOP in (a)), the mean distance between all the pairs (mAllDist in (b)), vectorial order parameter (VOP, a measure of global order, in (c)), and mean nearest neighbor distance (N.N. Dist/L in (d)) for four different values of total social attraction indicated in the legend as a function of time is shown. With an egocentric representation of space, collective motion is not observed. Rather, for too low social attraction agents move freely and by increasing social attraction a transition to a phase where agents show cohesive motion leading to high local order but low global order is observed. Parameter values: $N = 80$. Parameter values: $N_s = 100$, $\sigma = 2\pi/N_s$, $\nu = 0.5$, $v_0 = 10$, and $\beta = 400$. Agents move in a periodic space with linear size, $L = 1000$.

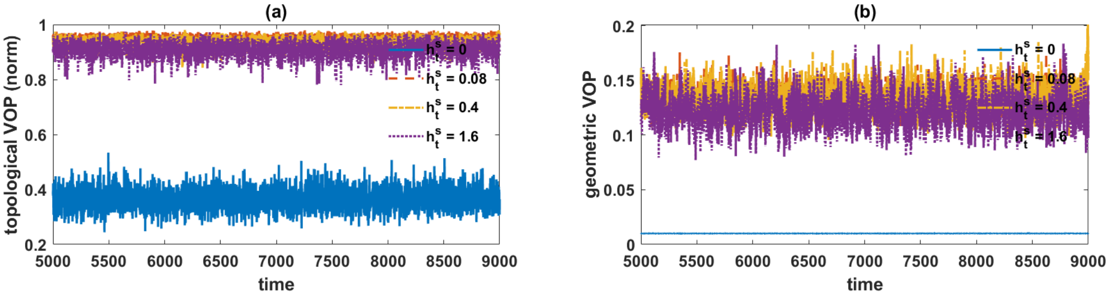

Supplementary Figure. 49: Time dependence of collective movement in groups of 80 agents with an egocentric representation of space. Two measures of local order, normalized topological VOP in (a), and the geometric VOP in (b), for different values of social attraction are plotted as a function of time. With an egocentric representation of space, collective motion is not observed. Rather, for too low social attraction agents move freely and by increasing social attraction a transition to a phase where agents show cohesive motion leading to high local order but low global order is observed. Parameter values: $N_s = 100$, $\sigma = 2\pi/N_s$, $\nu = 0.5$, $v_0 = 10$, and $\beta = 400$. 80 agents move in a periodic space with linear size, $L = 1000$.

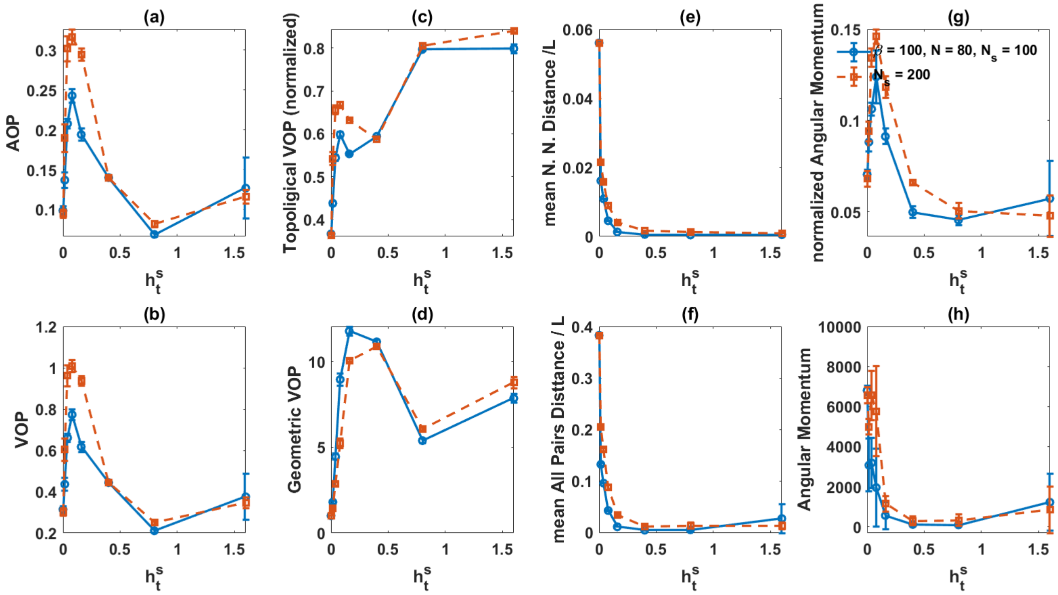

Supplementary Figure. 50: Different measures of collective motion in agents are shown as a function of social attraction and for two different numbers of spins for individuals' networks ($\beta = 100$). (a) to (h) show the global angular order parameter (AOP, (a)), vectorial order parameter (VOP, (b)), local VOP, both topological (c) and geometric (d), mean nearest neighbor distance (e), and all-pair distances (f), normalized and total angular momentum (AM, (g) and (h)). Parameter values: $N_s = 100$, $\sigma = 2\pi/N_s$, $\nu = 0.5$, $v_0 = 10$, and $\beta = 100$. 80 agents move in a periodic space with linear size, $L = 1000$.

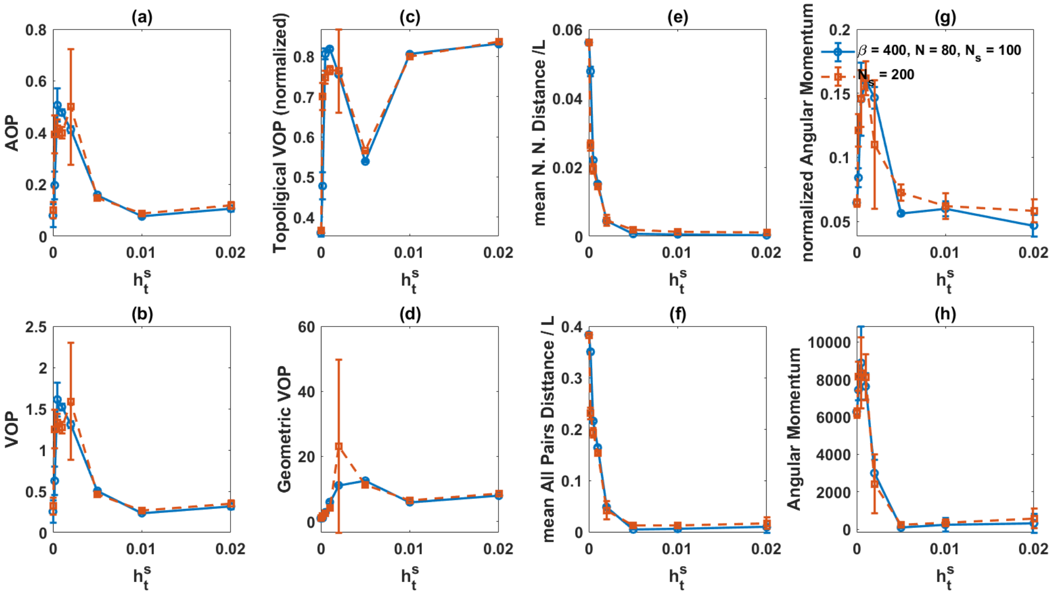

Supplementary Figure. 51: Different measures of collective motion of agents are shown as a function of social attraction and for two different numbers of spins for individuals' networks ($\beta = 400$). (a) to (h) show the global angular order parameter (AOP, (a)), vectorial order parameter (VOP, (b)), local VOP, both topological (c) and geometric (d), mean nearest neighbor distance (e), and all-pair distances (f), normalized and total angular momentum (AM, (g) and (h)). Parameter values: $\sigma = 2\pi/N_s$, $\nu = 0.5$, $v_0 = 10$, and $\beta = 400$. 80 agents move in a periodic space with linear size, $L = 1000$.

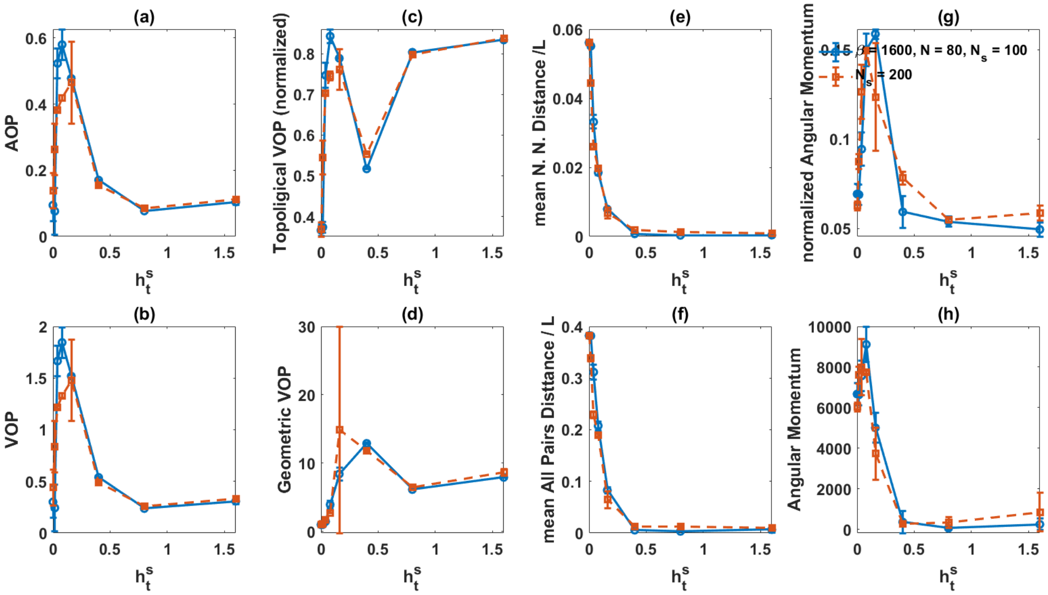

Supplementary Figure. 52: Different measures of collective motion of agents are shown as a function of social attraction and for two different numbers of spins for individuals' networks ($\beta = 1600$). (a) to (h) show the global angular order parameter (AOP, (a)), vectorial order parameter (VOP, (b)), local VOP, both topological (c) and geometric (d), mean nearest neighbor distance (e), and all-pair distances (f), normalized and total angular momentum (AM, (g) and (h)). Parameter values: Parameter values: $\sigma = 2\pi/N_s$, $\nu = 0.5$, $v_0 = 10$, and $\beta = 1600$. 80 agents move in a periodic space with linear size, $L = 1000$.

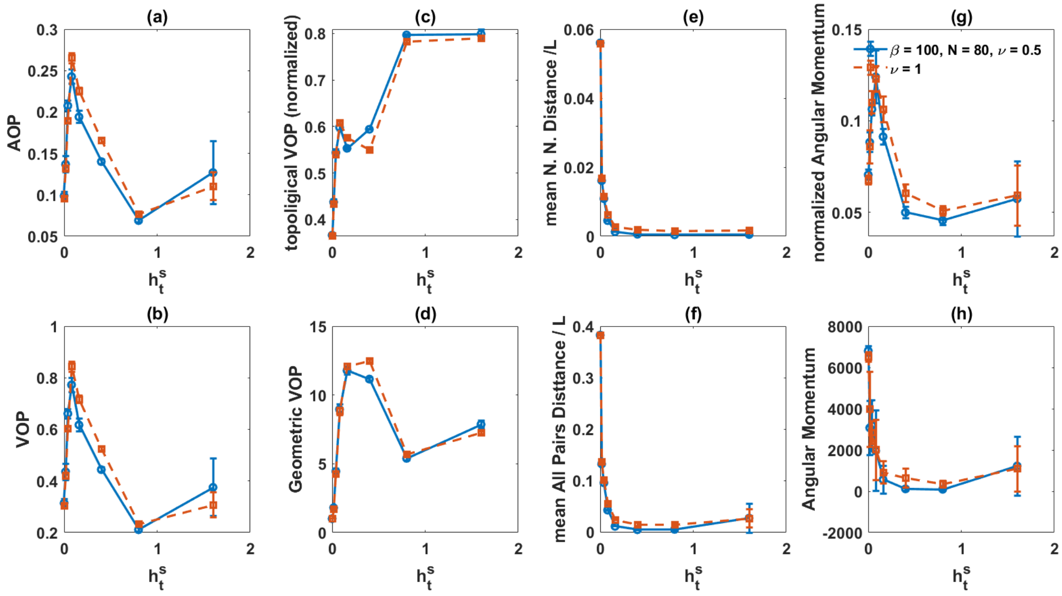

Supplementary Figure. 53: Different measures of collective motion of agents are shown as a function of social attraction and for two different values of the neural tuning parameter ($\beta = 100$). (a) to (h) show the global angular order parameter (AOP, (a)), vectorial order parameter (VOP, (b)), local VOP, both topological (c) and geometric (d), mean nearest neighbor distance (e), and all-pair distances (f), normalized and total angular momentum (AM, (g) and (h)). Parameter values: Parameter values: $N_s = 100$, $\sigma = 2\pi/N_s$, $v_0 = 10$, and $\beta = 100$. 80 agents move in a periodic space with linear size, $L = 1000$.

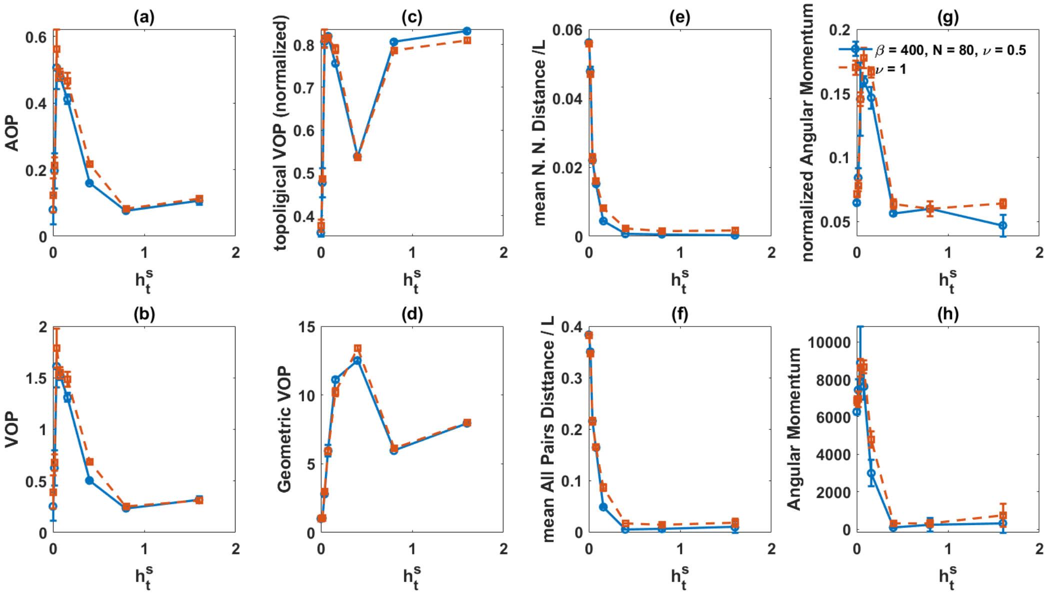

Supplementary Figure. 54: Different measures of collective motion of agents are shown as a function of social attraction and for two different values of the neural tuning parameter ($\beta = 400$). (a) to (h) show, the global angular order parameter (AOP, (a)), vectorial order parameter (VOP, (b)), local VOP, both topological (c) and geometric (d), mean nearest neighbor distance (e), and all-pair distances (f), normalized and total angular momentum (AM, (g) and (h)). Parameter values: Parameter values: $N_s = 100$, $\sigma = 2\pi/N_s$, $v_0 = 10$, and $\beta = 400$. 80 agents move in a periodic space with linear size, $L = 1000$.

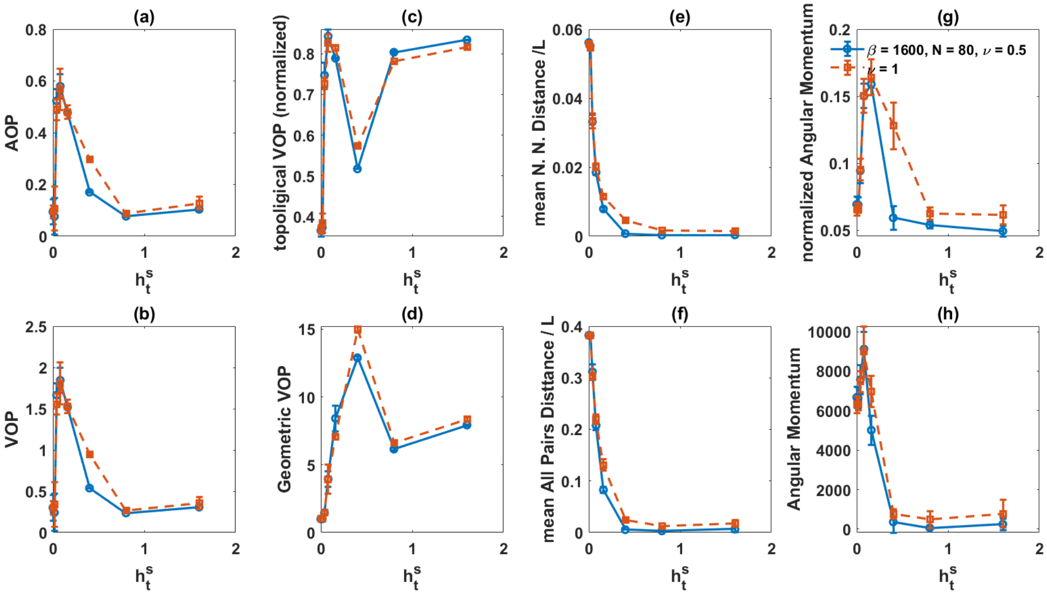

Supplementary Figure. 55: Different measures of collective motion of agents are shown as a function of social attraction and for two different values of the neural tuning parameter ($\beta = 1600$). (a) to (h) show the global angular order parameter (AOP, (a)), vectorial order parameter (VOP, (b)), local VOP, both topological (c) and geometric (d), mean nearest neighbor distance (e), and all-pair distances (f), normalized and total angular momentum (AM, (g) and (h)). Parameter values: Parameter values: $N_s = 100$, $\sigma = 2\pi/N_s$, $v_0 = 10$, and $\beta = 1600$. 80 agents move in a periodic space with linear size, $L = 1000$.

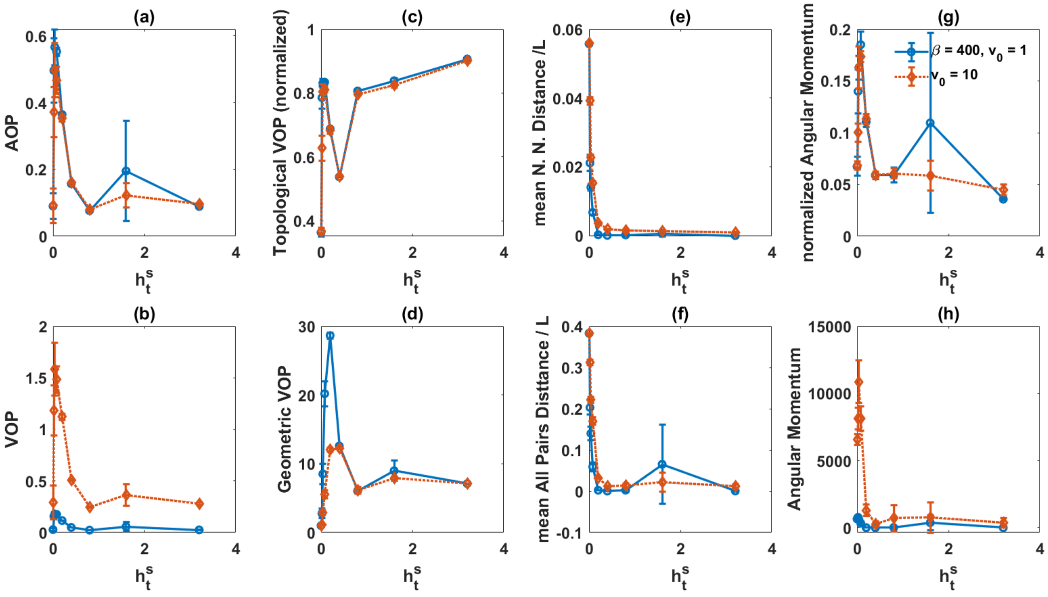

Supplementary Figure. 56: Different measures of collective motion of agents are shown as a function of social attraction and for two different values of agents' speed constant, v_0. (a) to (h) show the global angular order parameter (AOP, (a)), vectorial order parameter (VOP, (b)), local VOP, both topological (c) and geometric (d), mean nearest neighbor distance (e), and all-pair distances (f), normalized and total angular momentum (AM, (g) and (h)). $N_s = 100$, $\sigma = 2\pi/N_s$, $\nu = 0.5$, and $\beta = 400$. 80 agents move in a periodic space with linear size, $L = 1000$. v_0 is shown in the legend.

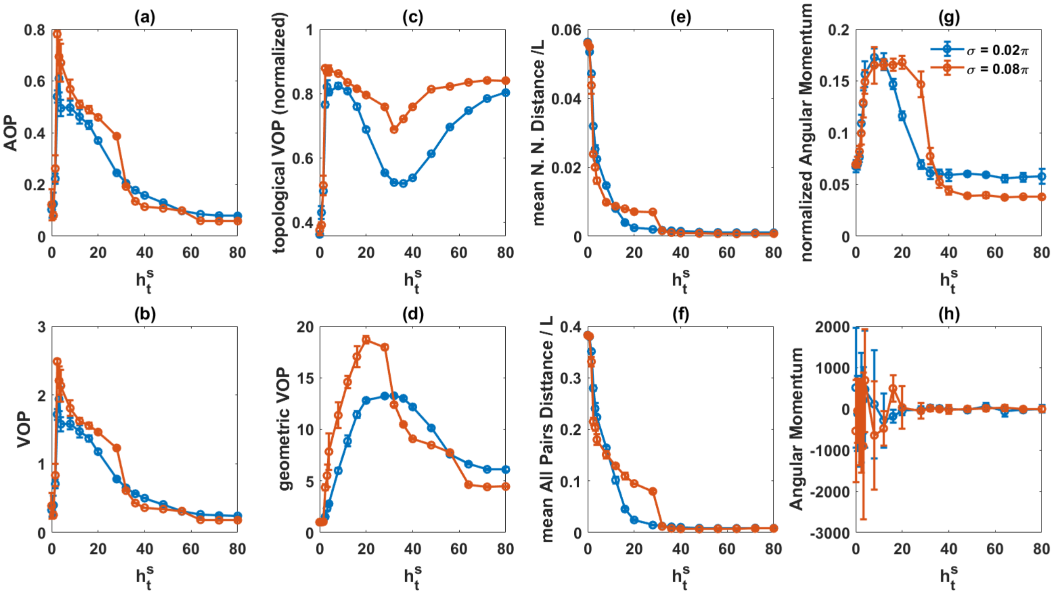

Supplementary Figure. 57: Different measures of collective motion of agents are shown as a function of social attraction and for two different values of agents' receptive field width, σ. (a) to (h) show, the global angular order parameter (AOP, (a)), vectorial order parameter (VOP, (b)), local VOP, both topological (c) and geometric (d), mean nearest neighbor distance (e), and all-pair distances (f), normalized and total angular momentum (AM, (g) and (h)). $N_s = 100$, $\nu = 0.5$, $v_0 = 10$, and $\beta = 400$. 80 agents move in a periodic space with linear size, $L = 1000$. σ is shown in the legend.

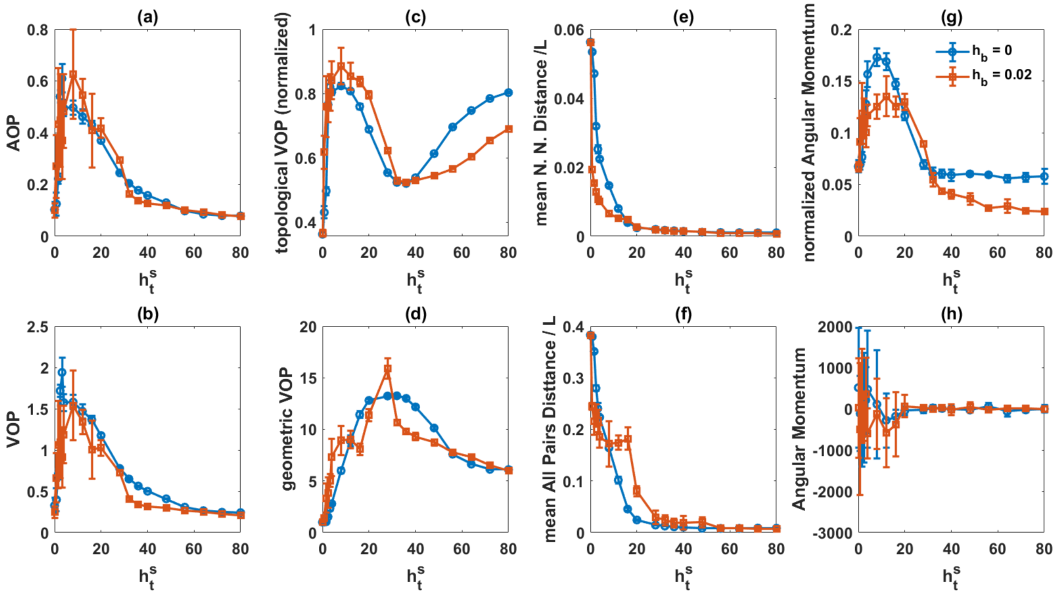

Supplementary Figure. 58: Different measures of collective motion of agents are shown as a function of social attraction and for two different values of base field, h_b. (a) to (h) show the global angular order parameter (AOP, (a)), vectorial order parameter (VOP, (b)), local VOP, both topological (c) and geometric (d), mean nearest neighbor distance (e), and all-pair distances (f), normalized and total angular momentum (AM, (g) and (h)). $N_s = 100$, $\sigma = 2\pi/N_s$, $\nu = 0.5$, $v_0 = 10$, and $\beta = 400$. 80 agents move in a periodic space with linear size, $L = 1000$.

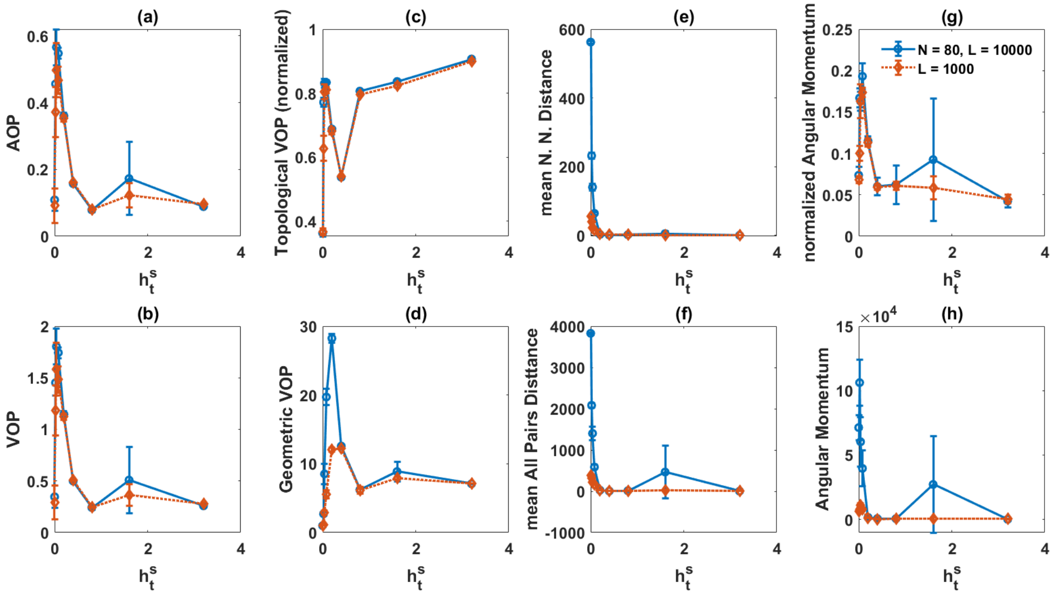

Supplementary Figure. 59: Different measures of collective motion of agents are shown as a function of social attraction and for two different densities. (a) to (h) show the global angular order parameter (AOP, (a)), vectorial order parameter (VOP, (b)), local VOP, both topological (c) and geometric (d), mean nearest neighbor distance (e), and all-pair distances (f), normalized and total angular momentum (AM, (g) and (h)). $N_s = 100$, $\sigma = 2\pi/N_s$, $\nu = 0.5$, $v_0 = 10$, and $\beta = 400$. 80 agents move in a periodic space with linear size, $L = 1000$ and $L = 10000$.

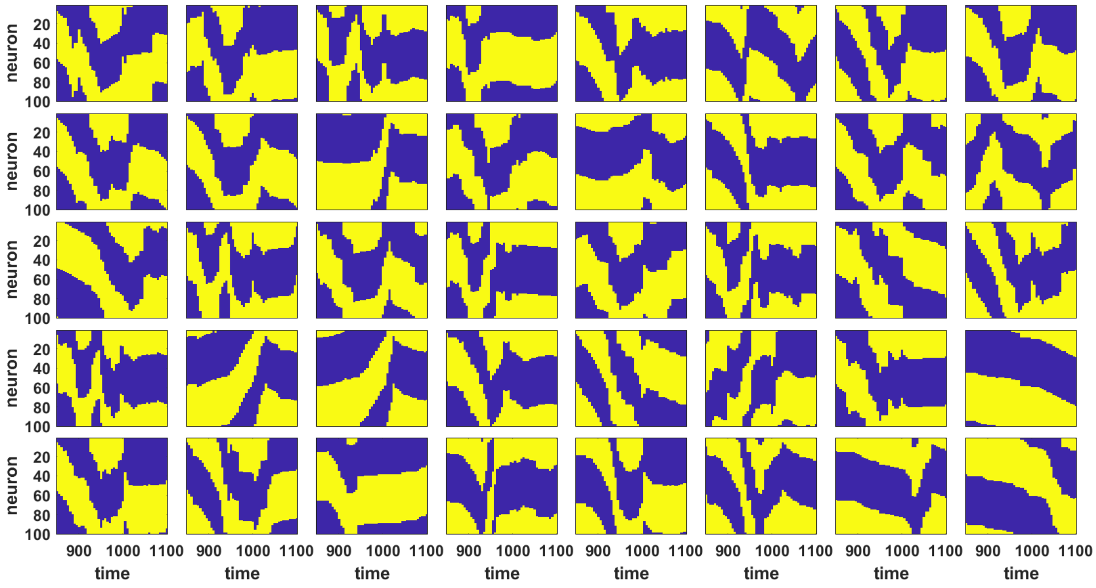

Supplementary Figure. 60: The neural activity of 40 agents during collective movement in a population of 80 agents is shown. The bright color shows active spins (neuron groups) and the dark color shows inactive neurons. The trajectory of the agents is shown in Supplementary Figure. 62. The population of agents can be decomposed into subgroups of agents whose neural dynamics are synchronized and move together. During the fission-fusion dynamics, individuals change their group, groups split, or new groups are formed. Consequently, subgroups of synchronized agents change. As a result Parameter values: $N_s = 100$, $\sigma = 2\pi/N_s$, $\nu = 0.5$, $v_0 = 10$, and $\beta = 400$. 80 agents move in a periodic space with linear size $L = 1000$.

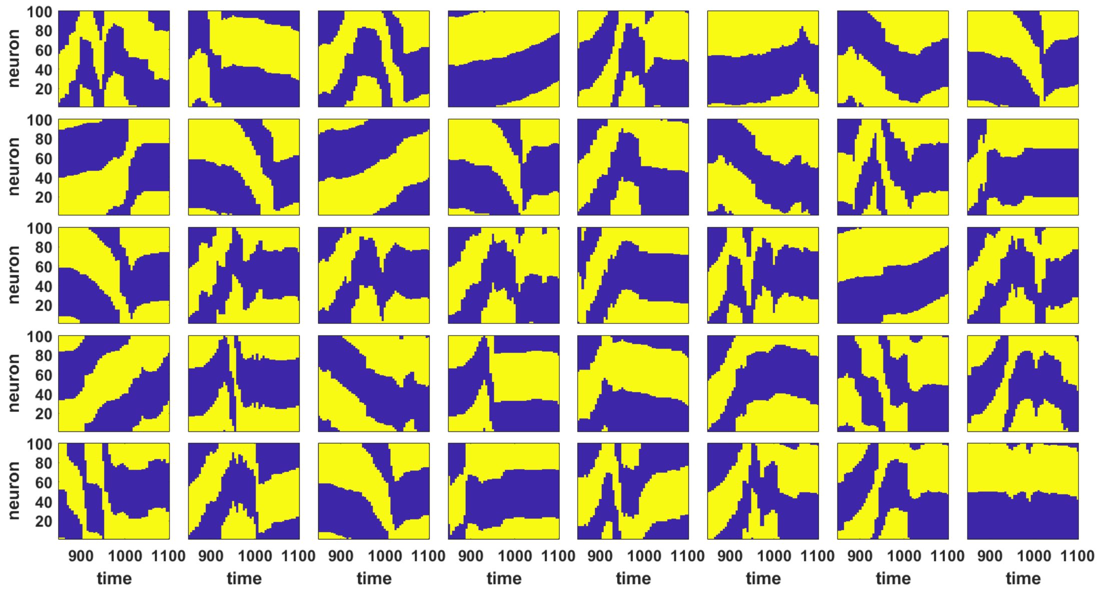

Supplementary Figure. 61: The neural activity of 40 agents during collective movement in a population of 80 agents is shown. The bright color shows active spins (neuron groups) and the dark color shows inactive neurons. The trajectory of the agents is shown in Supplementary Figure. 62. The population of agents can be decomposed into subgroups of agents whose neural dynamics are synchronized and move together. During the fission-fusion dynamics, individuals change their group, groups split, or new groups are formed. Consequently, subgroups of synchronized agents change. As a result Parameter values: $N_s = 100$, $\sigma = 2\pi/N_s$, $\nu = 0.5$, $v_0 = 10$, and $\beta = 400$. 80 agents move in a periodic space with linear size $L = 1000$.

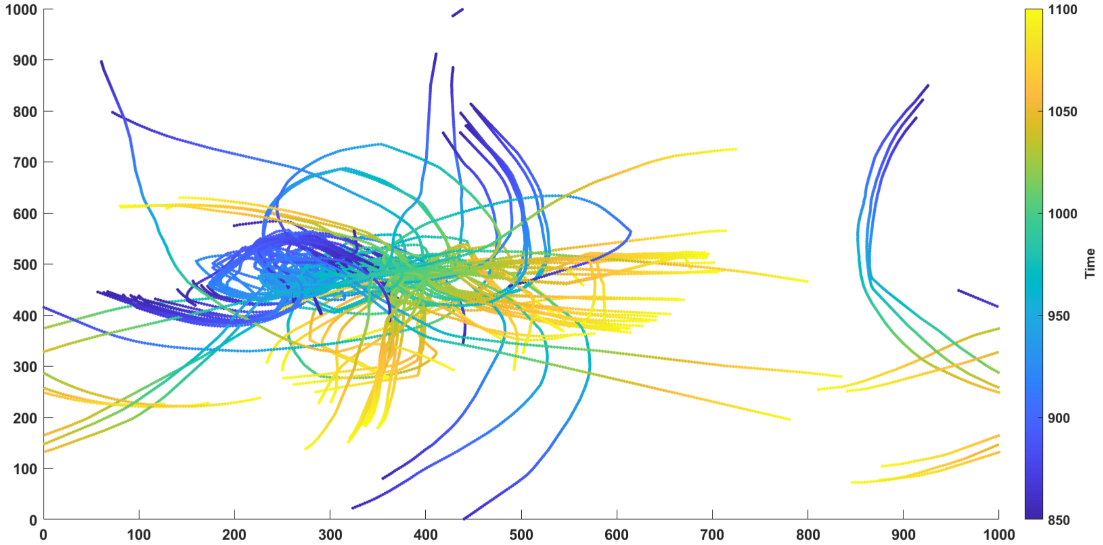

Supplementary Figure. 62: The trajectory of 80 collectively moving agents are shown. Time is color-coded. Parameter values: $N_s = 100$, $\sigma = 2\pi/N_s$, $\nu = 0.5$, $v_0 = 10$, and $\beta = 400$. 80 agents move in a periodic space (thus, agents reappear from the opposite side of the arena, when they reach a boundary) with linear size $L = 1000$.

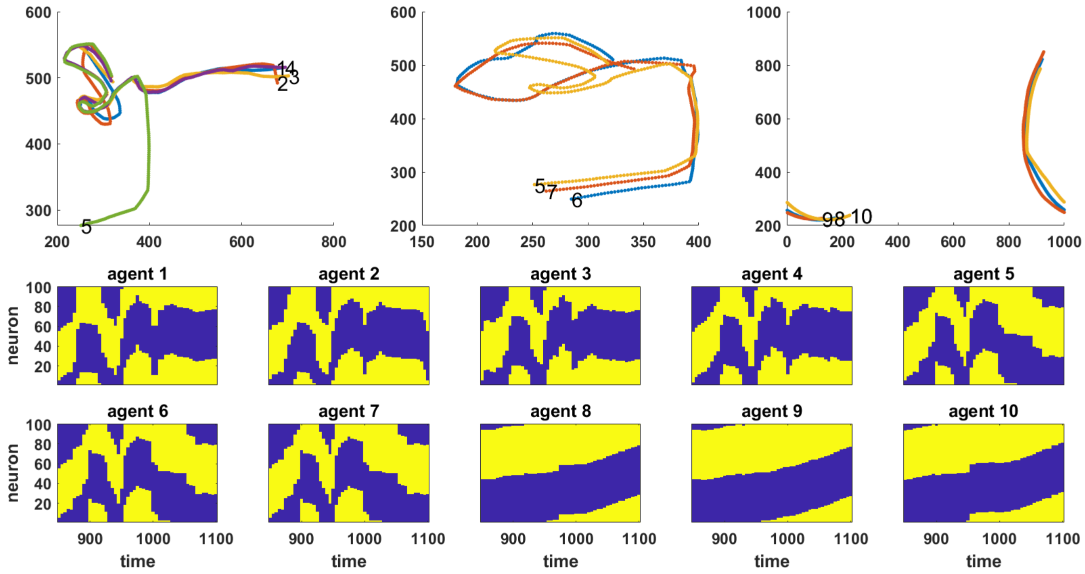

Supplementary Figure. 63: Synchronization and fission-fusion dynamics in collective movement. The trajectories of 10 agents broken into synchronized groups in a population of 80 agents are shown (top panels). The trajectory of all the agents is shown in Supplementary Figure. 63. The neural activities of the agents are shown in the bottom panels. Among the 80 agents in the population shown in Supplementary Figure. 60 and Supplementary Figure. 61, three groups with similar neural dynamics are shown separately. Agent 5 changes its group around time 1000. While agents 1 to 7 show similar neural dynamics and trajectories up to time 1000, their neural dynamics diverges after this time leading to the split of the group into two different groups. Agent 5 is better synchronized with agents 3 and 4 before this time and become synchronized with agents 6 and 7 after this time.

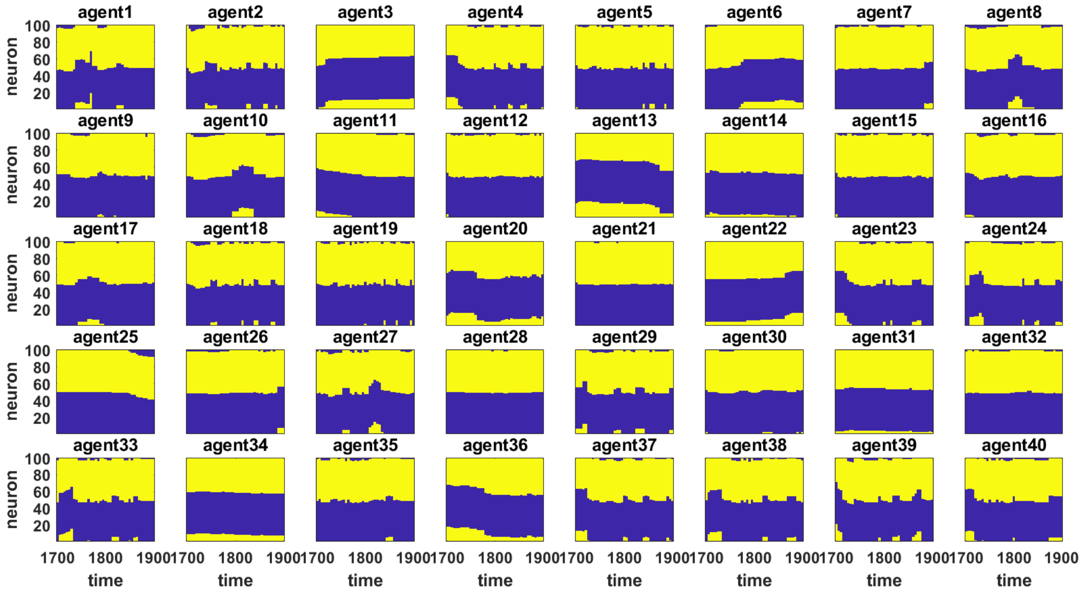

Supplementary Figure. 64: The neural activity of 40 agents during collective movement in a population of 80 agents is shown. The bright color shows active neurons and the dark color shows inactive neurons. The trajectory of the agents is shown in Supplementary Figure. 62. The population of agents can be decomposed into subgroups of agents whose neural dynamics are synchronized and move together. During the fission-fusion dynamics, individuals change their group, groups split, or new groups are formed. Consequently, subgroups of synchronized agents change. As a result Parameter values: $N_s = 100$, $\sigma = 2\pi/N_s$, $\nu = 0.5$, $v_0 = 10$, and $\beta = 400$. 80 agents move in a periodic space with linear size $L = 1000$.

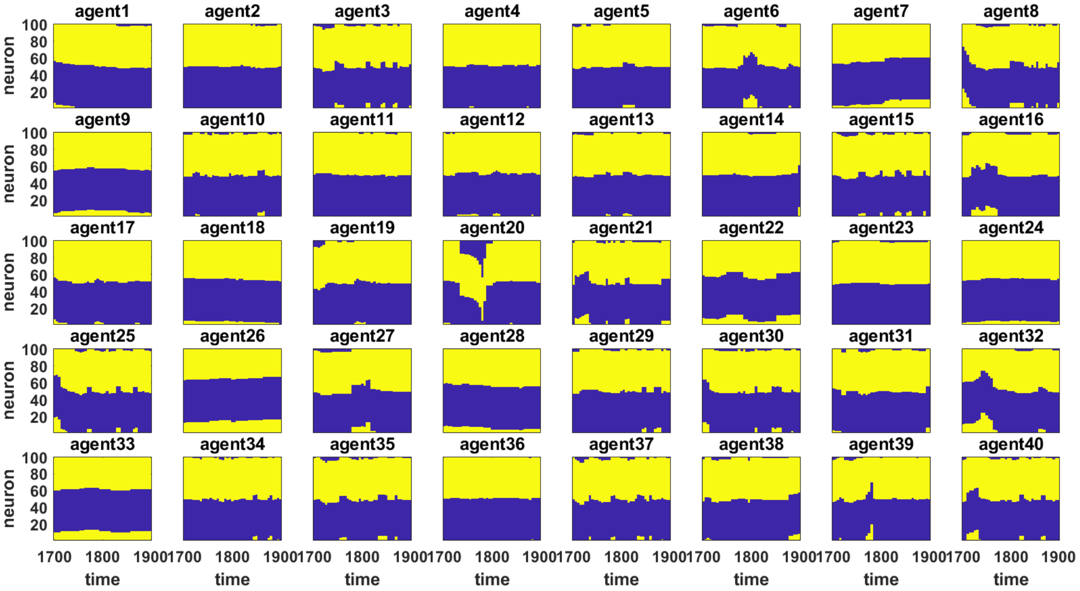

Supplementary Figure. 65: The neural activity of 40 agents during collective movement in a population of 80 agents is shown. The bright color shows active neurons and the dark color shows inactive neurons. The trajectory of the agents is shown in Supplementary Figure. 62. The population of agents can be decomposed into subgroups of agents whose neural dynamics are synchronized and move together. During the fission-fusion dynamics, individuals change their group, groups split, or new groups are formed. Consequently, subgroups of synchronized agents change. As a result Parameter values: $N_s = 100$, $\sigma = 2\pi/N_s$, $\nu = 0.5$, $v_0 = 10$, and $\beta = 400$. 80 agents move in a periodic space with linear size $L = 1000$.

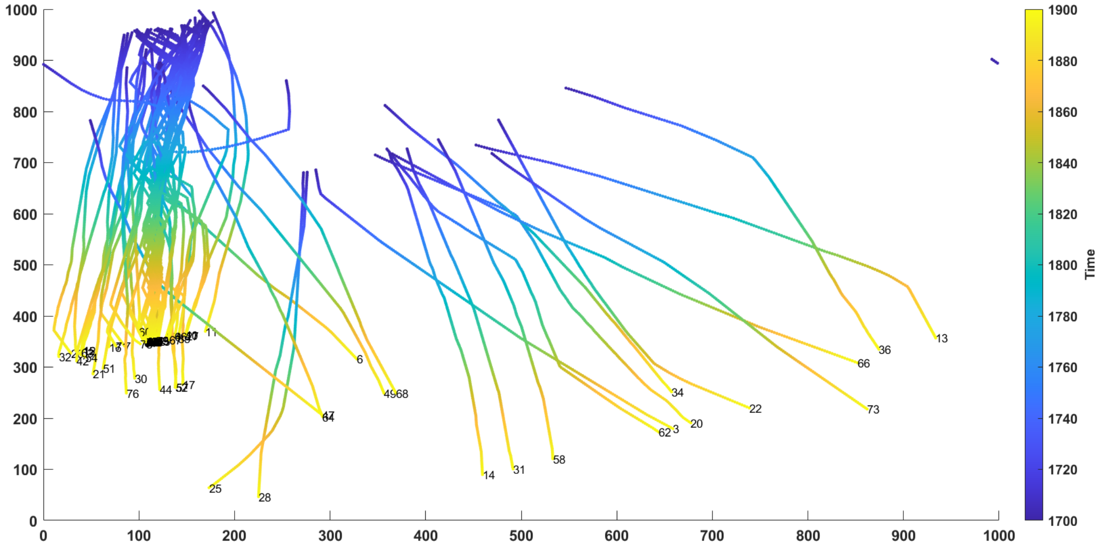

Supplementary Figure. 66: The trajectory of 80 collectively moving agents are shown. Time is color coded. Parameter values: $N_s = 100$, $\sigma = 2\pi/N_s$, $\nu = 0.5$, $v_0 = 10$, and $\beta = 400$. 80 agents move in a periodic space with linear size $L = 1000$.

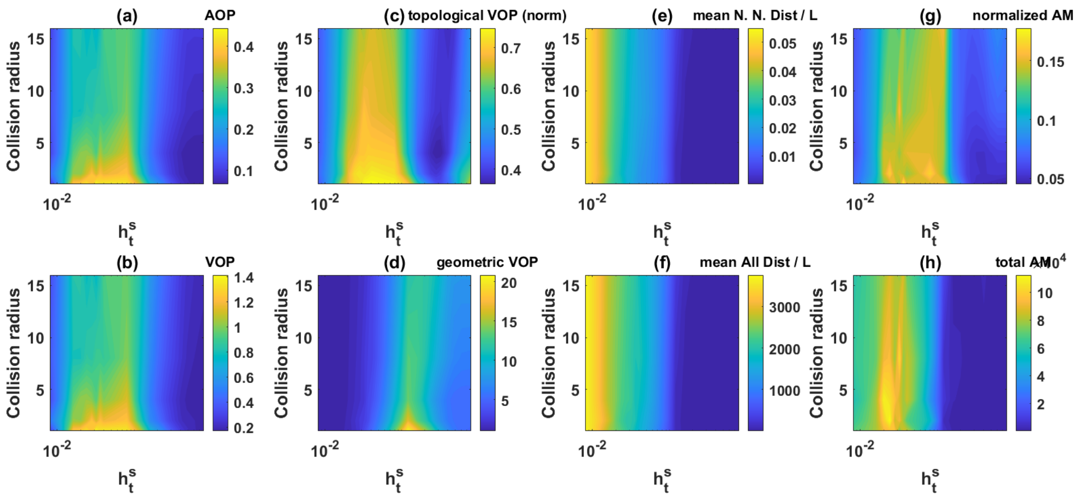

Supplementary Figure. 67: Contour plots of collective motion metrics with long-range attraction and short-range repulsion. Collective motion metrics are presented in the plane defined by total social attraction, h_t^s, and collision radius. The collective motion order parameter is color plotted as a function of the total social attraction (h_s^t) and collision radius, below which agents avoid collision. At zero social attraction, agents exhibit independent movement. An increase in h_s^t triggers a phase transition to ordered motion. Further increasing the social attraction results in agent coalescence and cohesive motion. Similar phenomenology is observed for non-zero collision radius. (a) to (h) show the global angular order parameter (AOP, (a)), vectorial order parameter (VOP, (b)), local VOP, both topological (c) and geometric (d), mean nearest neighbor distance (e), and all-pair distances (f), normalized and total angular momentum (AM, (g) and (h)). Parameter values: $N_s = 100$, $\sigma = 2\pi/N_s$, $v_0 = 10$, $\beta = 400$. $N = 80$ agents move in a periodic space with linear size, $L = 10000$. The strength of the external field below the collision radius is equal to -0.1.

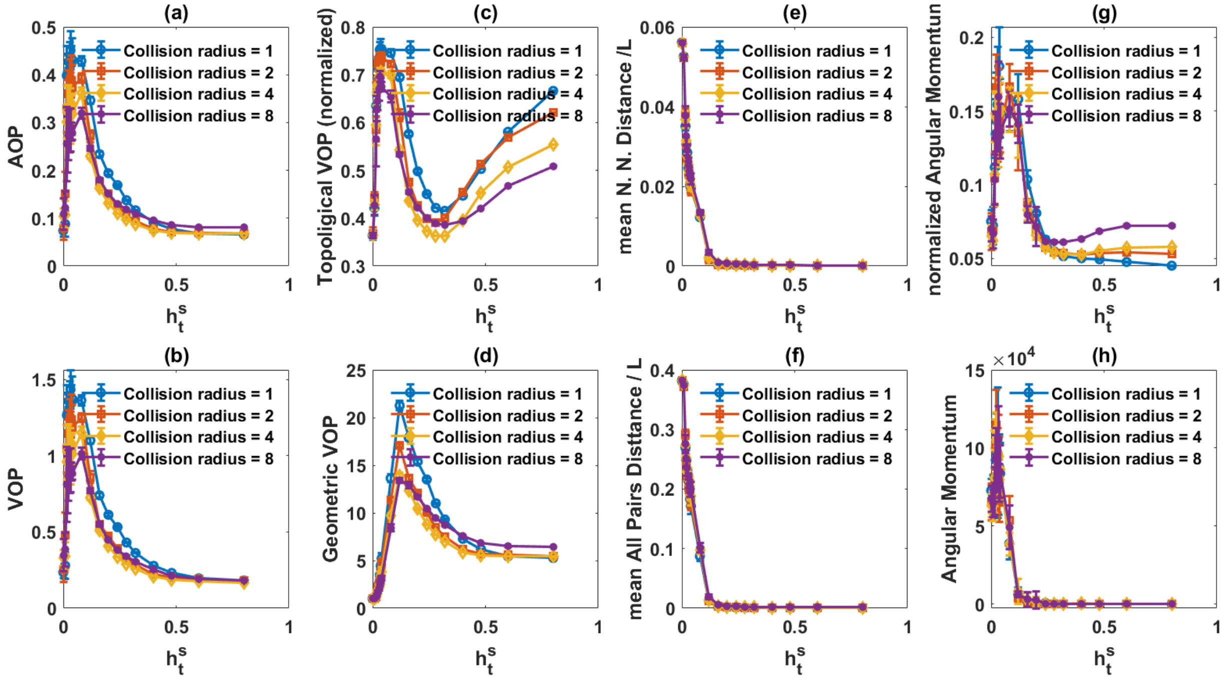

Supplementary Figure. 68: Measures of collective motion with long-range attraction and short-range repulsion. Different measures of collective motion of agents are shown as a function of the total social attraction, h_s^t, and for different collision radius, below which agents avoid collision are plotted. For zero social attraction, the agents move independently. As the social attraction increases, the system shows a phase transition to a phase where ordered motion is observed. Consequently, the global angular order parameter (AOP in (a)) and vectorial order parameter (VOP in (b)), as well as their local counterparts (topological VOP in (c) and geometric VOP in (d)) increase. By increasing the social attraction all the individuals coalesce without net transportative motion. In this phase, the mean nearest neighbor distance (e) and the distance between all pairs (f), as well as the order parameters are minimized. The normalized and total angular momentum (AM, in (g) and (h), respectively) is maximized in the ordered phase, due to the fission-fusion dynamics. Parameter values: $N = 320$. Parameter values: $N_s = 100$, $\sigma = 2\pi/N_s$, $v_0 = 10$. $N = 80$ agents move in a periodic space with linear size, $L = 10000$. The strength of the external field below the collision radius is equal to -0.1.

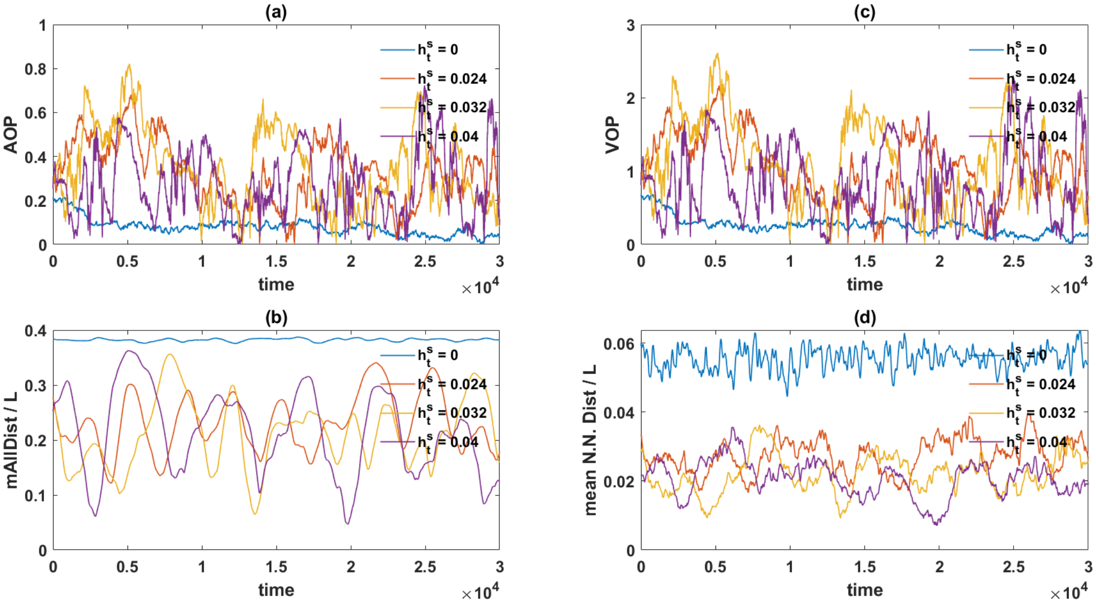

Supplementary Figure. 69: Time dependence of collective movement with short-range repulsion and long-range attraction. Angular order parameter (AOP in (a)), the mean distance between all the pairs (mAllDist in (b)), vectorial order parameter (VOP in (c)), and mean nearest neighbor distance (N. N. Dist/L in (d)) for four different values of total social attraction indicated in the legend as a function of time is shown. In the absence of social attraction, individuals do not interact and no collective motion is observed. For average social attraction, collective motion is observed. The intermittency indicates the strong fission-fusion dynamics in the system observed in large groups. For larger values of social attraction (not shown), cohesive motion with low global order and low distance between individuals is observed. Parameter values: $N_s = 100$, $\sigma = 2\pi/N_s$, $\nu = 0.5$, $v_0 = 10$, and $\beta = 400$. $N = 80$ agents move in a periodic space with linear size, $L = 10000$. The collision radius is set equal to 4. The strength of the external field below the collision radius is equal to -0.1.

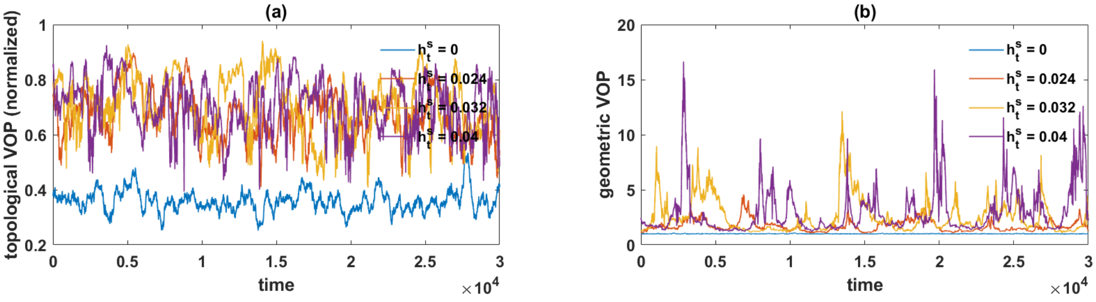

Supplementary Figure. 70: Time dependence of collective movement with short-range repulsion and long-range attraction. normalized topological vectorial order parameter (VOP in (a)), and the geometric VOP (b) for different values of social attraction are plotted as a function of time. Parameter values: $N_s = 100$, $\sigma = 2\pi/N_s$, $\nu = 0.5$, $v_0 = 10$, and $\beta = 400$. $N = 80$ agents move in a periodic space with linear size, $L = 10000$. The collision radius is set equal to 4. The strength of the external field below the collision radius is equal to -0.1. Geometric VOP is calculated as the order parameter between neighbors with a distance smaller than $L/100$.

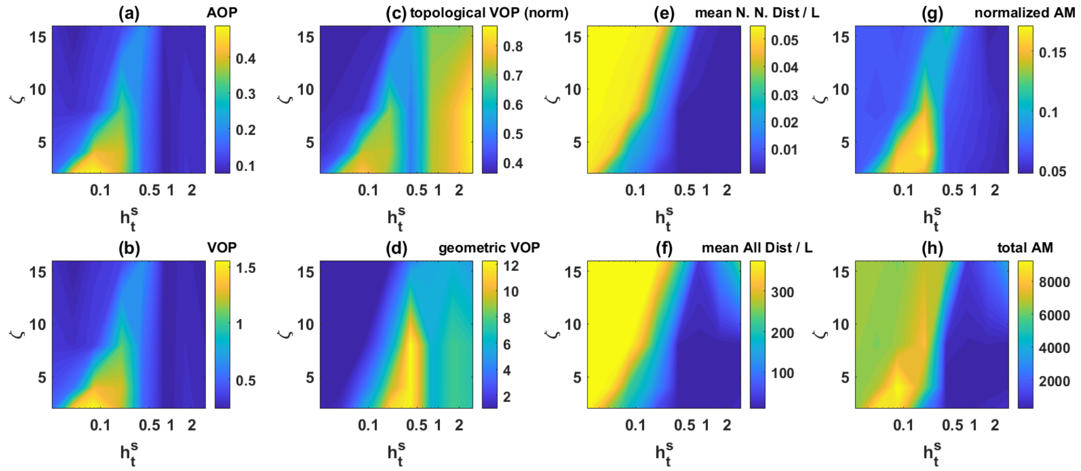

Supplementary Figure. 71: Contour plots of collective motion metrics with distance-dependent social attraction. Collective motion metrics are presented in the plane defined by total social attraction, h_t^s, and ζ, when social attraction decays exponentially with distance. Here, the magnitude of the social field, h_0^s, decays exponentially with distance according to, $h_0^s \exp(-d/(L/\zeta))$. The collective motion order parameter is color plotted as a function of the total social attraction (h_s^t) and ζ. By increasing h_s^t a phase transition to ordered motion is observed. Further increasing the social attraction results in agent coalescence and cohesive motion. Increasing ζ, leading to more short-range social attraction decreases global order. (a) to (h) show the global angular order parameter (AOP, (a)), vectorial order parameter (VOP, (b)), local VOP, both topological (c) and geometric (d), mean nearest neighbor distance (e), and all-pair distances (f), normalized and total angular momentum (AM, (g) and (h)). Parameter values: $N_s = 100$, $\sigma = 2\pi/N_s$, $v_0 = 10$, $\beta = 400$. $N = 80$ agents move in a periodic space with linear size, $L = 1000$.

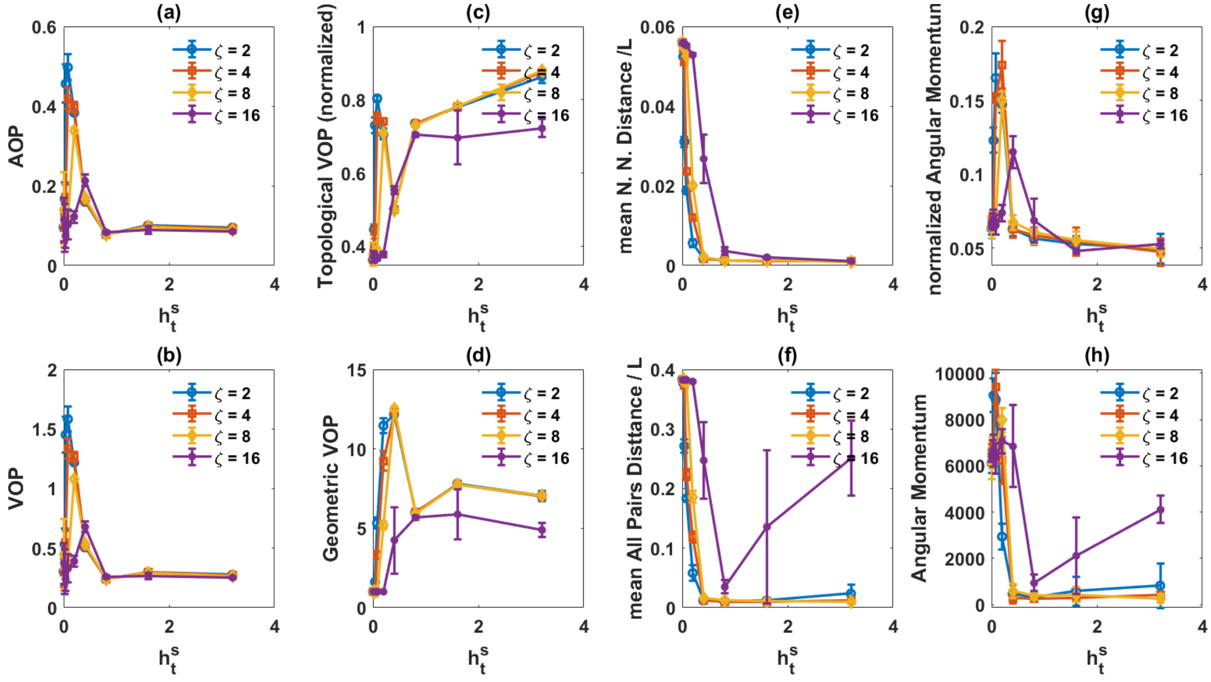

Supplementary Figure. 72: Measures of collective motion with distance-dependent social attraction. Different measures of collective motion of agents are shown as a function of the total social attraction, h_s^t, and different values of ζ are shown. Here, the magnitude of the social field, h_0^s, decays exponentially with distance according to, $h_0^s \exp(-d/(L/\zeta))$. For zero social attraction, the agents move independently. As the social attraction increases, the system shows a phase transition to a phase where ordered motion is observed. Consequently, the global angular order parameter (AOP in (a)) and vectorial order parameter (VOP in (b)), as well as their local counterparts (topological VOP in (c) and geometric VOP in (d)) increase. By increasing the social attraction all the individuals coalesce without net transportative motion. In this phase, the mean nearest neighbor distance (e) and the distance between all pairs (f), as well as the order parameters are minimized. The normalized and total angular momentum (AM, in (g) and (h), respectively) is maximized in the ordered phase, due to the fission-fusion dynamics. A comparison of the order parameters for different values of ζ shows that local and global order decreases when the range of social attraction decreases. Parameter values: $N = 320$. Parameter values: $N_s = 100$, $\sigma = 2\pi/N_s$, $v_0 = 10$. $N = 80$ agents move in a periodic space with linear size, $L = 1000$.

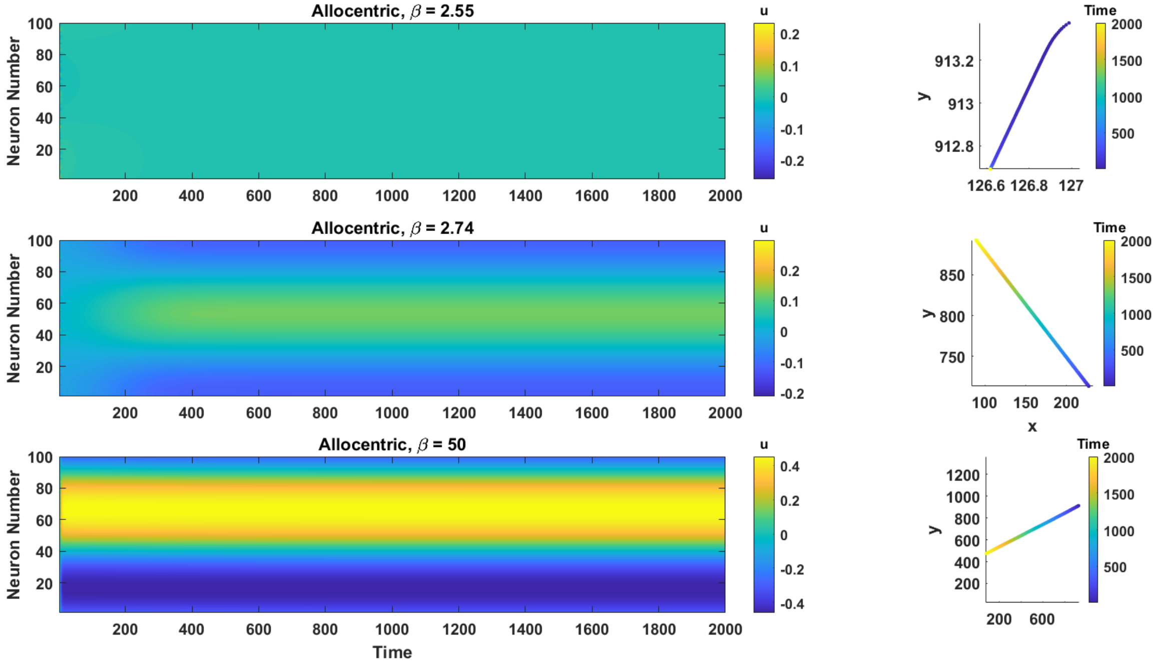

Supplementary Figure. 73: Movement patterns with an allocentric representation of space and during free motion in the neural field model. The network activity as a function of time (left) and the resulting trajectories (right) for different values of β, indicated on each panel are shown. The agent possesses an allocentric representation of space. Starting from a random initial condition for neuron potentials, u_i, for smaller values of β, after a short transient period, the agent stops moving. As β increases, a bump of activity appears in the network, leading to a motion along a directed trajectory. As β increases, the agent's speed increases and it saturates for large β. Parameter values: $N_s = 100$, $\sigma = 0.4$, $v_0 = 0.05$, $dt = 0.3$, $h_b = 0$. The agent moves in a periodic space with linear size, $L = 1000$.

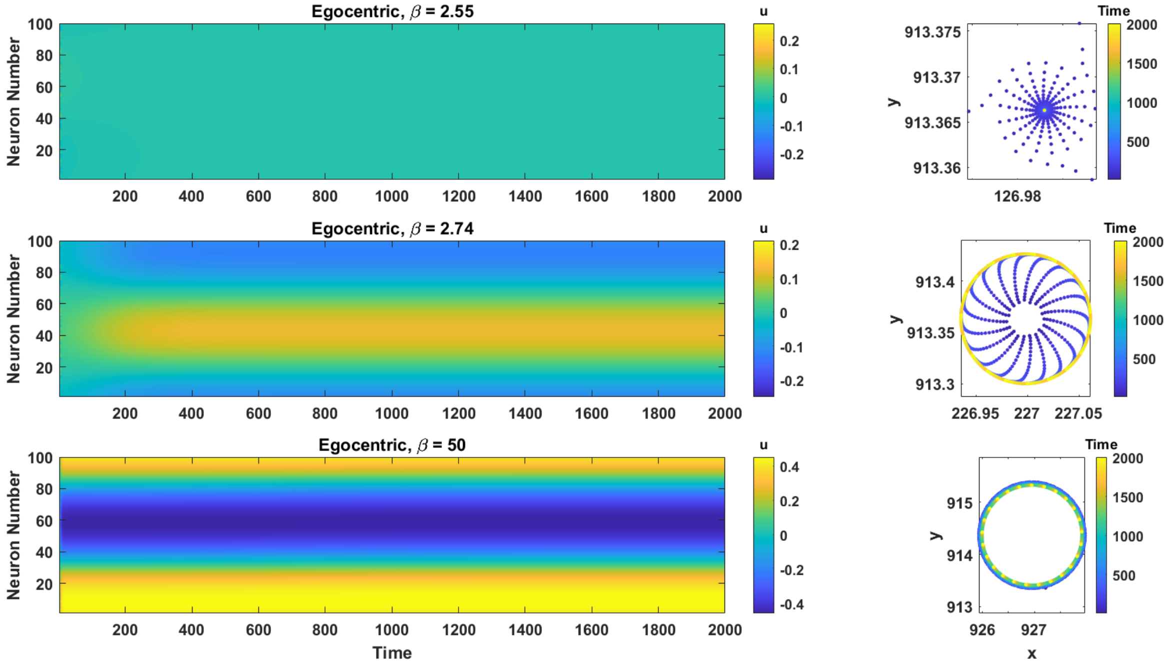

Supplementary Figure. 74: Movement patterns with an egocentric representation of space and during free motion in the neural field model. The network activity as a function of time (left) and the resulting trajectories (right) for different values of β, indicated on each panel are shown. The agent possesses an egocentric representation of space. Starting from a random initial condition for neuron potentials, u_i, for smaller values of β, after a short transient period, the agent stops moving. The trajectory of the agent during the transient period resembles an inward spiral. As β increases, a bump of activity appears in the network, leading to a motion along a circular trajectory is observed. The motion pattern during the transient time, where the agent's speed is increasing, is a growing (outward) spiral. As β increases further, the agent's speed increases and it saturates for large β. The trajectory remains a circular trajectory. Parameter values: $N_s = 100$, $\sigma = 0.4$, $v_0 = 0.05$, $dt = 0.3$, $h_b = 0$. The agent moves in a periodic space with linear size, $L = 1000$.

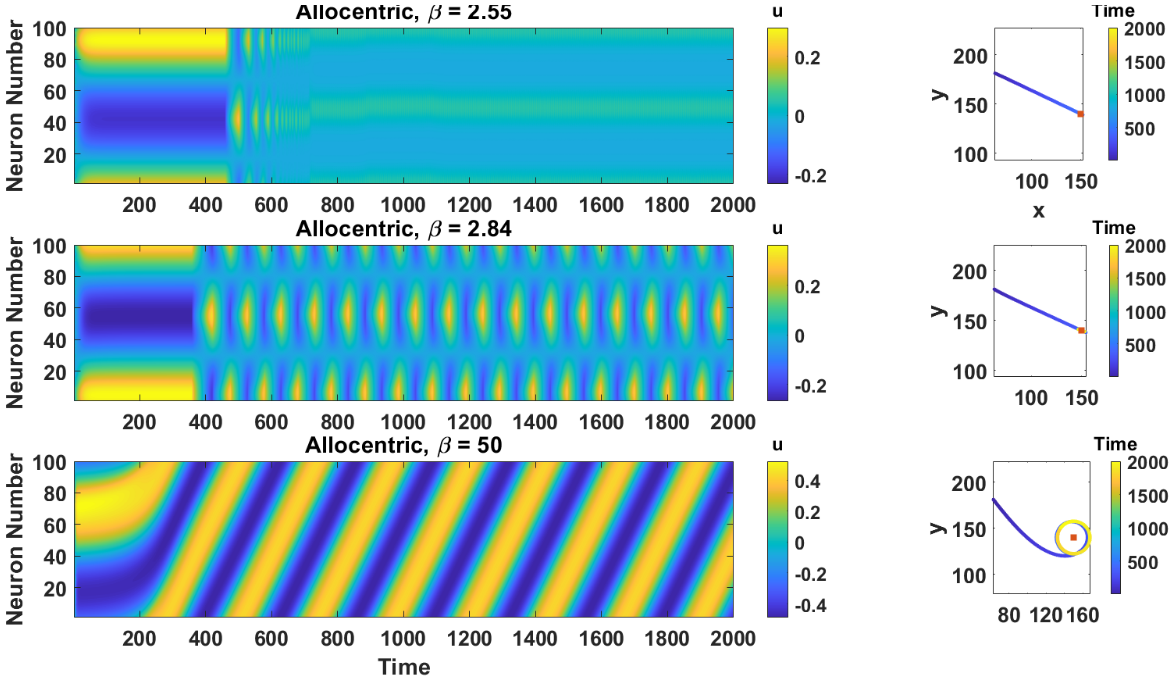

Supplementary Figure. 75: Movement patterns with an allocentric representation of space and during tracking a stationary target in the neural field model. The network activity as a function of time (left) and the resulting trajectories (right) for different values of β, indicated on each panel are shown. The agent possesses an allocentric representation of space. For all the values of β, the agent moves towards the target. Large values of β can lead to a circular trajectory around the target once the agent reaches the target. Parameter values: $N_s = 100$, $\sigma = 0.4$, $v_0 = 0.05$, $dt = 0.3$, $h_b = 0$. The agent moves in a periodic space with linear size, $L = 1000$.

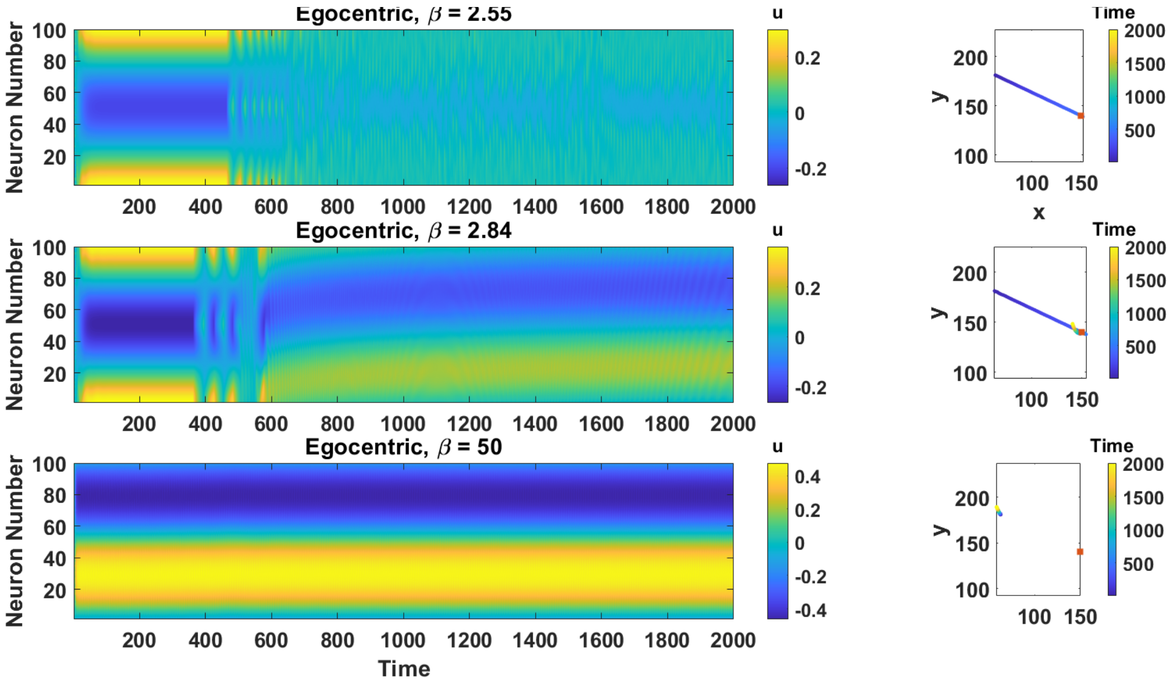

Supplementary Figure. 76: Movement patterns with an egocentric representation of space and during tracking a stationary target in the neural field model. The network activity as a function of time (left) and the resulting trajectories (right) for different values of β, indicated on each panel are shown. The agent has an egocentric representation of space. For small values of β, the agent moves towards the target. While for the small enough values of β, the agent stays close to the target, for larger values, it either moves away from the target in a coil-shaped trajectory or fails to reach the target. Parameter values: $N_s = 100$, $\sigma = 0.4$, $v_0 = 0.05$, $dt = 0.3$, $h_b = 0$. The agent moves in a periodic space with linear size, $L = 1000$.

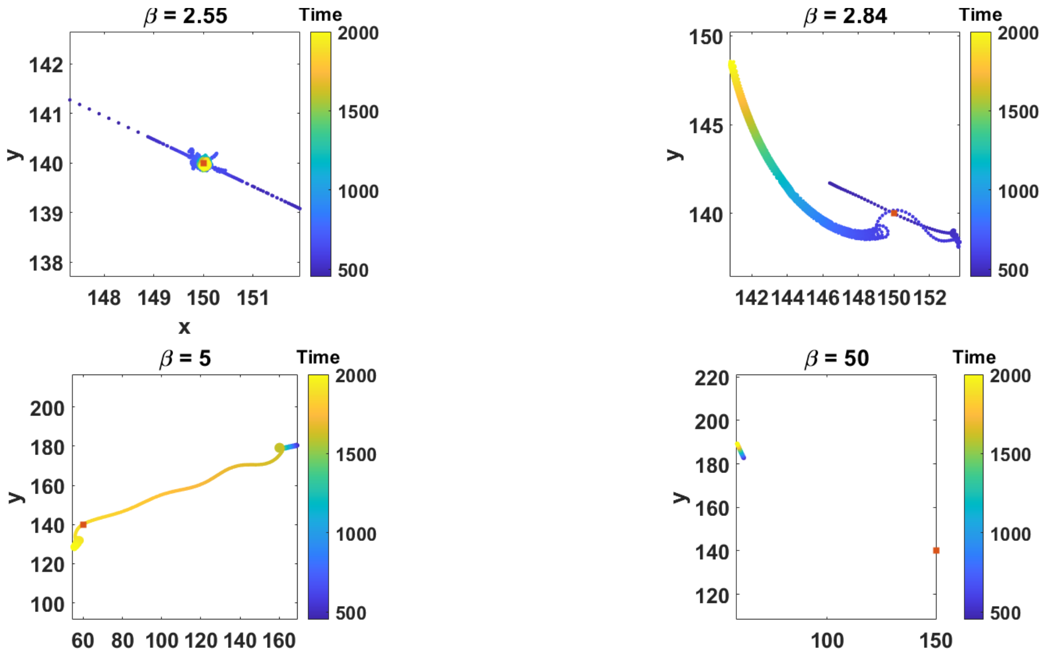

Supplementary Figure. 77: Movement patterns with an egocentric representation of space and during tracking a stationary target in the neural field model. The movement patterns in the last 1500 timesteps during tracking a fixed target are shown. Parameter values: $N_s = 100$, $\sigma = 0.4$, $v_0 = 0.05$, $dt = 0.3$, $h_b = 0$. The agent moves in a periodic space with linear size, $L = 1000$.

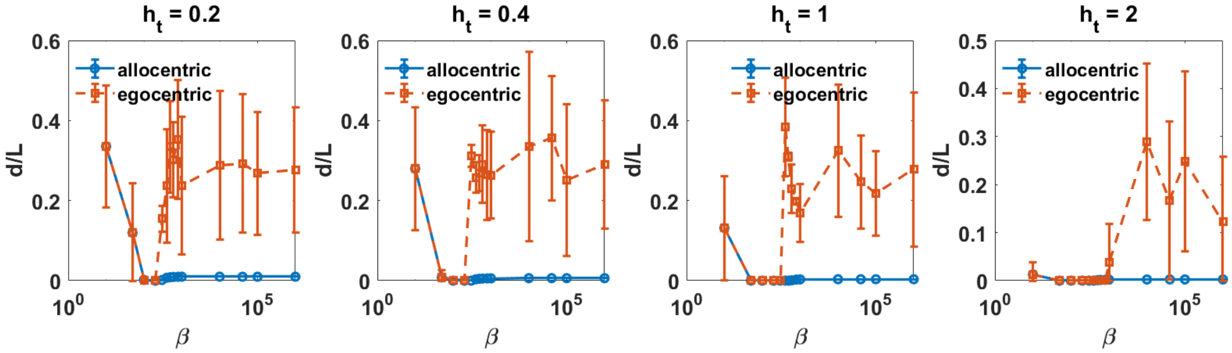

Supplementary Figure. 78: Individual information acquisition in the neural field model. The average distance of an agent from a fixed target with an allocentric and egocentric representation of space as a function of β is plotted. For too small β, the agent does not move. For larger β, the agent moves, reaches, and stays close to the target when possessing an allocentric representation of space. However, with an egocentric representation of space, the agent fails to reach the target for too large β. The value of β above which the agent starts to move towards the target decreases by increasing the attractiveness of the target, h_t. Parameter values: $N_s = 100$, $\sigma = 0.4$, $v_0 = 0.05$, $dt = 0.3$, $h_b = 0$. The agent moves in a periodic space with linear size, $L = 1000$.

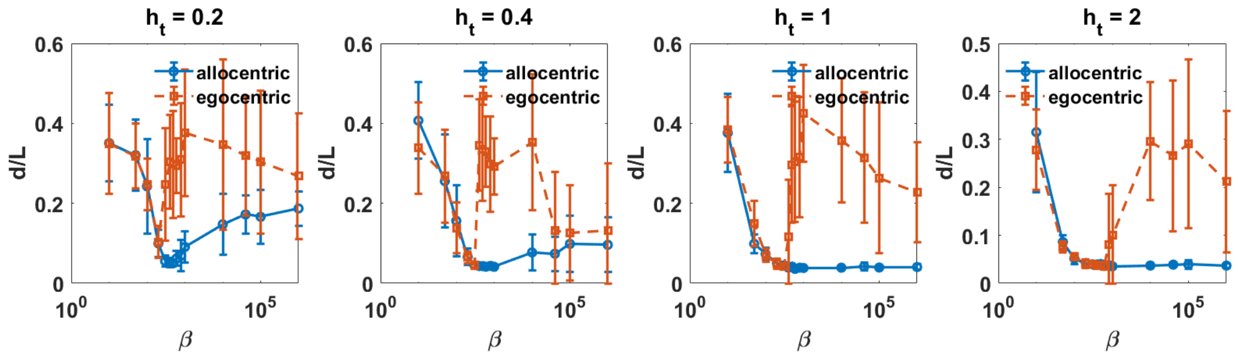

Supplementary Figure. 79: Individual information acquisition in the neural field model. The average distance of an agent from a moving target with an allocentric and egocentric representation of space as a function of β is plotted. For too small β, the agent does not move. For larger β, the agent moves, reaches, and stays close to the target when possessing an allocentric representation of space. The distance of the agent to the target for small h_t is minimized for medium values of β. However, with an egocentric representation of space, the agent fails to reach a target for too large β. Large error bars with an egocentric representation of space result from the fact that the agent's behavior shows stronger variability in different runs. Parameter values: $N_s = 100$, $\sigma = 0.4$, $v_0 = 0.05$, $dt = 0.3$, $h_b = 0$. The agent moves in a periodic space with linear size, $L = 1000$.

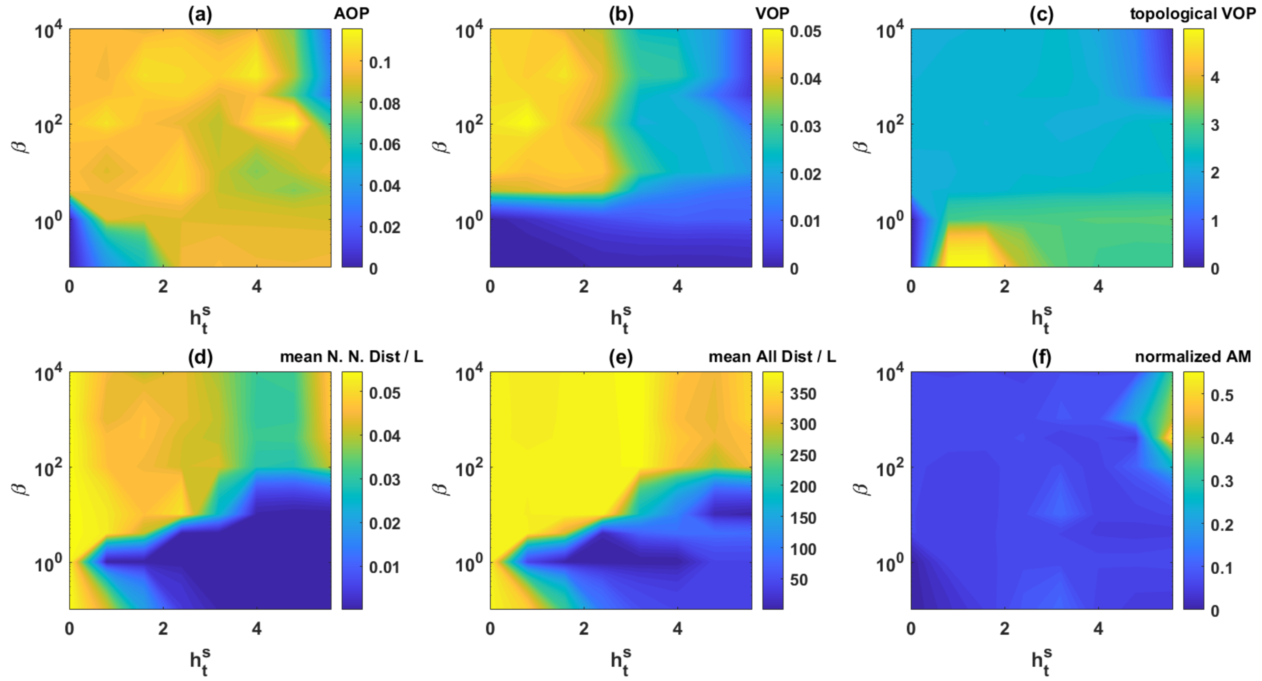

Supplementary Figure. 80: Contour plots of collective motion metrics in $\beta - h_t^s$ space in groups of 80 agents in the neural field model with an egocentric representation of space. The collective motion order parameters are color plotted as a function of the social attraction (h_s^0) and inverse neural noise (β). For a social attraction that is too small, the agents move independently. As social attraction increases, collective motion is not observed. Rather, the collective forms an aggregation with low global order (global angular order parameter (AOP in (a)) and vectorial order parameter (VOP in (b))), low local order (topological VOP in (c)), and low or relatively low mean distance between agents (mean nearest neighbor distance (d) and the distance between all pairs (e)). While for small β, agents coalesce in the aggregation phase, for too large values of β, agents keep a distance in the aggregation phase, or they are decomposed into subgroups with slow movement. Parameter values: $N_s = 100$, $\sigma = 0.4$, $v_0 = 0.05$, $dt = 0.3$, $h_b = 0$. 80 agents move in a periodic space with linear size, $L = 1000$.

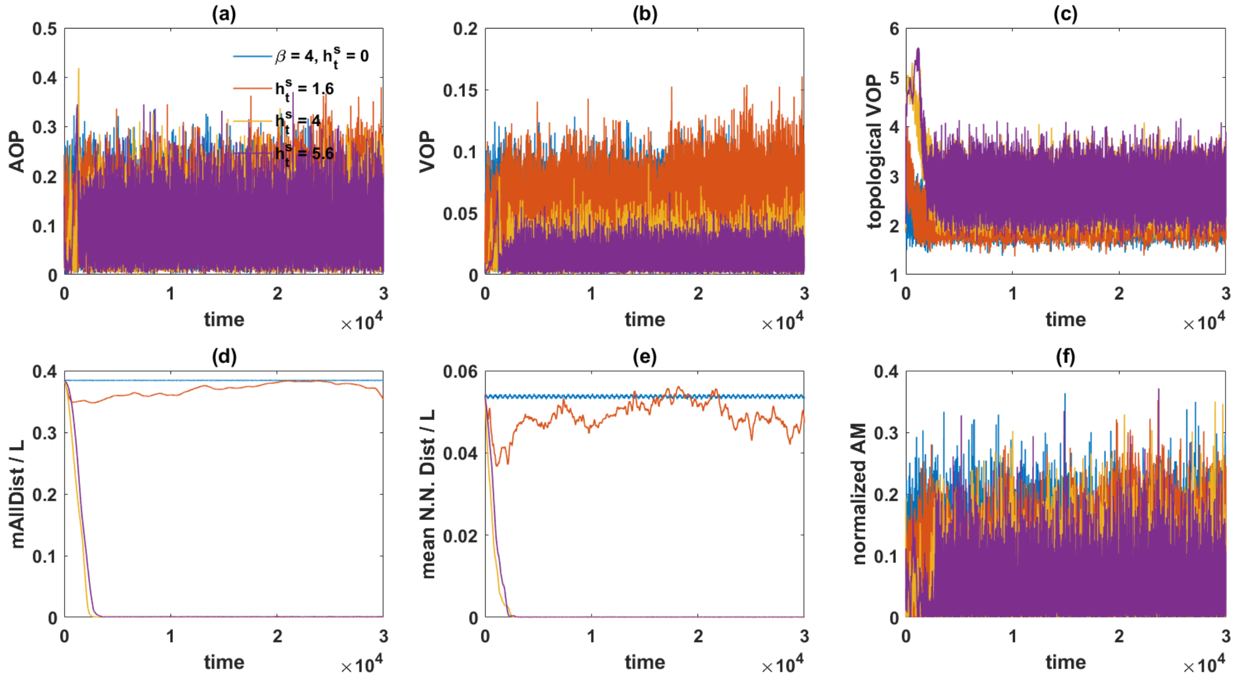

Supplementary Figure. 81: Time dependence of collective movement in groups of 80 agents with an egocentric representation of space in the neural field model. Angular order parameter (AOP in (a)), vectorial order parameter (VOP in (b)), topological VOP (c), the mean distance between all the pairs (normalized by the arena size, L, mAllDist/L in (d)), mean nearest neighbor distance (N.N. Dist/L in (e)), and normalized angular momentum, (AM in (f)) for four different values of total social attraction indicated in the legend as a function of time is shown. By increasing social attraction, the collective shows a transition from disordered motion, where individuals move independently, to an aggregation, where the distance between individuals decreases. Global order and local order remain small for all values of social attraction. The bump for small social attraction in local order in initial time results from the agents moving toward each other starting from random locations, in the initial stages of simulations. Parameter values: $N_s = 100$, $\sigma = 0.4$, $v_0 = 0.05$, $h_b = 0$, $dt = 0.3$, and $\beta = 10000$. $g = 80$ agents move in a periodic space with linear size, $L = 1000$.

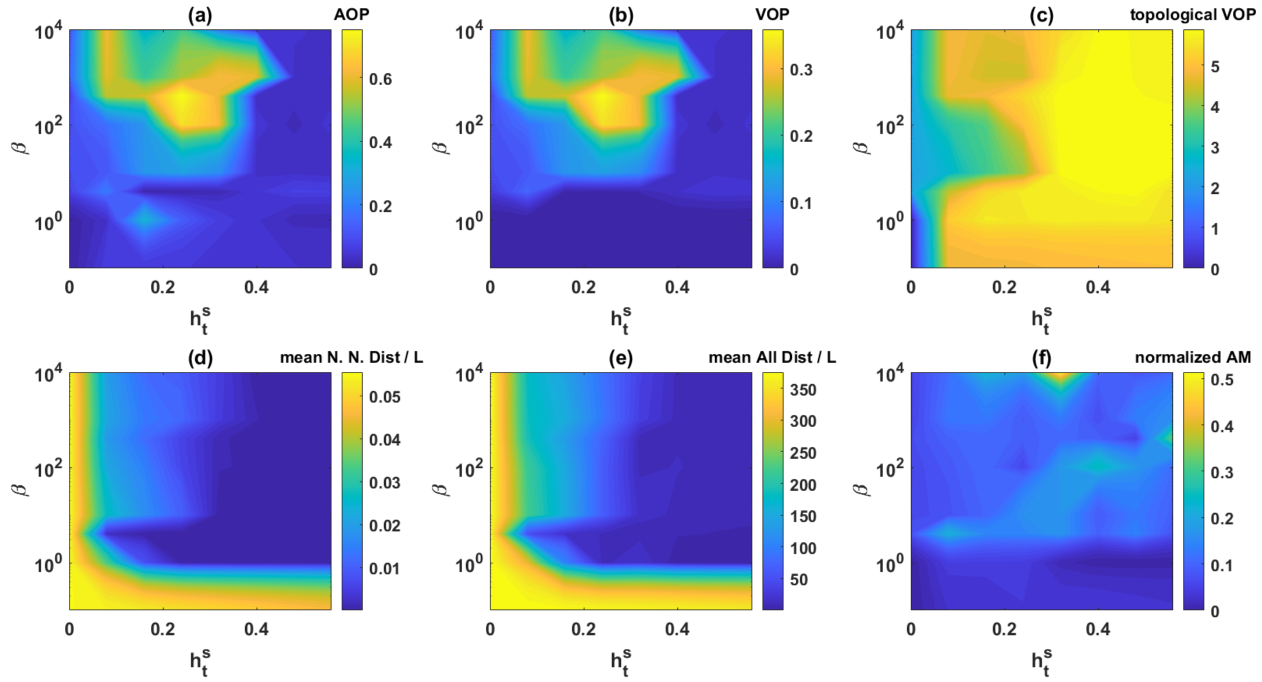

Supplementary Figure. 82: Contour plots of collective motion metrics in $\beta - h_t^s$ space in groups of 80 agents in the neural field model with an allocentric representation of space. The collective motion order parameters are color plotted as a function of the social attraction (h_s^0) and inverse neural noise (β). For a social attraction that is too small, the agents move independently. As social attraction increases, the collective first shows a phase transition to a collective motion phase with high global (global angular order parameter (AOP in (a)) and vectorial order parameter (VOP in (b))) and local (topological VOP in (c)) order, and then a second phase transition to an aggregation phase with high local order but often small global order and low mean distance between agents (mean nearest neighbor distance (d) and the distance between all pairs (e)). While the agents coalesce for high social attraction, the group of packed agents often shows a small collective motion for too high social attraction. A relatively high (time-averaged) normalized angular momentum (f) indicates milling patterns are observed in the neural field model. Parameter values: $N_s = 100$, $\sigma = 0.4$, $v_0 = 0.05$, $dt = 0.3$, $h_b = 0$. 80 agents move in a periodic space with linear size, $L = 1000$.

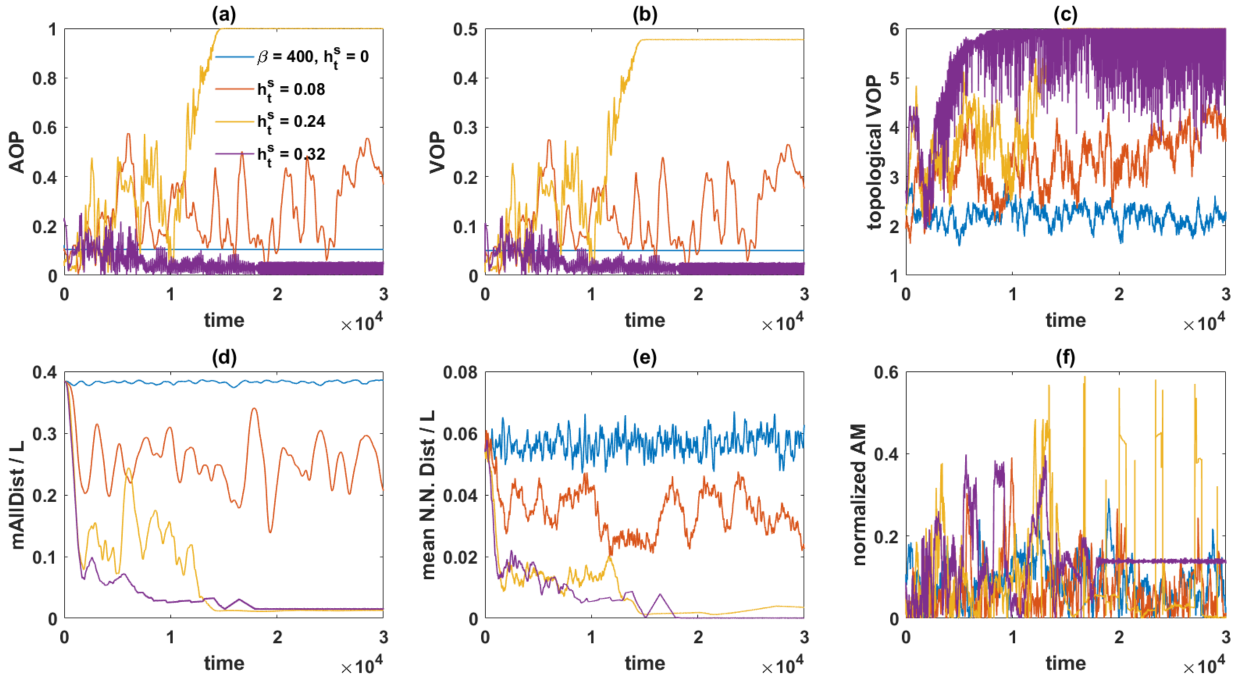

Supplementary Figure. 83: Time dependence of collective movement in groups of 80 agents with an allocentric representation of space in the neural field model. Angular order parameter (AOP in (a)), vectorial order parameter (VOP in (b)), topological VOP (c), the mean distance between all the pairs (normalized by the arena size, L, mAllDist/L in (d)), mean nearest neighbor distance (N.N. Dist/L in (e)), and normalized angular momentum, (AM in (f)) for four different values of total social attraction indicated in the legend as a function of time is shown. In the absence of social attraction, individuals do not interact and no collective motion is observed. For moderate values of total social attraction, collective motion is observed. For larger values of social attraction, $h_s^0 = 0.4$, cohesive motion with low global order and low distance between individuals is observed. Parameter values: $N_s = 100$, $\sigma = 0.4$, $v_0 = 0.05$, $h_b = 0$, $dt = 0.3$, and $\beta = 10000$. $g = 80$ agents move in a periodic space with linear size, $L = 1000$.

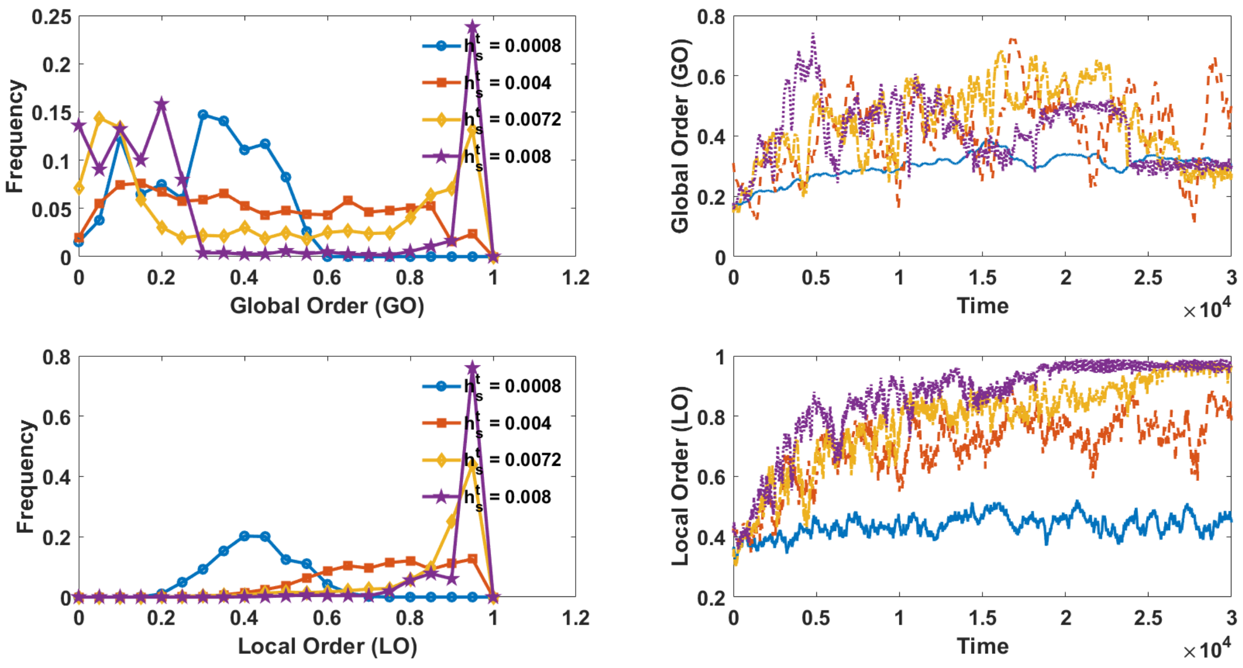

Supplementary Figure. 84: Order-disorder transition in populations of $g = 20$ individuals in the neural field model. The left panels show the distribution of Global Order (angular order parameter) and local order (topological order parameter) in groups of $g = 20$ agents with an allocentric representation of space. The right panels show example time series of the same variables as a function of time. Parameter values: $N_s = 100$, $\sigma = 2\pi/N_s$, $\nu = 0.5$, $v_0 = 0.05$, $\beta = 1000$, $h_b = 0$, and $dt = 0.3$. Agents move in a periodic space with linear size, $L = 1000$. The simulations are performed for 30000 timesteps, and the last 15000 timesteps in a sample of 5 simulations are used to calculate distributions.

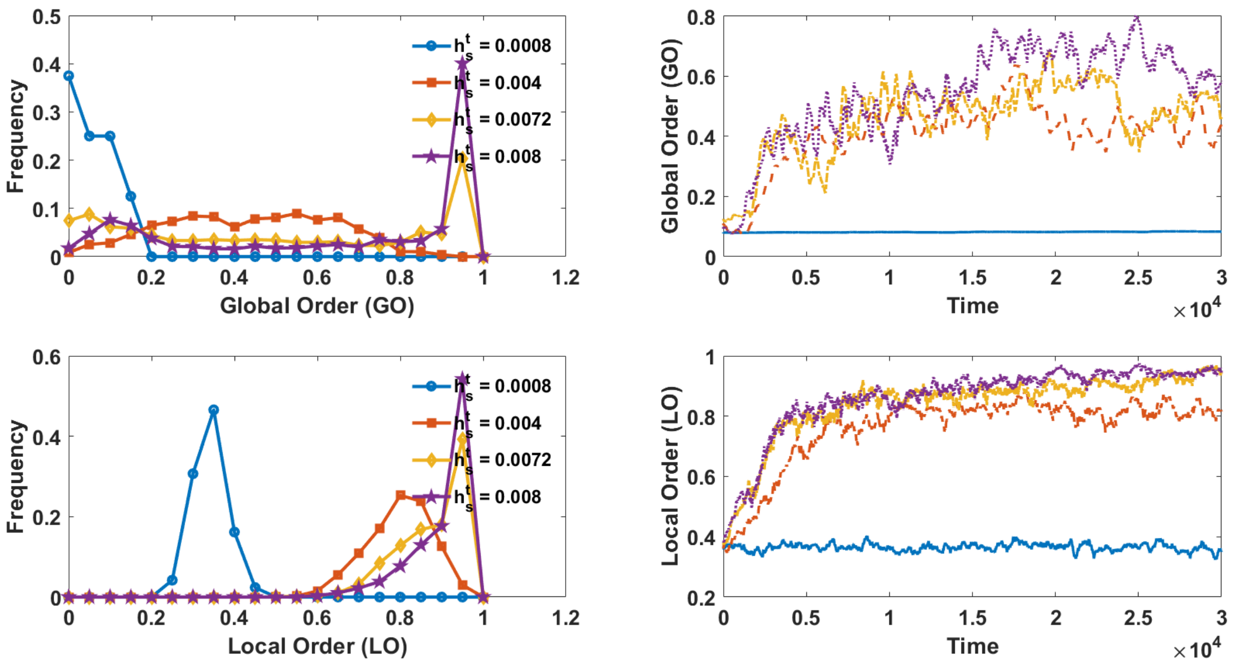

Supplementary Figure. 85: Collective motion-aggregation phase transition in populations of $g = 80$ individuals in the neural field model. The left panels show the distribution of Global Order (angular order parameter) and local order (topological order parameter) in groups of $g = 80$ agents with an allocentric representation of space. The right panels show example time series of the same variables as a function of time. Parameter values: $N_s = 100$, $\sigma = 2\pi/N_s$, $\nu = 0.5$, $v_0 = 0.05$, $\beta = 1000$, $h_b = 0$, and $dt = 0.3$. Agents move in a periodic space with linear size, $L = 1000$. The simulations are performed for 30000 timesteps, and the last 15000 timesteps in a sample of 8 simulations are used to calculate distributions.

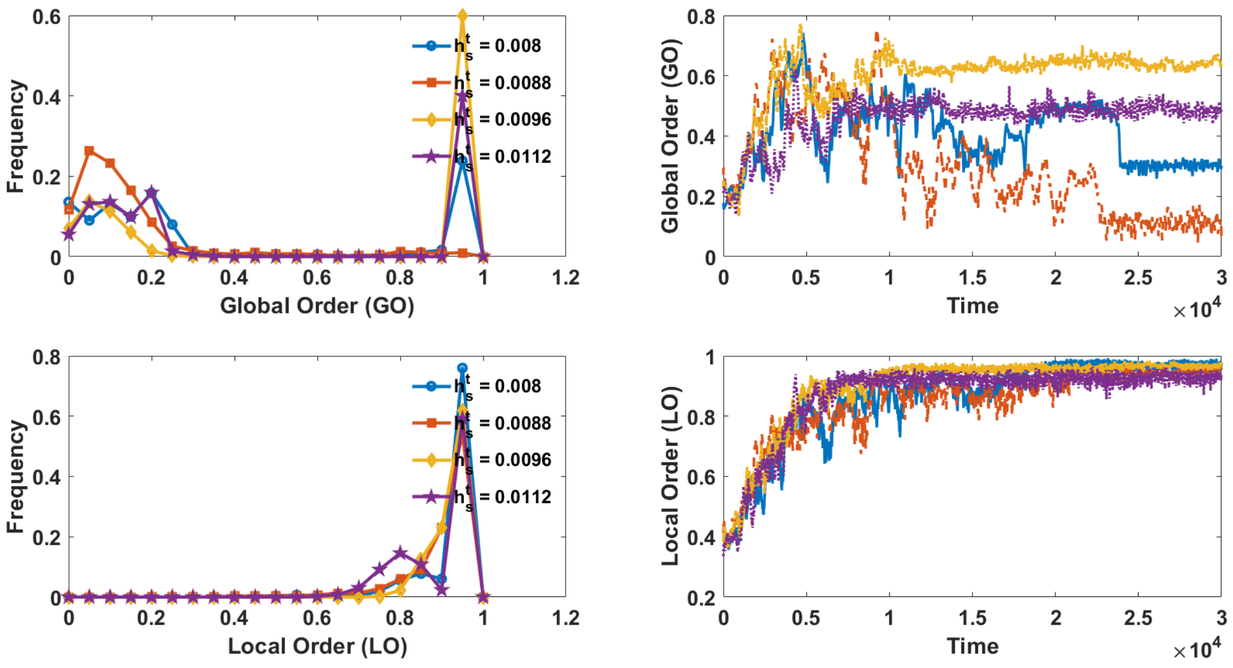

Supplementary Figure. 86: Collective motion-aggregation phase transition in populations of $g = 20$ individuals in the neural field model. The left panels show the distribution of Global Order (angular order parameter) and local order (topological order parameter) in groups of $g = 20$ agents with an allocentric representation of space. The right panels show example time series of the same variables as a function of time. Parameter values: $N_s = 100$, $\sigma = 2\pi/N_s$, $\nu = 0.5$, $v_0 = 0.05$, $\beta = 1000$, $h_b = 0$, and $dt = 0.3$. Agents move in a periodic space with linear size, $L = 1000$. The simulations are performed for 30000 timesteps, and the last 15000 timesteps in a sample of 5 simulations are used to calculate distributions.

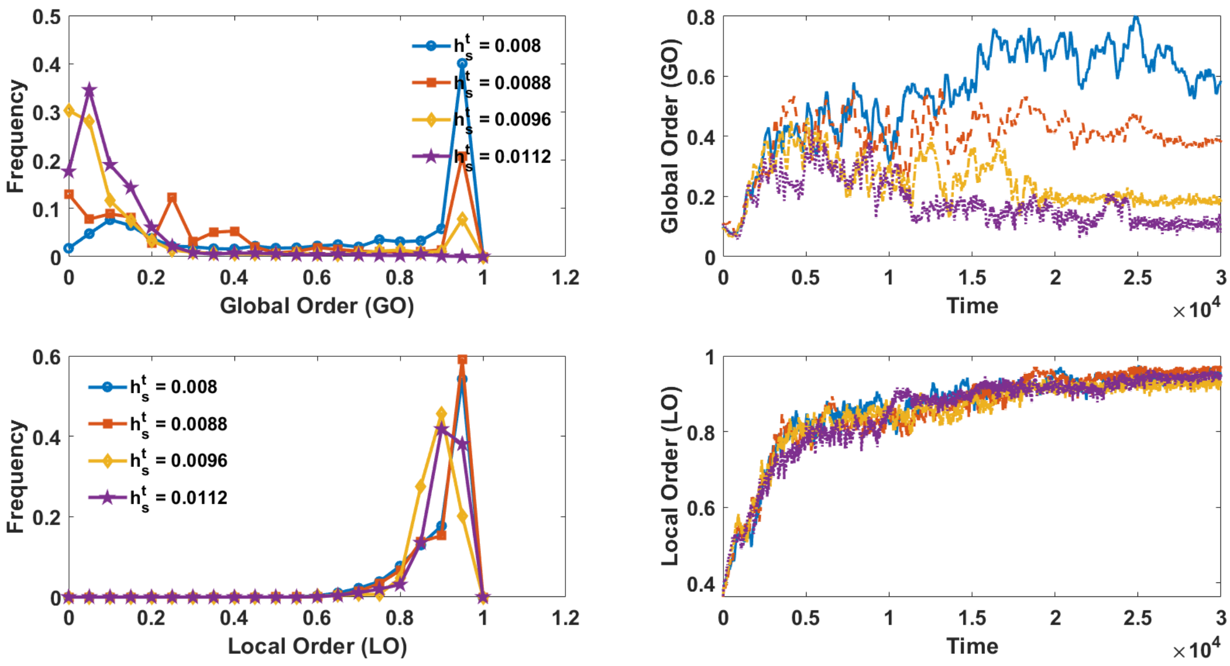

Supplementary Figure. 87: Order-disorder transition in populations of $g = 80$ individuals in the neural field model. The left panels show the distribution of Global Order (angular order parameter) and local order (topological order parameter) in groups of $g = 80$ agents with an allocentric representation of space. The right panels show example time series of the same variables as a function of time. Parameter values: $N_s = 100$, $\sigma = 2\pi/N_s$, $\nu = 0.5$, $v_0 = 0.05$, $\beta = 1000$, $h_b = 0$, and $dt = 0.3$. Agents move in a periodic space with linear size, $L = 1000$. The simulations are performed for 30000 timesteps, and the last 15000 timesteps in a sample of 8 simulations are used to calculate distributions.

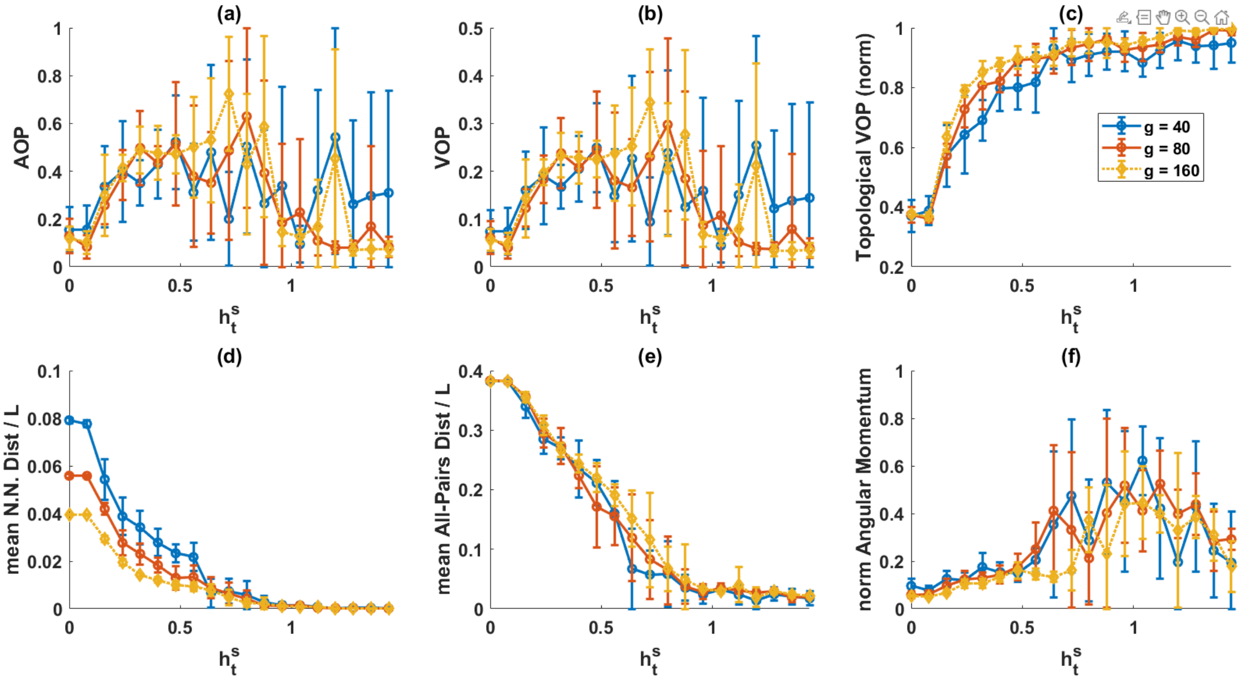

Supplementary Figure. 88: Dependence on the number of agents, g in the neural field model. Different measures of collective motion of agents are shown as a function of social attraction and for three different population size, g. (a) to (f) show the global angular order parameter (AOP, (a)), vectorial order parameter (VOP, (b)), topological VOP (c), mean nearest neighbor distance (d), and all-pair distances (e), normalized angular momentum (f). $N_s = 100$, $\nu = 0.5$, $v_0 = 0.05$, $\beta = 1000$, $h_b = 0$, and $dt = 0.3$. g agents move in a periodic space with linear size, $L = 1000$. g is shown in the legend. Simulations are run for 30000 timesteps, and time averages are taken over the last 5000 timesteps. An average over a sample of 8 simulations is taken.

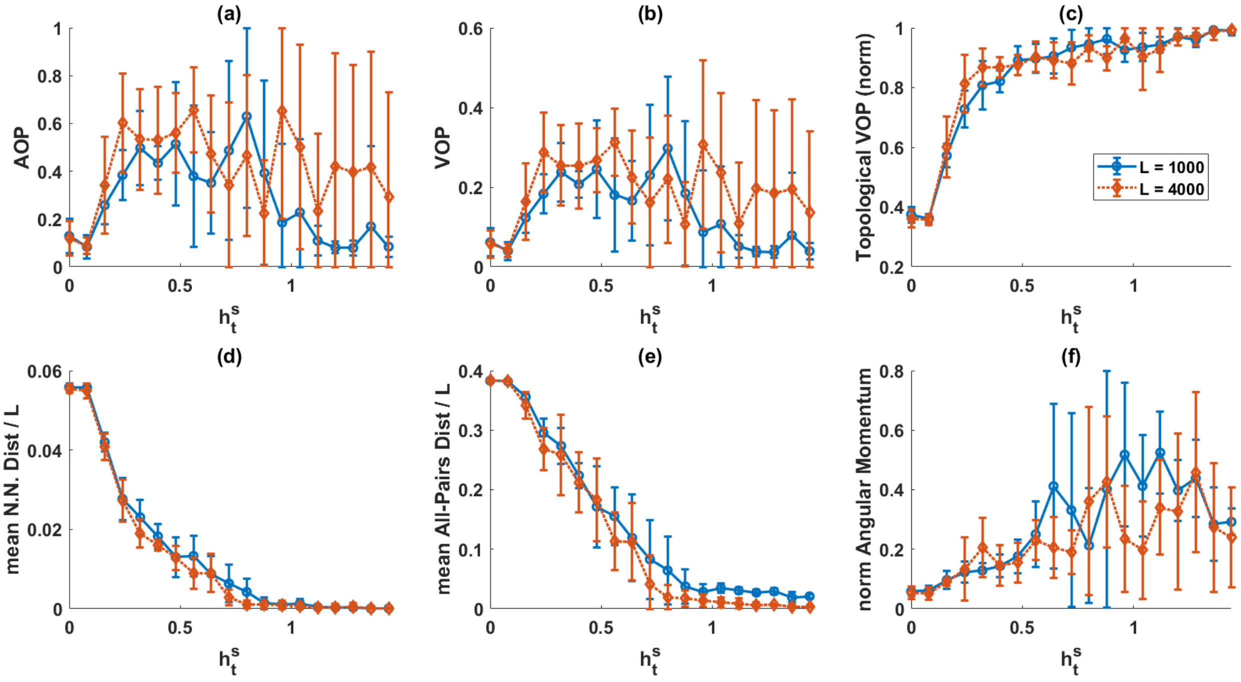

Supplementary Figure. 89: Dependence on the arena size, L in the neural field model. Different measures of collective motion of agents are shown as a function of social attraction and for two different arena size, L. (a) to (f) show the global angular order parameter (AOP, (a)), vectorial order parameter (VOP, (b)), topological VOP (c), mean nearest neighbor distance (d), and all-pair distances (e), normalized angular momentum (f). $N_s = 100$, $\nu = 0.5$, $\sigma = 0.1$, $v_0 = 0.05$, $\beta = 1000$, $h_b = 0$, $\beta = 1000$, and $dt = 0.3$. 80 agents move in a periodic space with linear size, L. L is shown in the legend. Simulations are run for 30000 timesteps, and time averages are taken over the last 5000 timesteps. An average over a sample of 8 simulations is taken.

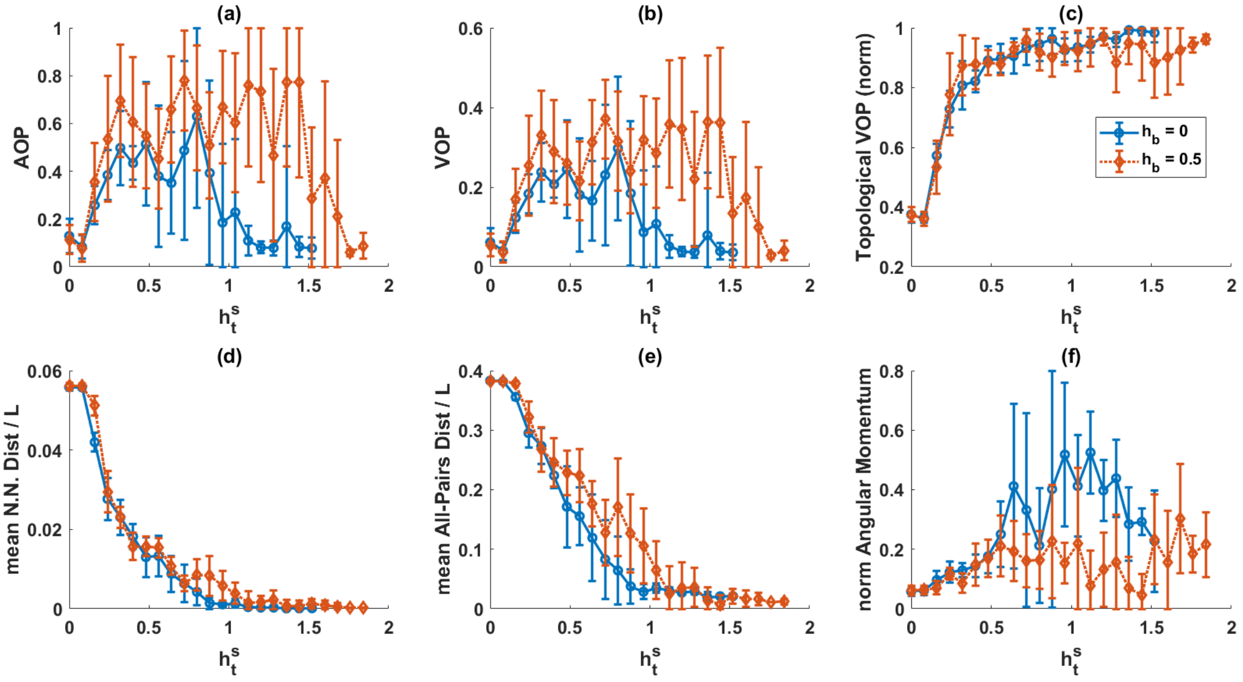

Supplementary Figure. 90: Dependence on the global inhibition, h_b in the neural field model. Different measures of collective motion of agents are shown as a function of social attraction and for two different values of agents' network's global inhibition, h_b. (a) to (f) show the global angular order parameter (AOP, (a)), vectorial order parameter (VOP, (b)), topological VOP (c), mean nearest neighbor distance (d), and all-pair distances (e), normalized angular momentum (f). $N_s = 100$, $\nu = 0.5$, $v_0 = 0.05$, $\beta = 1000$, $\beta = 1000$, and $dt = 0.3$. 80 agents move in a periodic space with linear size, $L = 1000$. h_b is shown in the legend. Simulations are run for 30000 timesteps, and time averages are taken over the last 5000 timesteps. An average over a sample of 8 simulations is taken.

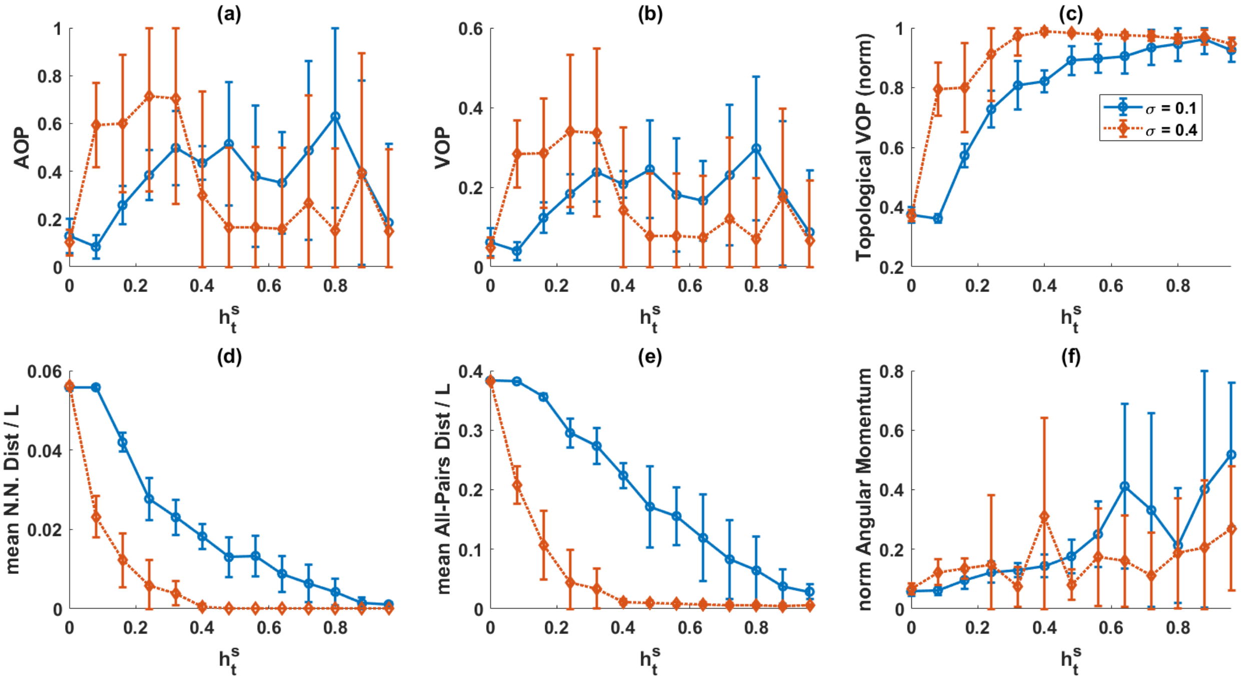

Supplementary Figure. 91: Dependence on the width of the receptive field, σ in the neural field model. Different measures of collective motion of agents are shown as a function of social attraction and for two different values of the width of the receptive field, σ. (a) to (f) show the global angular order parameter (AOP, (a)), vectorial order parameter (VOP, (b)), topological VOP (c), mean nearest neighbor distance (d), and all-pair distances (e), normalized angular momentum (f). $N_s = 100$, $\nu = 0.5$, $v_0 = 0.05$, $\beta = 1000$, $h_b = 0$, $\beta = 1000$, and $dt = 0.3$. 80 agents move in a periodic space with linear size, $L = 1000$. σ is shown in the legend. Simulations are run for 30000 timesteps, and time averages are taken over the last 5000 timesteps. An average over a sample of 8 simulations is taken.

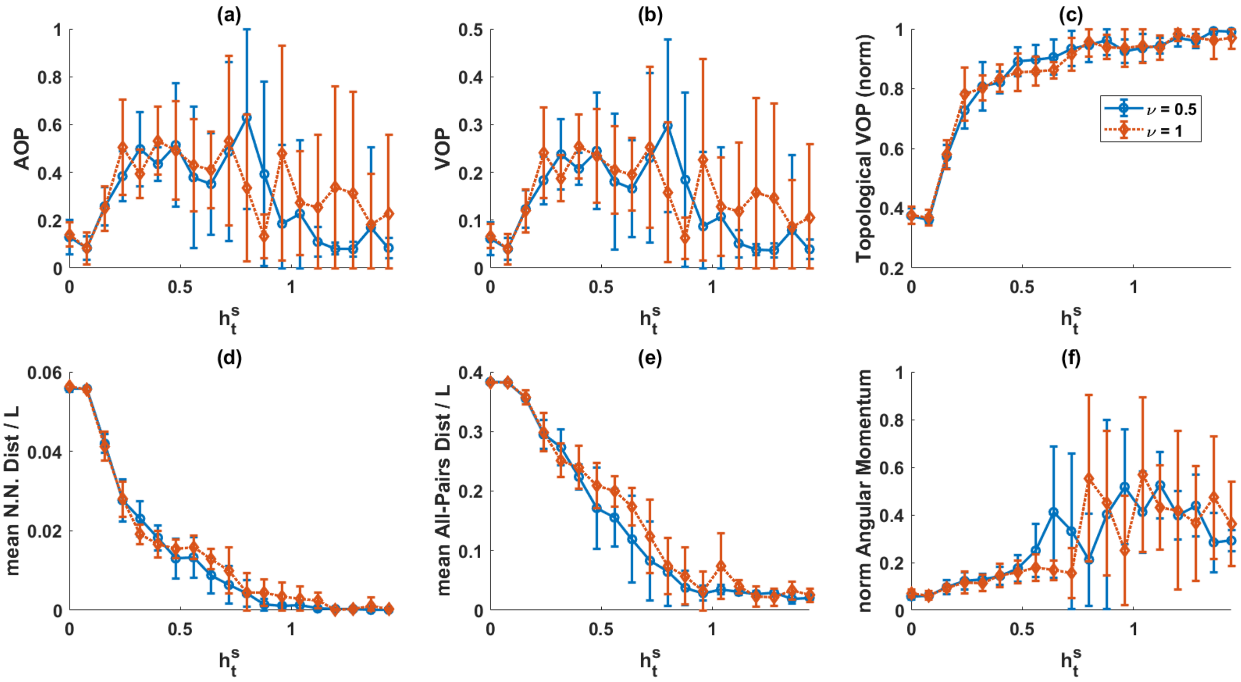

Supplementary Figure. 92: Dependence on the neural tunning parameter, ν in the neural field model. Different measures of collective motion of agents are shown as a function of social attraction and for two different values of the synaptic connectivity's tunning parameter, ν. (a) to (f) show the global angular order parameter (AOP, (a)), vectorial order parameter (VOP, (b)), topological VOP (c), mean nearest neighbor distance (d), and all-pair distances (e), normalized angular momentum (f). $N_s = 100$, $\sigma = 0.1$, $v_0 = 0.05$, $\beta = 1000$, $h_b = 0$, $\beta = 1000$, and $dt = 0.3$. 80 agents move in a periodic space with linear size, $L = 1000$. ν is shown in the legend. Simulations are run for 30000 timesteps, and time averages are taken over the last 5000 timesteps. An average over a sample of 8 simulations is taken.

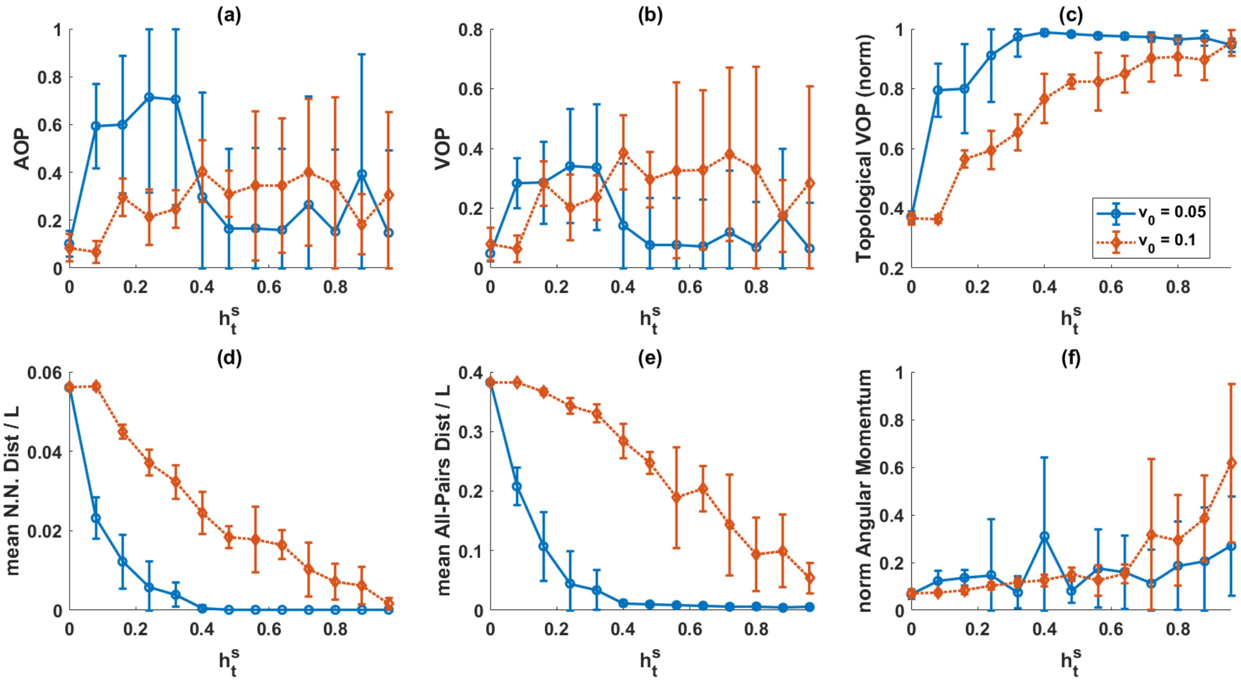

Supplementary Figure. 93: Dependence on the speed constant, v_0 in the neural field model. Different measures of collective motion of agents are shown as a function of social attraction and for two different values of the agents' speed constaqnt, v_0. (a) to (h) show, the global angular order parameter (AOP, (a)), vectorial order parameter (VOP, (b)), local VOP, both topological (c) and geometric (d), mean nearest neighbor distance (e), and all-pair distances (f), normalized and total angular momentum (AM, (g) and (h)). $N_s = 100$, $\nu = 0.5$, $\beta = 1000$, $h_b = 0$, $\beta = 1000$, and $dt = 0.3$. 80 agents move in a periodic space with linear size, $L = 1000$. v_0 is shown in the legend. Simulations are run for 30000 timesteps, and time averages are taken over the last 5000 timesteps. An average over a sample of 8 simulations is taken.

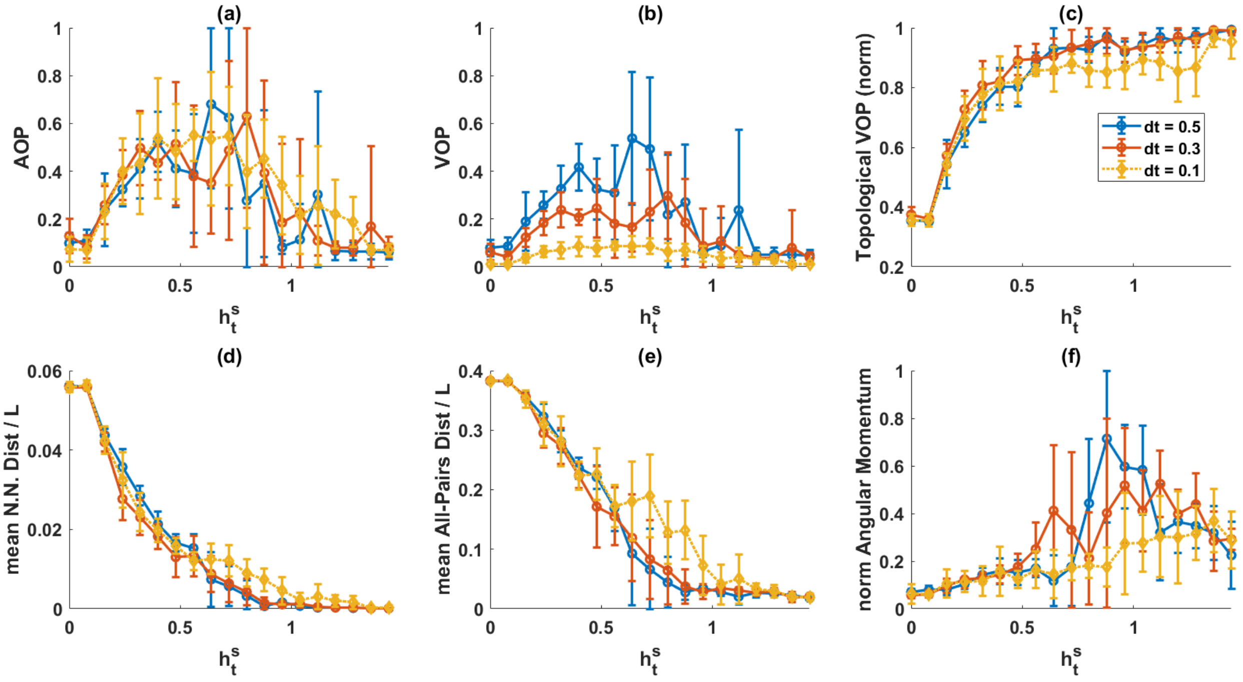

Supplementary Figure. 94: Dependence on the integration constant, dt. Different measures of collective motion of agents are shown as a function of social attraction and for three different values of the integration constant, dt. (a) to (f) show the global angular order parameter (AOP, (a)), vectorial order parameter (VOP, (b)), topological VOP (c), mean nearest neighbor distance (d), and all-pair distances (e), normalized angular momentum (f). $N_s = 100$, $\sigma = 0.1$, $v_0 = 0.05$, $\nu = 0.5$, $\beta = 1000$, and $h_b = 0$. 80 agents move in a periodic space with linear size, $L = 1000$. dt is shown in the legend. Simulations are run for 30000 timesteps, and time averages are taken over the last 5000 timesteps. An average over a sample of 8 simulations is taken.

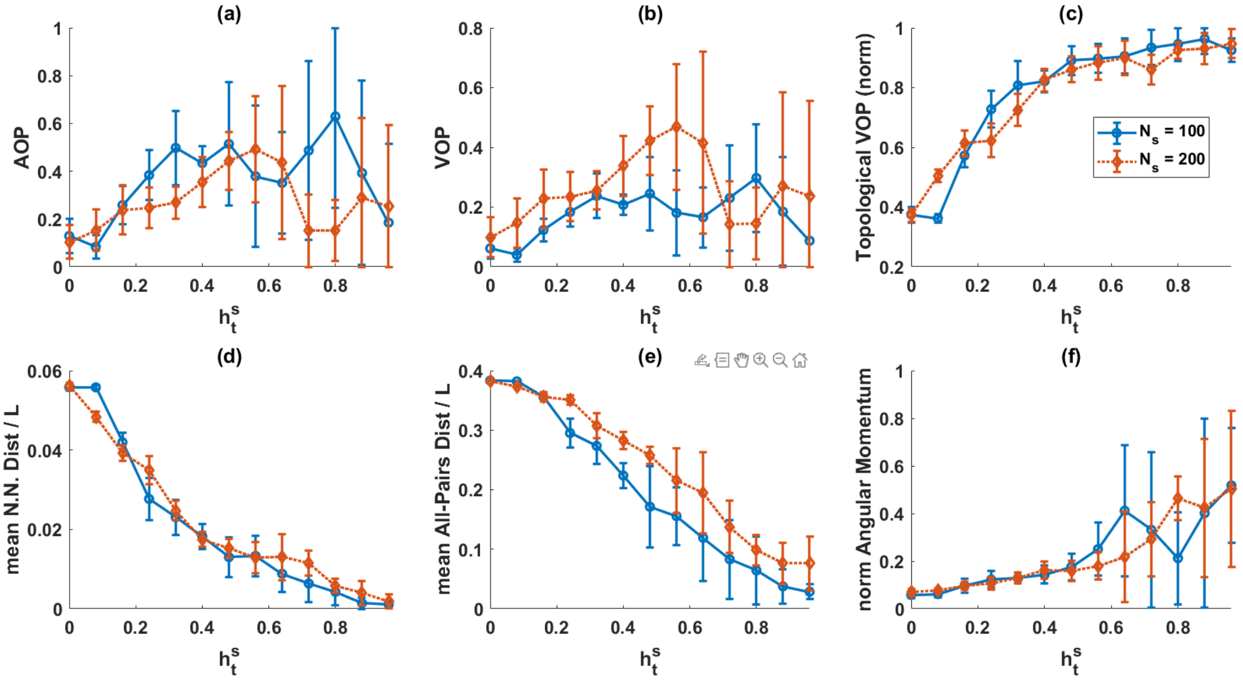

Supplementary Figure. 95: Dependence on the number of neurons, N_s. Different measures of collective motion of agents are shown as a function of social attraction and for two different values of the number of neurons, N_s. (a) to (f) show the global angular order parameter (AOP, (a)), vectorial order parameter (VOP, (b)), topological VOP (c), mean nearest neighbor distance (d), and all-pair distances (e), normalized angular momentum (f). Parameter values: $\sigma = 0.1$, $v_0 = 0.05$, $\nu = 0.5$, $\beta = 1000$, $dt = 0.3$, and $h_b = 0$. 80 agents move in a periodic space with linear size, $L = 1000$. dt is shown in the legend. Simulations are run for 30000 timesteps, and time averages are taken over the last 5000 timesteps. An average over a sample of 8 simulations is taken.

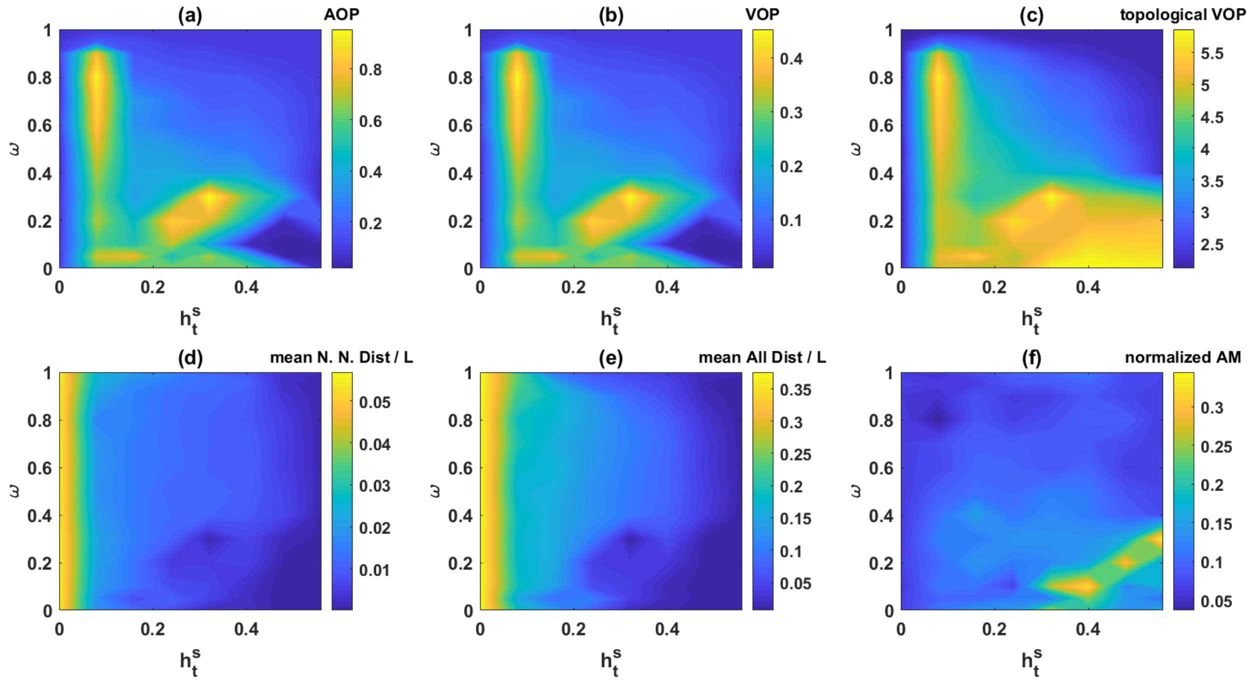

Supplementary Figure. 96: Contour plots of collective motion metrics in $\omega - h_t^s$ space in groups of 80 agents in the neural field model with switching between allocentric and egocentric representations of space. For smaller values of probability of egocentric representation, ω, as social attraction increases, the collective first shows a phase transition to a collective motion phase with high global (global angular order parameter (AOP in (a)) and vectorial order parameter (VOP in (b))) and local (topological VOP in (c)) order, and then a phase transition to an aggregation phase with high local order but often small global order and low mean distance between agents (mean nearest neighbor distance (d) and the distance between all pairs (e)). For larger values of ω, collective motion is still observed for small h_t^s. However, in the aggregation phase low local order is observed, similar to a purely egocentric representation of space. For too high ω, low global and local order is observed for all values of h_t^s. A relatively high normalized angular momentum (f) is observed for small ω close to the phase transition to aggregation. Parameter values: $N_s = 100$, $\sigma = 0.4$, $v_0 = 0.05$, $dt = 0.3$, $h_b = 0$, and $\beta = 1000$. 80 agents move in a periodic space with linear size, $L = 1000$.

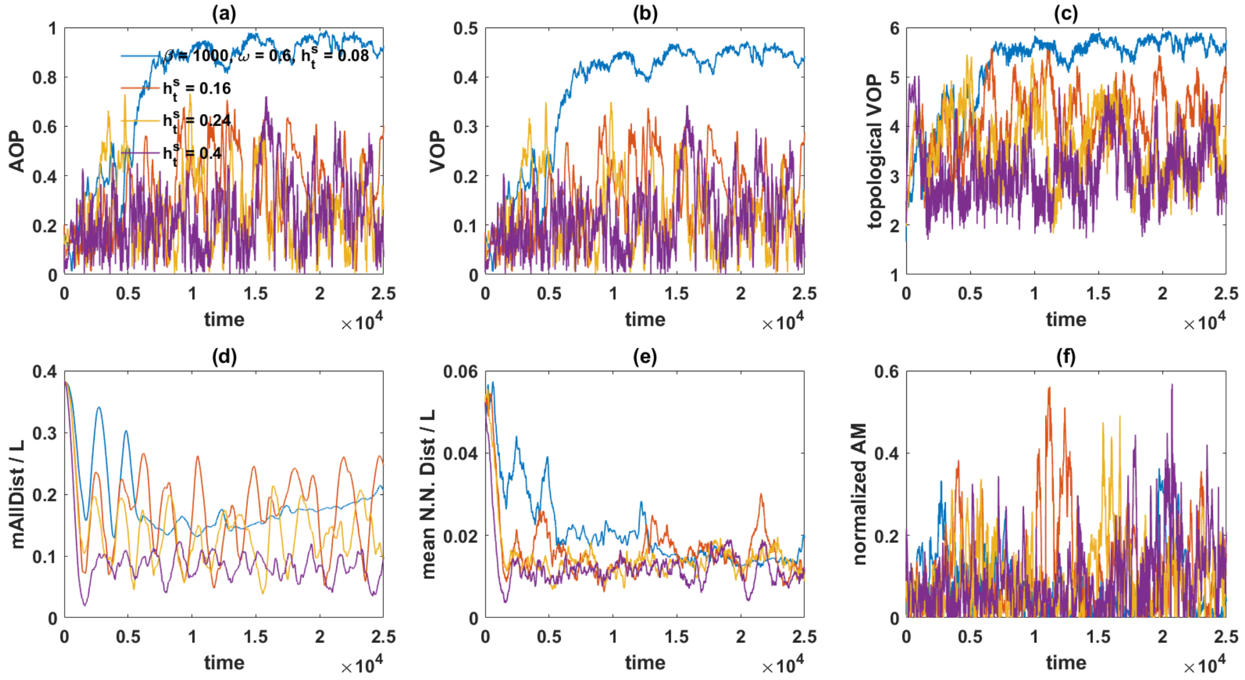

Supplementary Figure. 97: Time dependence of collective movement in groups of 80 agents with switch between allocentric and egocentric representations of space in the neural field model. Angular order parameter (AOP in (a)), vectorial order parameter (VOP in (b)), topological VOP (c), the mean distance between all the pairs (normalized by the arena size, L, mAllDist/L in (d)), mean nearest neighbor distance (N.N. Dist/L in (e)), and normalized angular momentum, (AM in (f)) for four different values of total social attraction indicated in the legend as a function of time is shown. While similar phases and phase transitions to that observed in the purely allocentric model is observed, switching between allocentric and egocentric representations stabilizes highly ordered motion in the collective motion phase ($h_t^s = 0.08$). Parameter values: $N_s = 100$, $\sigma = 0.4$, $v_0 = 0.05$, $h_b = 0$, $dt = 0.3$, and $\beta = 10000$. $g = 80$ agents move in a periodic space with linear size, $L = 1000$.

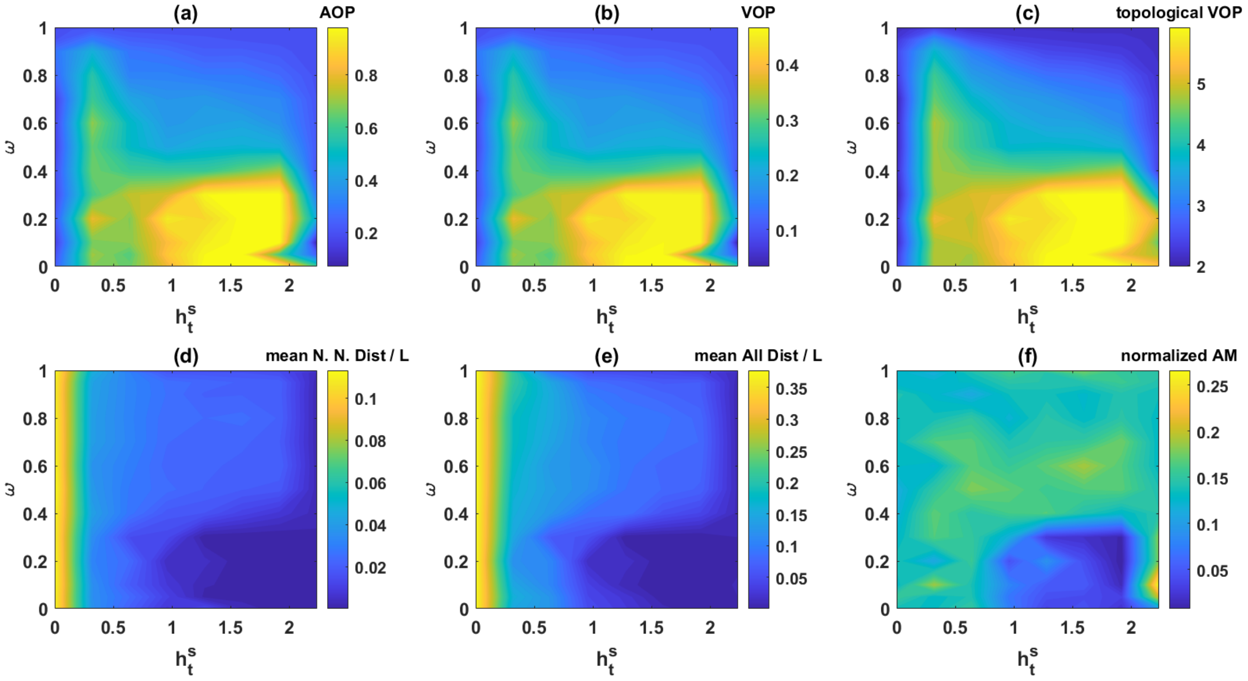

Supplementary Figure. 98: Contour plots of collective motion metrics in $\omega - h_t^s$ space in groups of 20 agents in the neural field model with switching between allocentric and egocentric representations of space. For smaller values of probability of egocentric representation, ω, as social attraction increases, the collective first shows a phase transition to a collective motion phase with high global (global angular order parameter (AOP in (a)) and vectorial order parameter (VOP in (b))) and local (topological VOP in (c)) order, and then a phase transition to an aggregation phase with high local order but often small global order and low mean distance between agents (mean nearest neighbor distance (d) and the distance between all pairs (e)). Compared to larger groups, in smaller groups lower order is observed for small h_t^s. Parameter values: $N_s = 100$, $\sigma = 0.4$, $v_0 = 0.05$, $dt = 0.3$, $h_b = 0$, and $\beta = 1000$. 20 agents move in a periodic space with linear size, $L = 1000$.

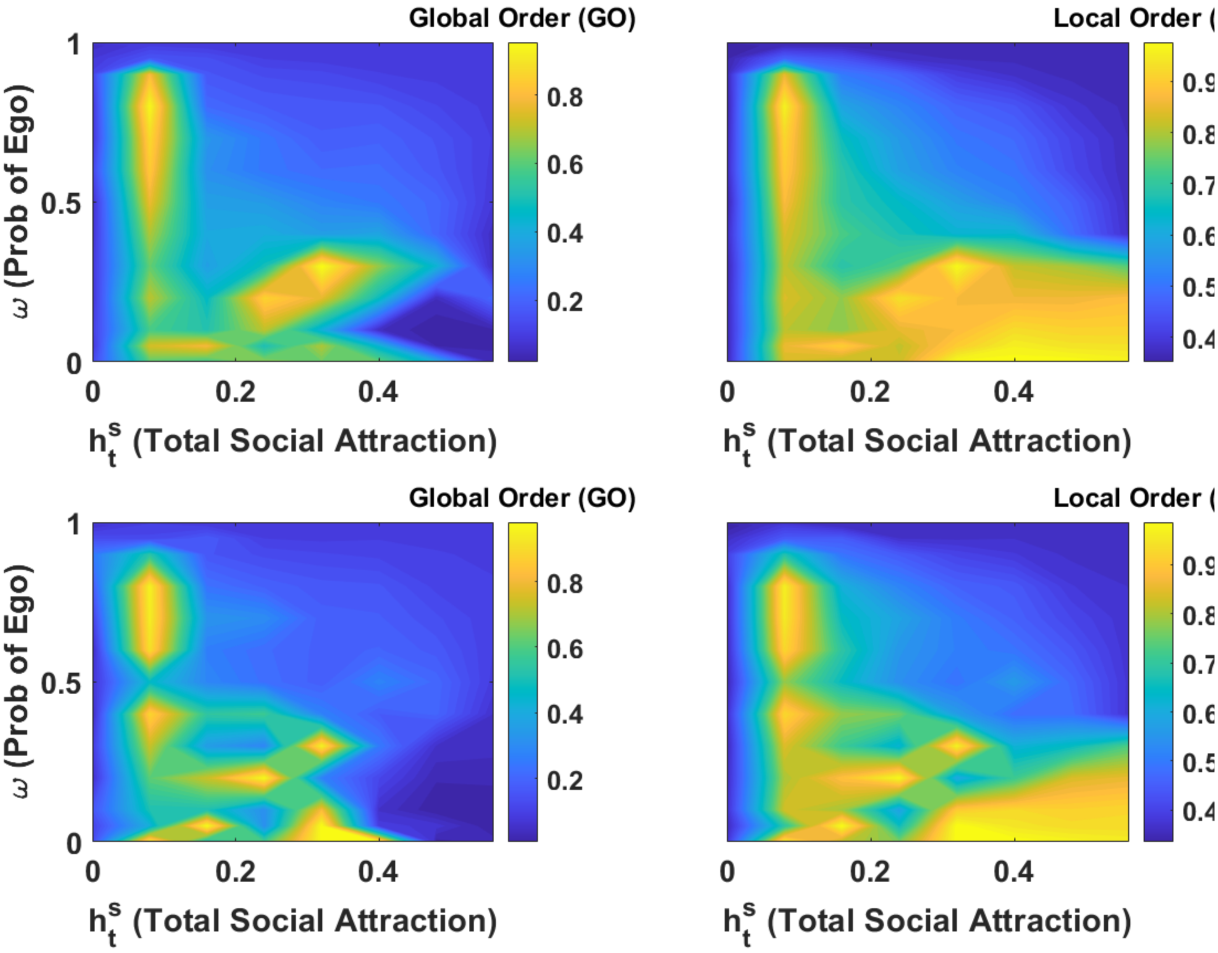

Supplementary Figure. 99: Contour plots of global and local order in $\omega - h_t^s$ plane in groups of 80 agents in the neural field model with switching between allocentric and egocentric representations of space. The top panels show an ensemble and a time average of over 5 simulations and the bottom panels show the time average results for only one simulation. A comparison of the two cases shows that, while in purely allocentric representation, ($\omega = 0$) in some runs high global order is observed, due to the multistability of different movement patterns, the system exhibits transitions between different patterns, both in time and in different runs. This complexity is reduced when agents switch between an allocentric and egocentric representation and a highly ordered collective motion becomes more stable, leading to high global and local order in the ensemble averages. Parameter values: $N_s = 100$, $\sigma = 0.4$, $v_0 = 0.05$, $dt = 0.3$, $h_b = 0$, and $\beta = 1000$. 80 agents move in a periodic space with linear size, $L = 1000$.

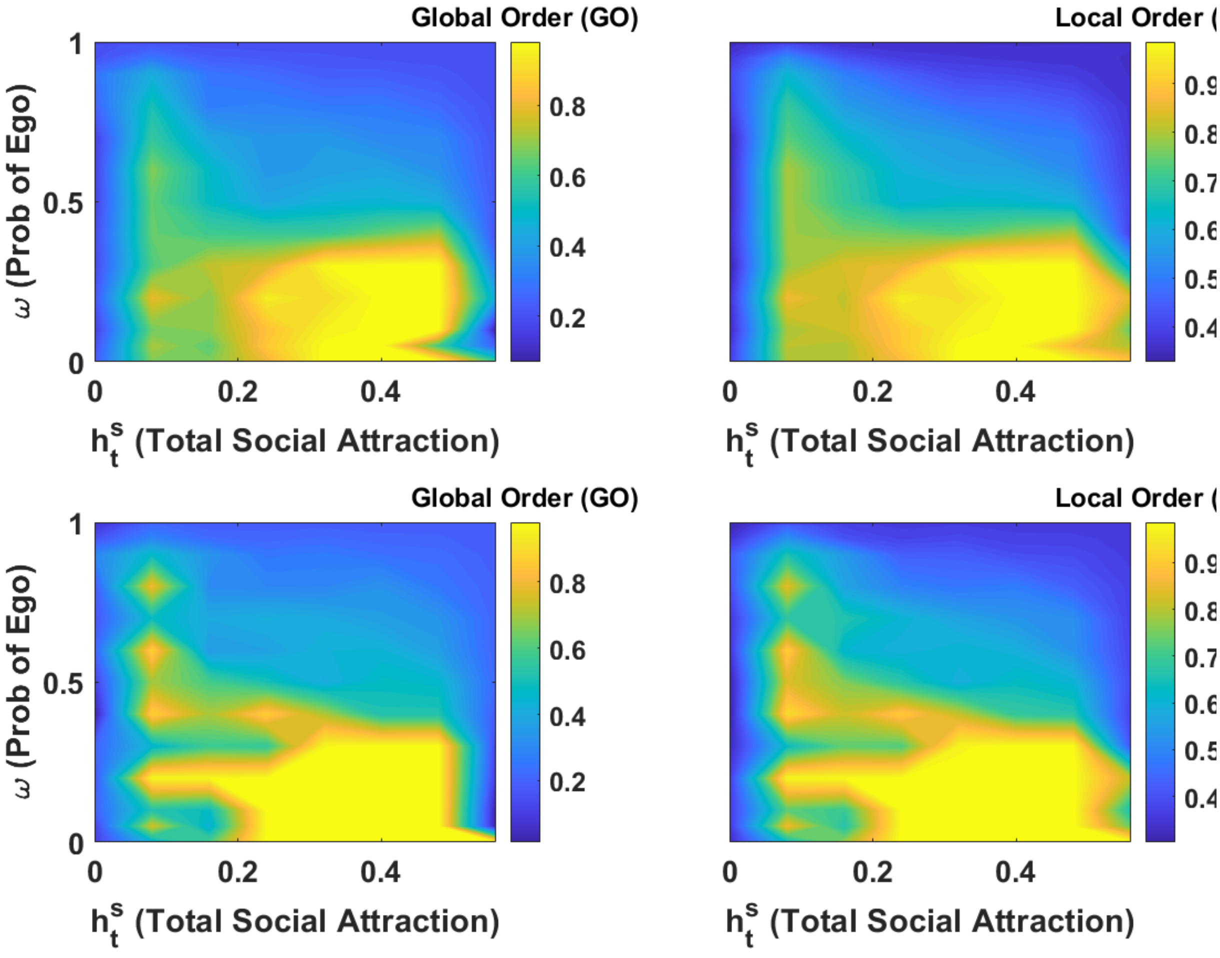

Supplementary Figure. 100: Contour plots of global and local order in $\omega - h_t^s$ plane in groups of 20 agents in the neural field model with switching between allocentric and egocentric representations of space. The top panels show an ensemble and a time average of over 5 simulations and the bottom panels show the time average results for only one simulation. A comparison of the two cases shows that, while in purely allocentric representation, ($\omega = 0$) in some runs high global order is observed, due to the multistability of different movement patterns, the system exhibits transitions between different patterns, both in time and in different runs. This complexity is reduced when agents switch between an allocentric and egocentric representation and a highly ordered collective motion becomes more stable, leading to high global and local order in the ensemble averages. Parameter values: $N_s = 100$, $\sigma = 0.4$, $v_0 = 0.05$, $dt = 0.3$, $h_b = 0$, and $\beta = 1000$. 20 agents move in a periodic space with linear size, $L = 1000$.

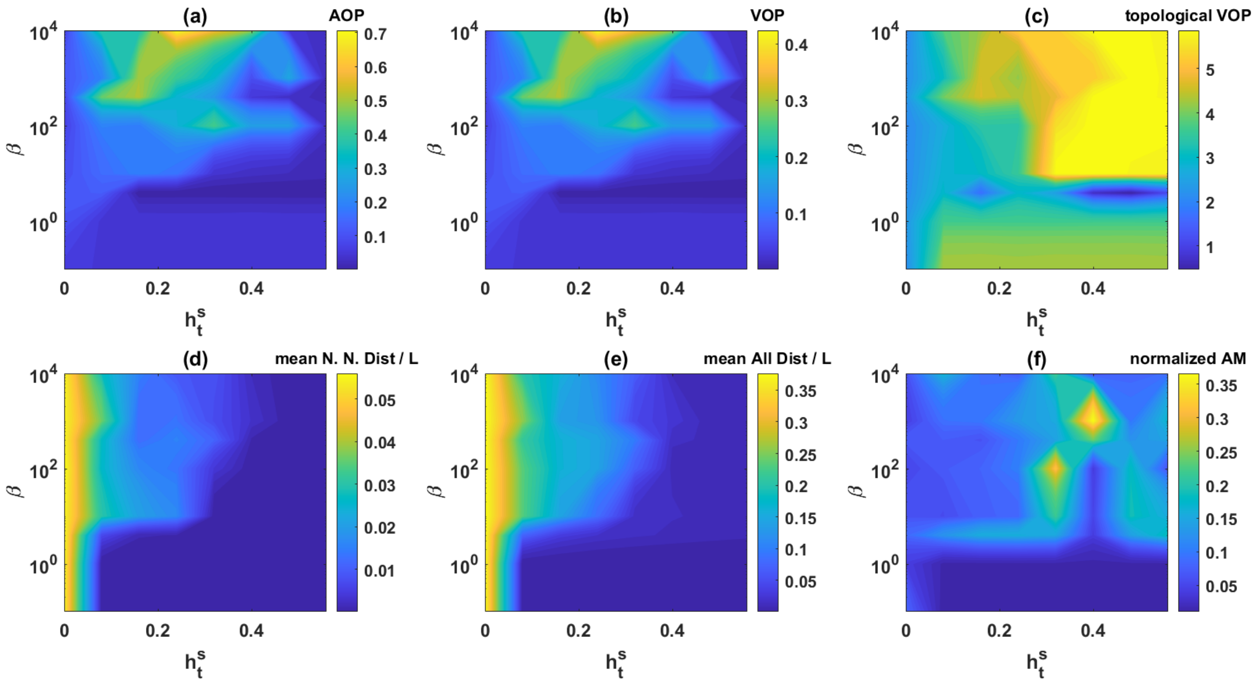

Supplementary Figure. 101: Contour plots of collective motion metrics in $\beta - h_t^s$ space in groups of 80 agents in the neural field model with an allocentric representation of space and in the modification of the neural field model based on self-organized head direction adjustment. The collective motion order parameters are color plotted as a function of the social attraction (h_s^0) and inverse neural noise (β). For a social attraction that is too small, the agents move independently. As social attraction increases, the collective first shows a phase transition to a collective motion phase with high global (global angular order parameter (AOP in (a)) and vectorial order parameter (VOP in (b))) and local (topological VOP in (c)) order, and then a second phase transition to an aggregation phase with high local order but often small global order and low mean distance between agents (mean nearest neighbor distance (d) and the distance between all pairs (e)). While the agents coalesce for high social attraction, the group of packed agents often shows a small collective motion for too high social attraction. A relatively high (time-averaged) normalized angular momentum (f) indicates milling patterns are observed in the neural field model. Parameter values: $N_s = 100$, $\sigma = 0.4$, $v_0 = 0.05$, $dt = 0.3$, $\nu = 0.5$, $h_b = 0$. 80 agents move in a periodic space with linear size, $L = 1000$.

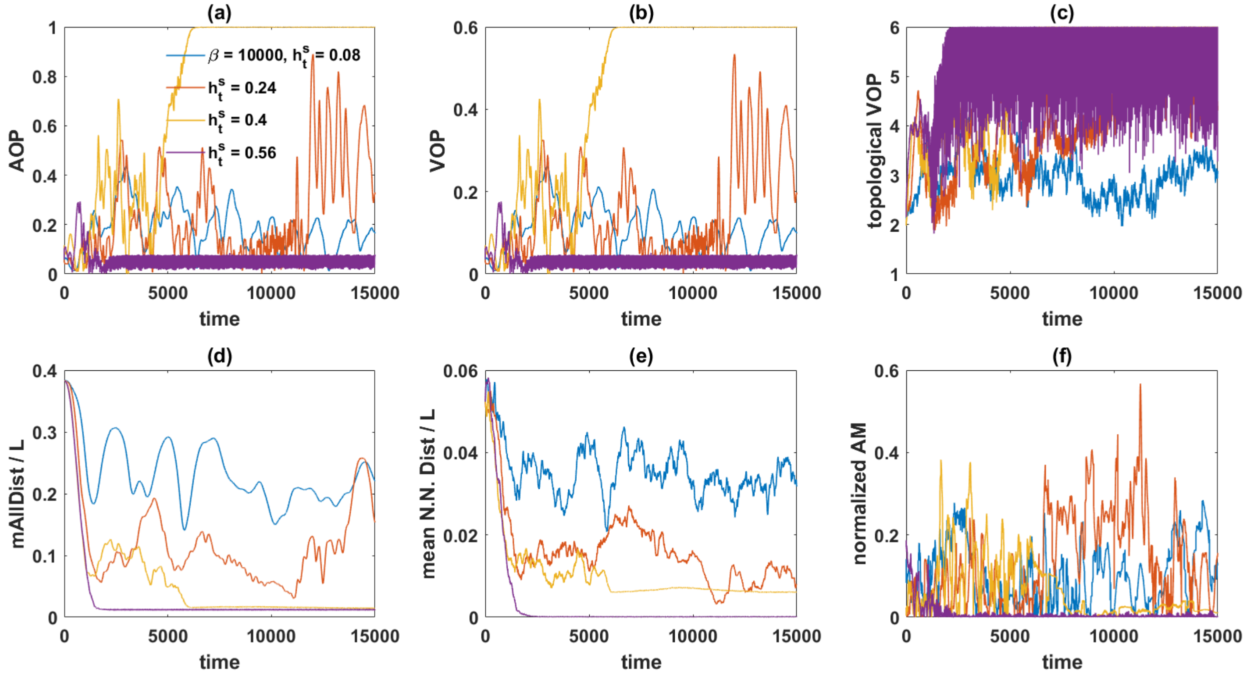

Supplementary Figure. 102: Time dependence of collective movement in groups of 80 agents with an allocentric representation of space in the neural field model with constant speed and self-organized head direction. Angular order parameter (AOP in (a)), vectorial order parameter (VOP in (b)), topological VOP (c), the mean distance between all the pairs (normalized by the arena size, L, mAllDist/L in (d)), mean nearest neighbor distance (N.N. Dist/L in (e)), and normalized angular momentum, (AM in (f)) for four different values of total social attraction indicated in the legend as a function of time is shown. In the absence of social attraction, individuals do not interact and no collective motion is observed. For moderate values of total social attraction, collective motion is observed. For larger values of social attraction, $h_s^0 = 0.4$, cohesive motion with low global order and low distance between individuals is observed. Parameter values: $N_s = 100$, $\sigma = 0.4$, $v_0 = 0.05$, $h_b = 0$, $dt = 0.3$, $\nu = 0.5$ and $\beta = 10000$. $g = 80$ agents move in a periodic space with linear size, $L = 1000$.

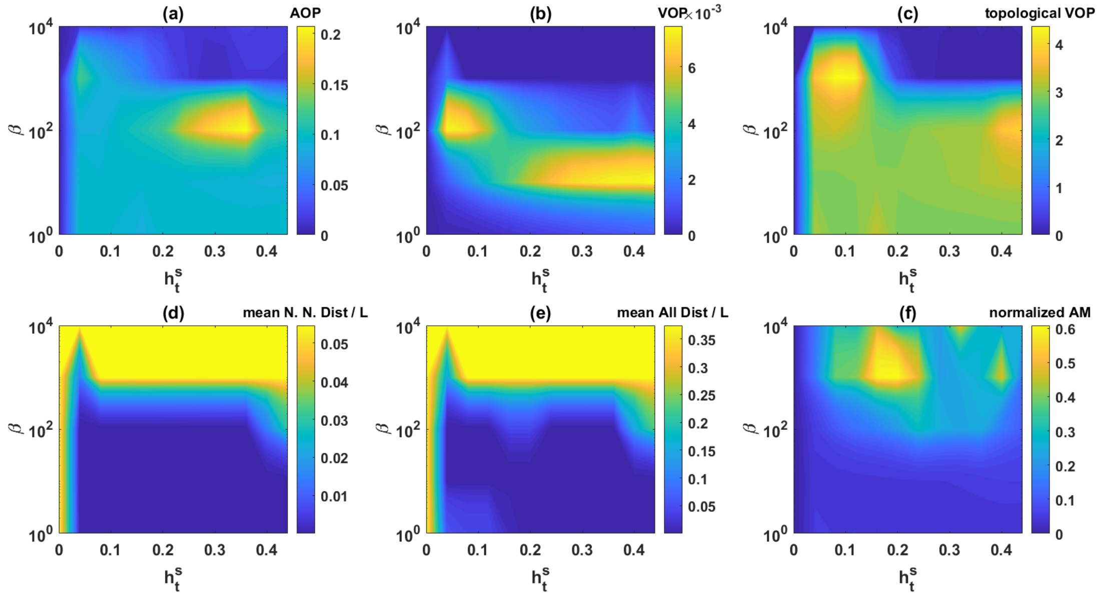

Supplementary Figure. 103: Contour plots of collective motion metrics in $\beta - h_t^s$ space in groups of 80 agents in the neural field model with an egocentric representation of space and with no recurrent connections. The collective motion order parameters are color plotted as a function of the social attraction (h_s^0) and inverse neural noise (β). For too large β, the individuals do not move or move too slowly for the simulations to reach a stationary state. For smaller values of β, the individuals move toward each other and form dense aggregates. The aggregate only shows slow random-walk-like movement with low global and local order. Parameter values: $N_s = 100$, $\sigma = 0.4$, $v_0 = 0.05$, $dt = 0.3$, $h_b = 0$. 80 agents move in a periodic space with linear size, $L = 100$.

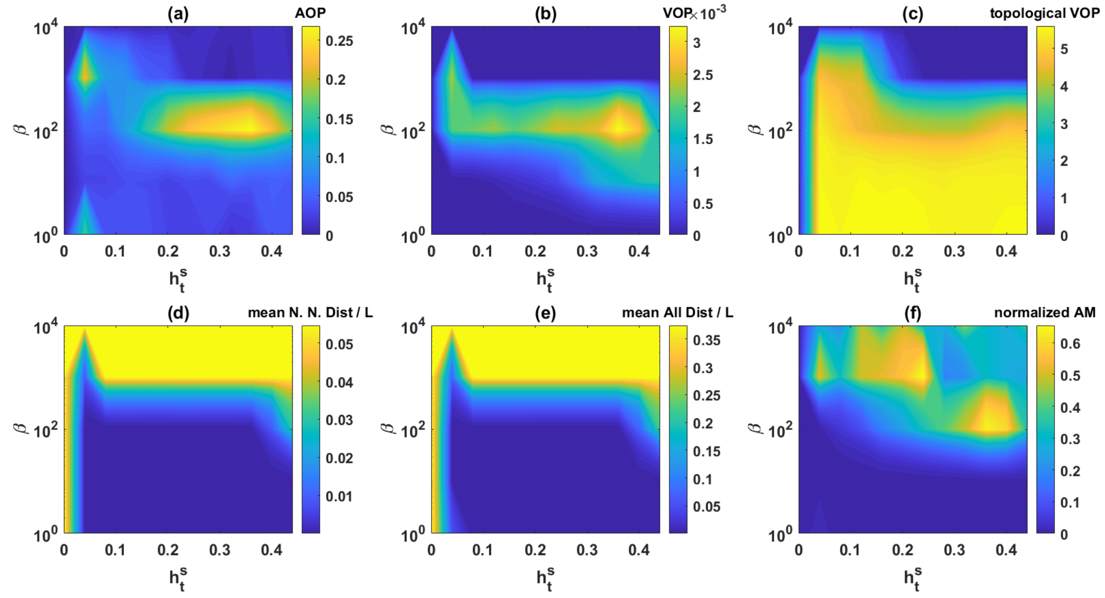

Supplementary Figure. 104: Contour plots of collective motion metrics in $\beta - h_t^s$ space in groups of 80 agents in the neural field model with an allocentric representation of space and with no recurrent connections. The collective motion order parameters are color plotted as a function of the social attraction (h_s^0) and inverse neural noise (β). For too large β, the individuals do not move or move too slowly for the simulations to reach a stationary state. For smaller values of β, the individuals move toward each other and form dense aggregates. The aggregate only shows slow random-walk-like movement with low global order but relatively high local order. Parameter values: $N_s = 100$, $\sigma = 0.4$, $v_0 = 0.05$, $dt = 0.3$, $h_b = 0$. 80 agents move in a periodic space with linear size, $L = 100$.

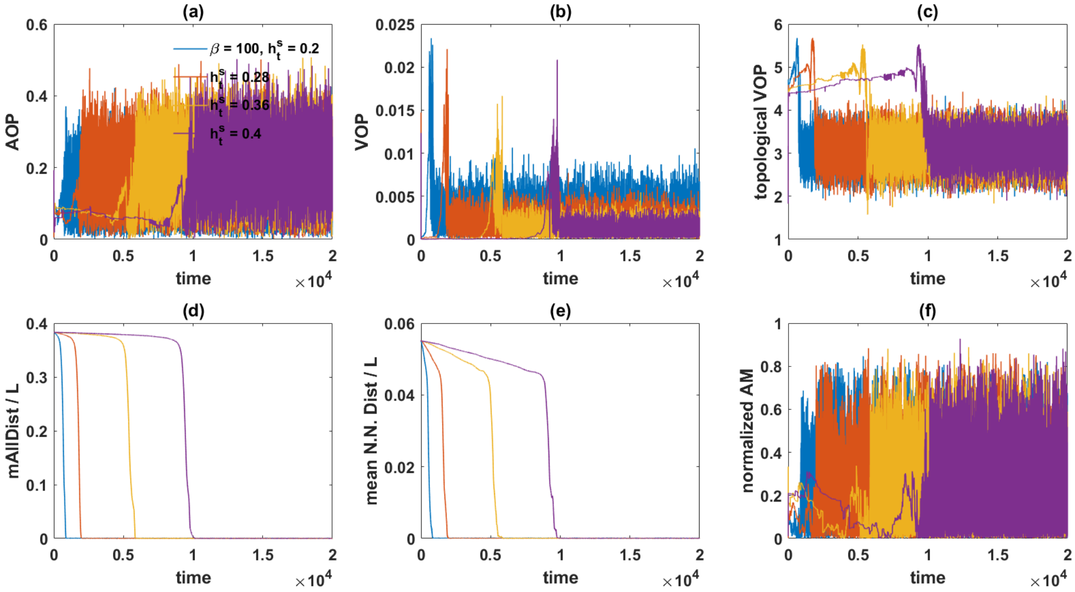

Supplementary Figure. 105: Time dependence of collective movement in groups of 80 agents with an egocentric representation of space in the neural field model with no recurrent connections. Angular order parameter (AOP in (a)), vectorial order parameter (VOP in (b)), topological VOP (c), the mean distance between all the pairs (normalized by the arena size, L, mAllDist/L in (d)), mean nearest neighbor distance (N.N. Dist/L in (e)), and normalized angular momentum, (AM in (f)) for four different values of total social attraction indicated in the legend as a function of time is shown. The acceleratingly fast, decreasing distance between agents over time shows the collapse of the collectives into a dense state. Once the collapse occurs, individuals show slow random-walk-like movements, indicated by high fluctuation in the global and local order and normalized angular momentum. Parameter values: $N_s = 100$, $\sigma = 0.4$, $v_0 = 0.05$, $h_b = 0$, $dt = 0.3$, $\nu = 0.5$ and $\beta = 100$. $g = 80$ agents move in a periodic space with linear size, $L = 100$.

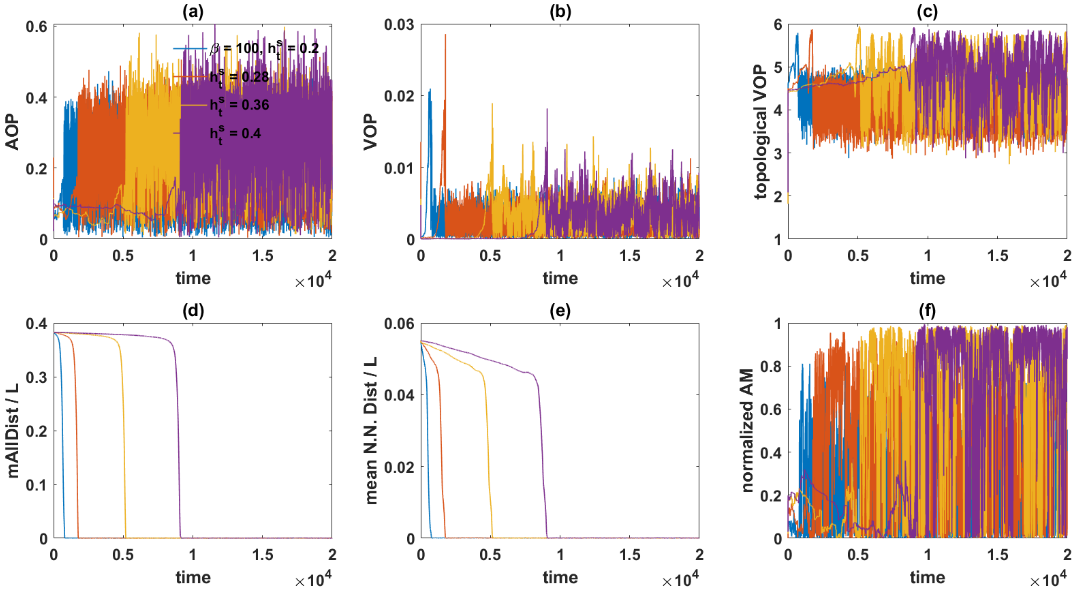

Supplementary Figure. 106: Time dependence of collective movement in groups of 80 agents with an allocentric representation of space in the neural field model with no recurrent connections. Angular order parameter (AOP in (a)), vectorial order parameter (VOP in (b)), topological VOP (c), the mean distance between all the pairs (normalized by the arena size, L, mAllDist/L in (d)), mean nearest neighbor distance (N.N. Dist/L in (e)), and normalized angular momentum, (AM in (f)) for four different values of total social attraction indicated in the legend as a function of time is shown. The acceleratingly fast, decreasing distance between agents over time shows the collapse of the collectives into a dense state. Once the collapse occurs, individuals show slow random-walk-like movements, indicated by high fluctuation in the global and local order and normalized angular momentum. Parameter values: $N_s = 100$, $\sigma = 0.4$, $v_0 = 0.05$, $h_b = 0$, $dt = 0.3$, $\nu = 0.5$ and $\beta = 100$. $g = 80$ agents move in a periodic space with linear size, $L = 100$.
