# Supplementary material for: Allocentric flocking
Source: Nat Commun. 2025 Oct 13;16:9051. doi: 10.1038/s41467-025-64676-5 (PMC12518777; doi:10.1038/s41467-025-64676-5)
Supplement: Supplementary file 2 — Description of Additional Supplementary Files [file 41467_2025_64676_MOESM2_ESM.pdf]

## Description of Additional Supplementary Files

File Name: Supplementary Video 1.
Description: Collective motion of allocentric agents in small groups (*N=10* agents) and close to the order-disorder transition in the spin system model. While global order exists, the group shows a variety of motion patterns, including intermittent swirling, sudden direction change, and fission-fusion dynamics. Parameter values: $N_s = 100, v_0 = 10, \sigma = \frac{2\pi}{N_s}, \beta = 400, N = 10, h_t^s = 0.024, h_b = 0$, and $L = 1000$.

File Name: Supplementary Video 2.
Description: Collective motion of allocentric agents in small groups (*N=10* agents) and in the ordered phase in the spin system model. While global order exists, the group shows a variety of motion patterns, including intermittent swirling, sudden direction change, and fission-fusion dynamics. Parameter values: $N_s = 100, v_0 = 10, \sigma = \frac{2\pi}{N_s}, \beta = 400, N = 10, h_t^s = 0.16, h_b = 0$, and $L = 1000$.

File Name: Supplementary Video 3.
Description: Collective motion of allocentric agents in small groups (*N=10* agents) in the spin system model. The group is close to the phase transition between the collective motion and aggregation phase, and the intermittency between these two modes of motion can be observed. Parameter values: $N_s = 100, v_0 = 10, \sigma = \frac{2\pi}{N_s}, \beta = 400, N = 10, h_t^s = 0.28, h_b = 0$, and $L = 1000$.

File Name: Supplementary Video 4.
Description: Collective motion of allocentric agents in large groups (*N=320* agents) and close to the order-disorder transition in the spin system model. Collective motion with high global order can be observed. Parameter values: $N_s = 100, v_0 = 10, \sigma = \frac{2\pi}{N_s}, \beta = 400, N = 320, h_t^s = 0.02, h_b = 0$, and $L = 1000$.

File Name: Supplementary Video 5.
Description: Collective motion of allocentric agents in large groups (*N=320* agents) in the spin system model. Collective motion with high global order and intermittency and coexistence of different modes of motion, such as fission-fusion dynamics, swirling, startling, and sudden direction changes can be observed. Parameter values: $N_s = 100, v_0 = 10, \sigma = \frac{2\pi}{N_s}, \beta = 400, N = 320, h_t^s = 0.032, h_b = 0$, and $L = 1000$.

File Name: Supplementary Video 6.
Description: Collective motion of allocentric agents in large groups (*N=320* agents) in the spin system model. A variety of collective motions can be observed. Explosive and implosive motion of the group leads to highly coordinated state changes between different motion patterns. Parameter values: $N_s = 100, v_0 = 10, \sigma = \frac{2\pi}{N_s}, \beta = 400, N = 320, h_t^s = 0.08, h_b = 0$, and $L = 1000$.

File Name: Supplementary Video 7.
Description: Collective motion of allocentric agents in large groups (*N=320* agents) and close to the transition between the collective motion and aggregation phase in the spin system model. A variety of collective motion patterns can be observed. Explosive and implosive motion of the group leads to highly coordinated state changes between different motion patterns. Parameter values: $N_s = 100, v_0 = 10, \sigma = \frac{2\pi}{N_s}, \beta = 400, N = 320, h_t^s = 0.12, h_b = 0$, and $L = 1000$.

File Name: Supplementary Video 8.

Description: Collective motion of allocentric agents in large groups (*N=320* agents) at the transition between collective motion and aggregation phase in the spin system model. Explosive and implosive motion of the group close to the aggregation phase transition can be observed. Parameter values: $N_s = 100, v_0 = 10, \sigma = \frac{2\pi}{N_s}, \beta = 400, N = 320, h_t^s = 0.16, h_b = 0$, and $L = 1000$.

File Name: Supplementary Video 9.
Description: Supplementary Video 9 shows an example of collective motion patterns in the neural field model in the collective motion phase for small $h_t^s$, characterized by unstable dynamics and intermittency between different movement patterns, such as directed motion, flash expansion, sudden direction change, and fission-fusion dynamics. Parameter values: $N_s = 100, v_0 = 5, \sigma = 0.4, \beta = 1000, N = 80, h_t^s = 0.08, h_b = 0$, $\Delta t = 0.3$ and $L = 1000$.

Supplementary Video 10.
Description: Supplementary Video 10 shows an example of collective motion patterns in the neural field model and in the collective motion phase. The population shows transient motion between a variety of patterns, such as milling, moving bands, and fission-fusion dynamics. Parameter values: $N_s = 100, v_0 = 5, \sigma = 0.4, \beta = 1000, N = 80, h_t^s = 0.24, h_b = 0$, $\Delta t = 0.3$ and $L = 1000$.

Supplementary Video 11.
Description: Supplementary Video 11 shows an example of collective motion patterns in the neural field model and in the collective motion phase, but closer to the aggregation phase transition. The population shows transient motion between a variety of patterns, such as milling, moving bands, and fission-fusion dynamics. Parameter values: $N_s = 100, v_0 = 5, \sigma = 0.4, \beta = 1000, N = 80, h_t^s = 0.28, h_b = 0$, $\Delta t = 0.3$ and $L = 1000$.

File Name: Supplementary Video 12.
Description: Supplementary Video 12 shows collective motion patterns close to the collective motion-aggregation phase transition in the neural field model. The system spends more time in a state where the population is composed of subgroups of coherently moving individuals, with high alignment, exhibiting directed motion. Parameter values: $N_s = 100, v_0 = 5, \sigma = 0.4, \beta = 1000, N = 80, h_t^s = 0.32, h_b = 0$, $\Delta t = 0.3$ and $L = 1000$.

File Name: Supplementary Video 13.
Description: Supplementary Video 13 shows collective motion patterns in large groups of *N=320* individuals in the neural field model in the collective motion phase. Intermittency between a variety of collective motion patterns, from milling to fission-fusion, flash expansion, sudden direction change, and highly ordered motion is observed. Parameter values: $N_s = 100, v_0 = 5, \sigma = 0.4, \beta = 1000, N = 320, h_t^s = 0.24, h_b = 0$, $\Delta t = 0.3$ and $L = 1000$.

File Name: Supplementary Video 14.
Description: Supplementary Video 14 shows collective motion patterns in large groups of *N=320* individuals in the neural field model in the collective motion phase but closer to the collective motion-aggregation phase transition. Here, after an initial period of mostly milling, the population forms subgroups of mobile aggregates moving with high order. Parameter values: $N_s = 100, v_0 = 5, \sigma = 0.4, \beta = 1000, N = 320, h_t^s = 0.28, h_b = 0$, $\Delta t = 0.3$ and $L = 1000$.

File Name: Supplementary Video 15.
Description: Supplementary Video 15 shows an example of collective motion when individuals randomly employ an allocentric and an egocentric perception of space. Here the neural field model is used and $h_t^s = 0.08$, corresponding to SV.9, where the same parameter values, but for a purely allocentric perception of space are used. Here, at each time step, individuals randomly employ an

allocentric or egocentric perception of space. The probability of being in the egocentric state is $\omega = 0.8$, maximizing the global order. Switching between the two states stabilizes highly ordered collective motion. Although such movement patterns are also observed when individuals employ a purely allocentric perception of space, these patterns are not stable in the absence of a switch to an egocentric state. Parameter values: $N_s = 100, v_0 = 5, \sigma = 0.4, \beta = 1000, N = 80, h_t^s = 0.08, h_b = 0$, $\Delta t = 0.3$ $\omega = 0.8$, and $L = 1000$.

File Name: Supplementary Video 16.

Description: Supplementary Video 16 shows collective motion patterns for $h_t^s = 0.4$ and $\omega = 0.2$. With a purely allocentric perception of space, in this regime, often milling is observed. Such a milling pattern can be seen in the initial times in the video. However, a small probability of being in the egocentric state destabilizes milling and leads to highly ordered motion of moving aggregates Parameter values: $N_s = 100, v_0 = 5, \sigma = 0.4, \beta = 1000, N = 80, h_t^s = 0.4, h_b = 0$, $\Delta t = 0.3$ $\omega = 0.2$, and $L = 1000$.

File Name: Supplementary Video 17.

Description: Supplementary Video 17 shows the dynamics of the model in the absence of recurrent connections and with an allocentric perception of space. Here, the same parameter values as in SV.9 to SV.16 are used. However, $\beta = 100$ and $h_t^s = 0.36$, and $L = 100$. To remove the recurrent connections, using the neural field model, we have set $J_{ij} = 0$ for all *i* and *j*. This ensures all the aspects of the model are preserved. However, the dynamics of the system are the result of feedforward connections. As can be seen in the Video, starting from random initial positions, agents move toward each other in an accelerating fashion and coalesce in the same position with slow, random-walk-like movement. This shows that recurrent connections are essential for the rich dynamical patterns observed in the model. Parameter values: $N_s = 100, v_0 = 5, \sigma = 0.4, \beta = 100, N = 80, h_t^s = 0.36, h_b = 0$, $\Delta t = 0.3$ $\omega = 0.2$, and $L = 100$.

File Name: Supplementary Data 1.

Description: Supplementary Data 1 contains the MATLAB codes used for the simulation of the neural field model.
